# MATR3 is essential for oocyte growth and maturation quality through a dual molecular mechanism

**DOI:** 10.7554/eLife.110703

**Published:** 2026-09-21

**Authors:** Yibing Bao, Zhenzi Zuo, Tengteng Wang, Lin Lin, Meng Gao, Shaogang Qin, Qingfeng Yang, Bingying Liu, Wanyuan Sun, Jie Ma, Tianhua Zhu, Guoliang Xia, Bo Zhou, Rong Hu, Hua Zhang, Fengchao Wang, Chao Wang

**Affiliations:** 1 State Key Laboratory of Animal Biotech Breeding, College of Biological Sciences, China Agricultural University Beijing China; 2 Department of Physiology and Pathophysiology, National Key Discipline of Cell Biology, School of Basic Medicine, Fourth Military Medical University Xi’an China; 3 College of Bioscience and Resources Environment, Beijing University of Agriculture Beijing China; 4 Department of Gynecology, General Hospital of Ningxia Medical University Ningxia China; 5 Reproductive Medicine Center, General Hospital of Ningxia Medical University Ningxia China; 6 Institute of Medical Sciences, General Hospital of Ningxia Medical University Ningxia China; 7 Transgenic Animal Center, National Institute of Biological Sciences Beijing China; The University of Adelaide Australia; Washington State University United States

**Keywords:** Matrin-3, growing oocyte, epigenetic modification, follicle development, fertility, Mouse

## Abstract

The molecular mechanisms governing mRNA accumulation during oocyte growth, essential for developmental competence, remain poorly understood. This study investigates the role of Matrin-3 (MATR3), a highly expressed RNA-binding protein in growing oocytes (GOs), using oocyte-specific knockout mouse models and human oocyte maturation arrest (OMA) samples. The results showed that MATR3 was more abundant in GOs than fully grown oocytes (FGOs), highly expressed in the nucleus of non-surrounded nucleolus (NSN) oocytes, and exited the nucleus during the NSN-to-surrounded nucleolus (SN) transition. In OMA patients, MATR3 nuclear localization was missed, with smaller oocytes than FGOs. Further, *Matr3* deletion in mouse GOs caused restricted oocyte growth, global transcription disorders, follicle development failure, blocked GO-granulosa cell communication (via reduced *Gdf9* and *Rdx* expression), and infertility. Mechanistically, MATR3 regulated transcription by recruiting H3K9me2-demethylating lysine-specific demethylase 3B or binding target gene promoters, like *Rdx*. These findings reveal a critical role of MATR3 in orchestrating transcription and paracrine signaling during oogenesis and suggest its potential as a diagnostic and therapeutic target for OMA.

## Introduction

The growth of oocytes refers to the process in which growing oocytes (GOs) develop into fully grown oocytes (FGOs). Oocyte growth involves an increase in volume, as well as the accumulation of metabolic molecules, transcripts, and proteins. In vivo, FGOs determine the developmental potential of early embryos. A high-quality FGO derives from the GO in a growing follicle, which may experience dynamic gene transcription, as well as fine-tuned orchestration between the GO and the surrounding granulosa cells. In the clinic, besides genetically caused female infertility, uncovering the etiologies, especially epigenetic causative reasons, is equally pivotal for improving the oocyte maturation quality in vitro (Ren et al., 2017; Wang et al., 2019; He et al., 2021; Gao et al., 2024; Zhu et al., 2024). Patients who suffer from oocyte maturation arrest (OMA) are unable to retrieve mature oocytes during their repetitive in vitro fertilization (IVF) and intracytoplasmic sperm injection (ICSI) cycles (Liu et al., 2018). In the past, the importance of oocyte-specific RNA-binding proteins (RBPs), such as MARF1, LSM14B, and MTR4 in improving oocyte development, as well as their relationship with OMA, has been confirmed (Su et al., 2012; Su et al., 2021; Wu et al., 2025). However, it remains unclear how other RBPs coordinate epigenetic molecules contributing to transcribe and preserve massive mRNAs to direct the timely growth of GOs and growing follicles.

Multiple RBPs have been proven to closely correlate with OMA. The PATL2 protein is known to regulate the homeostasis of maternal mRNAs, while a whole exome sequencing study has identified four recessive variants of the *PATL2* gene in three OMA families (Wang et al., 2024b). Also, the compound heterozygous *ZFP36L2* mutations correlate with OMA (Wan et al., 2024). ZFP36L2 is crucial for regulating the degradation of maternal mRNAs and the maturation of mouse oocytes (Chousal et al., 2018). Despite these, it remains unclear if other RBPs contribute to female fertility as well. This study focuses on a conserved RBP Matrin-3 (MATR3) that contains two zinc-finger domains to specifically bind to DNA sequences rich in GC (Zeitz et al., 2009). The two RNA recognition motifs of MATR3 participate in the regulation of alternative splicing by specifically binding to mRNA sequences rich in AU (Zeitz et al., 2009; Iradi et al., 2018). Moreover, MATR3 is rich in nuclear localization sequences, which enable it to localize in the nucleus. One of the characteristic functions of MATR3 is that it recruits other RBPs while binding to the target sequences (Pollini et al., 2021). Importantly, as a powerful RBP, the abnormal aggregation and pathological mutations of MATR3 may interfere with normal cellular functions, including RNA stability and transport (Coelho et al., 2015; Cha et al., 2021). Unfortunately, whether and how MATR3 correlates with GO to FGO development and female fertility remains unknown.

The growth of growing follicles, as well as oocyte maturation, depends strictly on endocrine and paracrine regulations in vivo. Studies including ours have shown that during the cyclic recruitment of growing follicles in each estrus cycle, paracrine factors like CNP (C-type natriuretic peptide) coordinate gonadotropins to timely control FGO meiosis arrest, meiosis resumption, and ovulation (Zhang et al., 2010). However, throughout the progress from GO to FGO, cumulative studies have highlighted the critical roles of oocyte-secreted factors (OSFs) in supporting follicular somatic cell proliferation, metabolism, and GO development (Hanrahan et al., 2004; Su et al., 2008; Nicol et al., 2009; Silva et al., 2011; Gao et al., 2024). Of these, TGFβ superfamily members, growth differentiation factor 9 (GDF9) and bone morphogenic protein 15 (BMP15), are the most studied ones (Dong et al., 1996; Dube et al., 1998). Within a growing follicle, GDF9 functions by regulating the cuboidal transformation and promoting the proliferation of granulosa cells, which has been repetitively demonstrated by studies, including ours (Carabatsos et al., 1998; Spicer et al., 2008; Gao et al., 2024). BMP15, highly homologous to GDF9, regulates the development of early growing follicles by controlling the proliferation of granulosa cells through the regulation of the GDF9-SMAD signaling pathway (Gilchrist et al., 2008). Most recently, we have proved that the major function of oocyte-derived mushroom-like microvilli (Oo-Mvi) relates to the release of OSFs timely and orderly. Loss of RADIXIN (RDX), the key component of Oo-Mvi, resulted in a failure of the formation of Oo-Mvi and a shortened reproductive lifespan in females (Zhang et al., 2021). However, how RBPs regulate OSFs, such as GDF9 expression and secretion via Oo-Mvi, remains uncertain.

Results of this study showed that MATR3 is highly expressed in the GOs of mice, pigs, and humans, indicating a conserved and essential function. MATR3 is required for female fertility, as its specific deletion in mouse GOs severely reduced antral follicle (AF) formation and resulted in infertility. MATR3 regulates transcription by recruiting H3K9me2-demethylating lysine-specific demethylase 3B or binding target gene promoters, such as *Rdx*. Conclusively, MATR3 is a strong candidate causative RBP contributing to OMA in females.

## Results

### MATR3 expression in oocyte of growing follicle is required for female fertility

**Figure 1.**
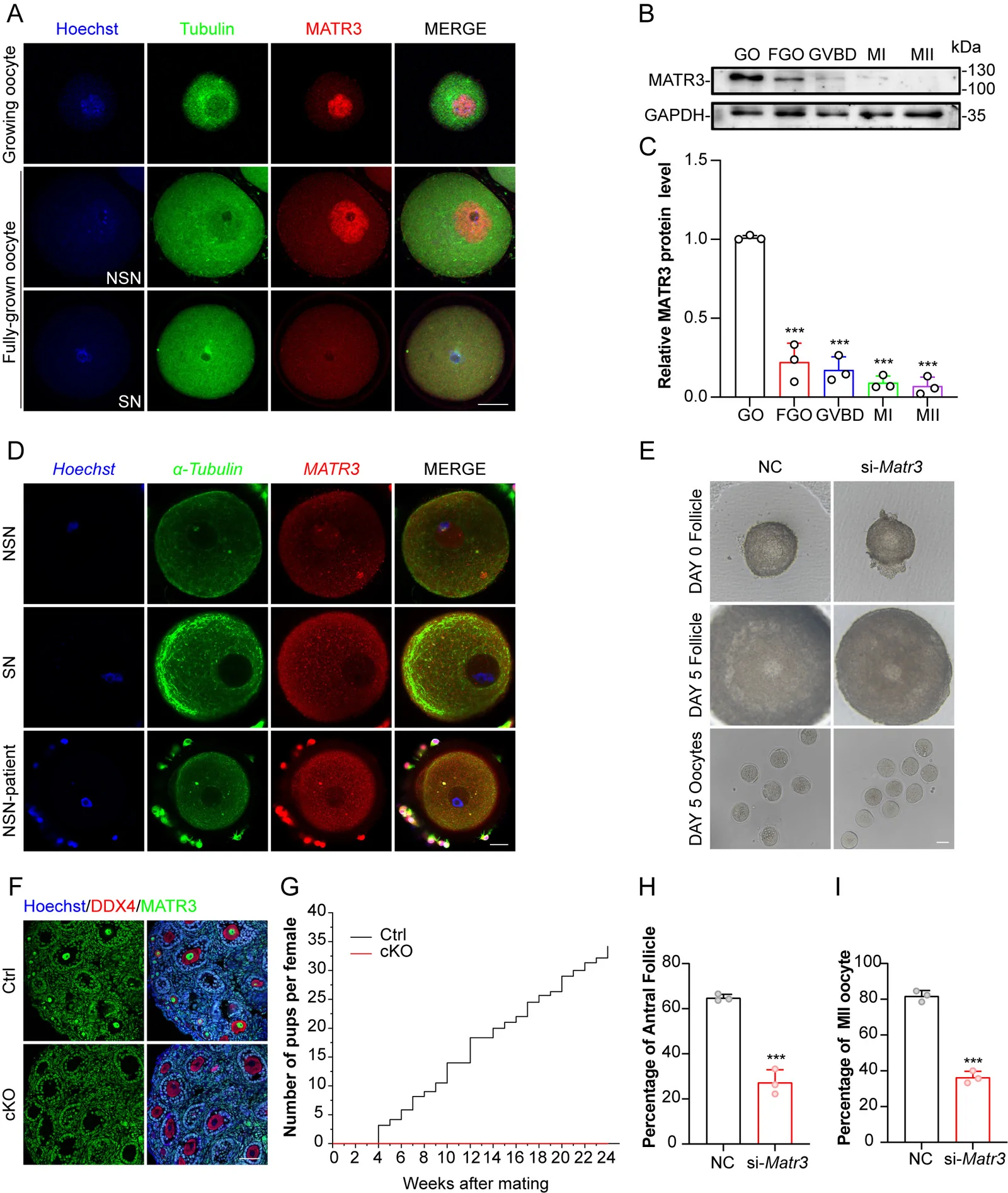
MATR3 in growing oocyte regulates female reproduction in mice. (**A**) Immunofluorescence showing MATR3 levels in mouse oocytes. (**B**) Western blot results showing MATR3 expression in mouse oocytes. Total proteins from 200 oocytes were loaded in each lane. GAPDH served as a loading control. (**C**) Relative expression level of MATR3 to GAPDH. (**D**) Immunofluorescence showing MATR3 levels in human oocytes. n=11. (**E**) Representative image of follicles and oocytes from negative control (NC) and si-*Matr3*. (**F**) Immunohistochemistry results showing MATR3 expression in oocytes. (**G**) Cumulative number of pups from Ctrl (n=6) and cKO (n=3) female. (**H**) Percentage of antral follicles in **E**. n≥33 per group. (**I**) MII ratio of oocytes collected from **E**. n≥33 per group. Scale bars: 20 μm in (**A**), (**B**), and (**C**), 40 μm in (**E**) and (**F**). Data are represented as mean ± SD. ***p<0.001, **p<0.01, *p<0.05, n.s., not significant. Figure 1—source data 1.PDF file containing original western blots for Figure 1B, indicating the relevant bands and treatments. Figure 1—source data 2.Original files for western blot analysis displayed in Figure 1B. Figure 1—source data 3.Source data for Figure 1C, G, H and I.

To investigate the potential function of MATR3 in female reproduction, we examined its location and expression pattern during folliculogenesis in multiple mammals. The results showed that MATR3 was generally expressed in the nucleus of oocytes and somatic cells of mice (Figure 1A, Figure 1—figure supplement 1A). Specifically, the level of MATR3 in GOs remained at relatively higher levels than those found in the FGOs, as well as stages after maturation (Figure 1B and C), implying that MATR3 may be more important for supporting mouse GO growth. Interestingly, compared to the normal group in which the expression pattern of MATR3 is similar to that of mouse, we noticed a decrease in MATR3 protein level in FGO (non-surrounded nucleolus [NSN]) from a patient with OMA, which has not been reported before (Figure 1D). This finding suggests that MATR3 may be one of a candidate responsible for OMA. Similarly, we also detected the expression pattern of MATR3 in the ovaries of porcine, which is highly consistent with that in mice and humans, indicating that the physiological function of MATR3 may be highly conserved among species (Figure 1—figure supplement 1B–D).

**Figure 1—figure supplement 1.**
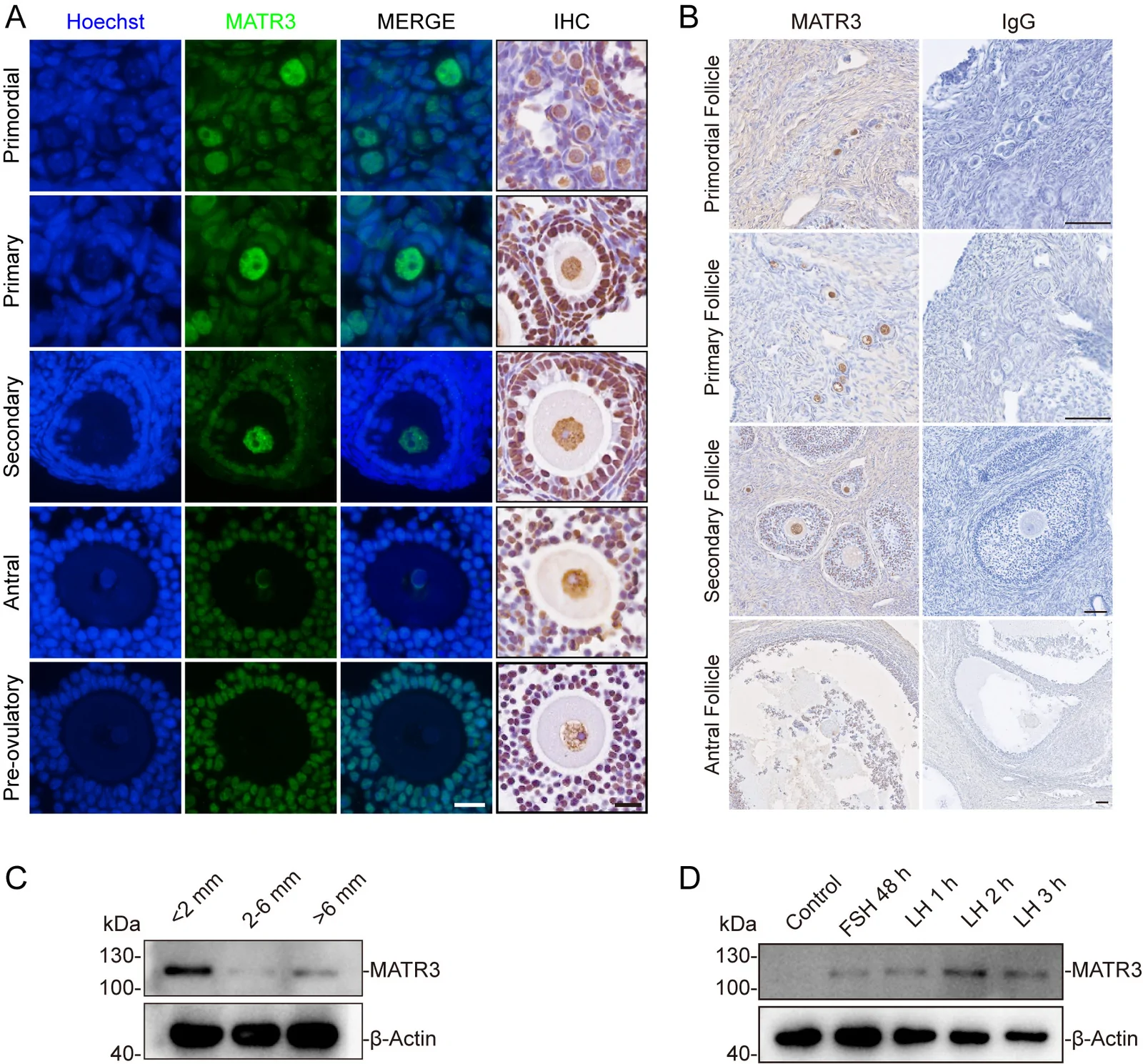
The expression pattern of MATR3 in ovaries of mice and porcine. (**A**) Immunofluorescence results showing MATR3 location in mouse ovaries. Mouse ovaries were stained for MATR3 (green) at postnatal day 23 (PD23). The nuclei were dyed with a Hoechst counterstain (blue). Scale bar: 25 μm. (**B**) Immunohistochemistry results showing MATR3 location in porcine ovaries. MATR3 was mainly localized to the nuclei of oocytes in either mouse or porcine. Scale bar: 50 μm. (**C**) Western blotting was used to detect the changes of MATR3 in GCs of porcine during the growth of follicles under physiological conditions. The results showed that MATR3 was highly expressed in the GCs of small secondary follicles, n=3. (**D**) Western blotting was used to detect the changes of MATR3 in GCs of porcine with the duration of LH. The results showed that MATR3 increased with the duration of LH, n=3. Figure 1—figure supplement 1—source data 1.PDF file containing original western blots for Figure 1—figure supplement 1C, indicating the relevant bands and treatments. Figure 1—figure supplement 1—source data 2.Original files for western blot analysis displayed in Figure 1—figure supplement 1C. Figure 1—figure supplement 1—source data 3.PDF file containing original western blots for Figure 1—figure supplement 1D, indicating the relevant bands and treatments. Figure 1—figure supplement 1—source data 4.Original files for western blot analysis displayed in Figure 1—figure supplement 1D.

**Figure 1—figure supplement 2.**
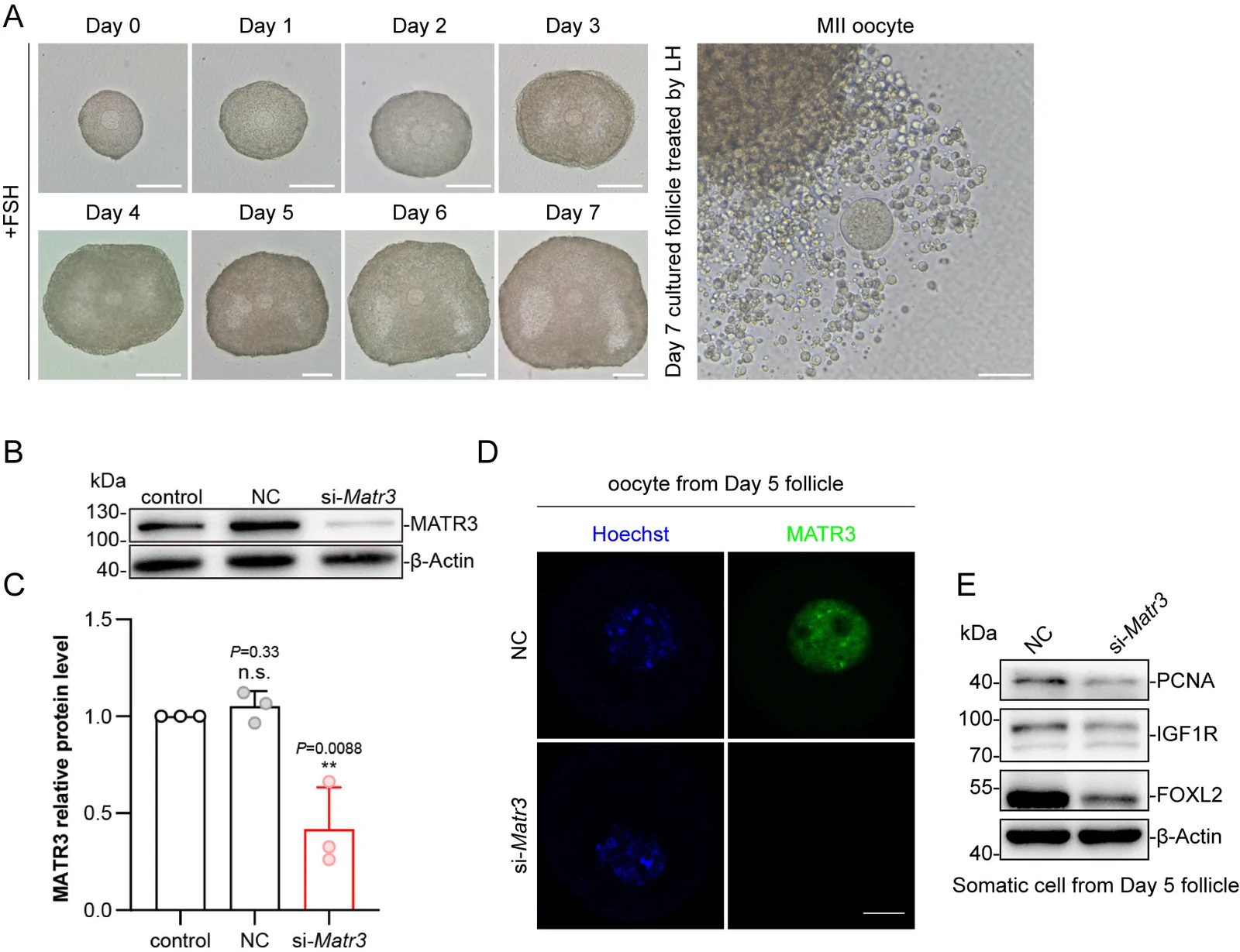
Construction of in vitro knockdown model, knockdown efficiency, and phenotypes. (**A**) Using the method of in vitro addition of follicle-stimulating hormone (FSH), secondary follicles with a diameter of 150–200 μm were continuously cultured. The follicles could develop to the pre-ovulatory follicle stage, and oocytes with the ability to resume meiosis could be ovulated 16 hr after the addition of LH. Scale bar: 100 μm. (**B**) After the 3T3 cell line was transfected with negative control (NC) and Matr3 siRNA, respectively, for 48 hr, western blot was used to detect the protein level of MATR3 in the cells, with n=3. β-Actin was used as an internal reference to normalize the protein loading level. (**C**) The statistical results of the gray scale scanning values of the MATR3 protein bands in (**B**). The statistical results are expressed as mean ± SD. (**D**) The knockdown efficiency was detected by immunofluorescence technique. Green fluorescence: MATR3; blue fluorescence: Hoechst (nucleus). Scale bar: 25 μm. (**E**) Isolate the somatic cells and oocytes of the follicles that have been continuously cultured for 5 days. Detect the changes in the levels of proteins related to the proliferation and differentiation of granulosa cells by western blotting. n=3. Figure 1—figure supplement 2—source data 1.PDF file containing original western blots for Figure 1—figure supplement 2B, indicating the relevant bands and treatments. Figure 1—figure supplement 2—source data 2.Original files for western blot analysis displayed in Figure 1—figure supplement 2B. Figure 1—figure supplement 2—source data 3.PDF file containing original western blots for Figure 1—figure supplement 2E, indicating the relevant bands and treatments. Figure 1—figure supplement 2—source data 4.Original files for western blot analysis displayed in Figure 1—figure supplement 2E. Figure 1—figure supplement 2—source data 5.Source data for Figure 1—figure supplement 2C.

**Figure 1—figure supplement 3.**
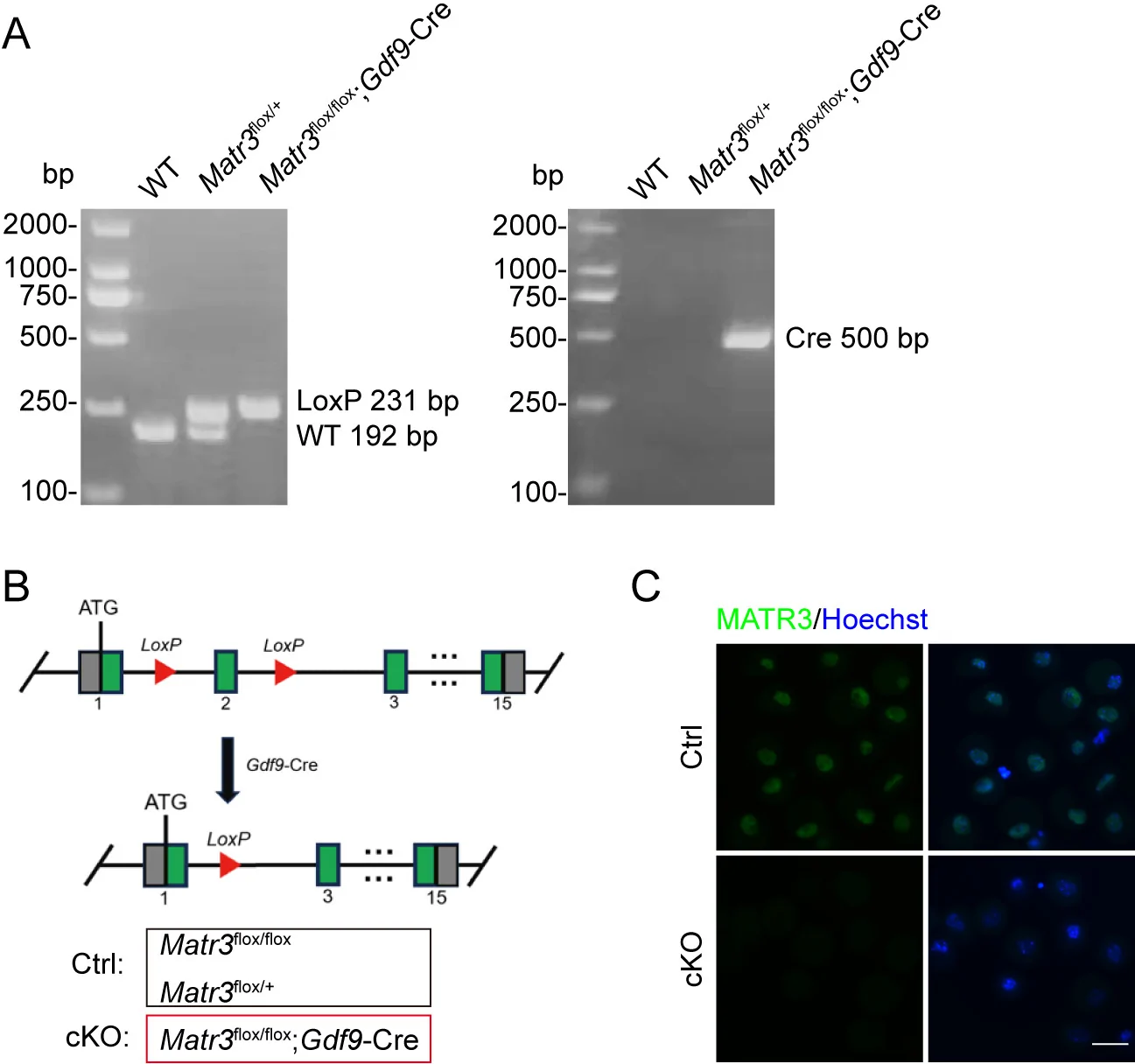
Strategies for constructing *Matr3* oocyte-specific knockout mice and detection of related phenotypes. (**A**) Using PCR to identify the genotypes of mice, with DL2000 as the DNA marker. After the insertion of LoxP, the size of the band is 231 bp, the size of the wild-type (WT) band is 192 bp, and the size of the Cre is 500 bp. (**B**) Construction strategy for *Matr3* knockout model in oocyte via *Gdf9*-Cre. ATG: transcription start site; red arrow: LoxP insertion site; numbers 1–15: exon regions 1–15 of *Matr3*. (**C**) Immunofluorescence results showing MATR3 location in oocytes from Ctrl and cKO mouse (postnatal day 14 [PD14]). Oocytes were stained for MATR3 (green). The nuclei were dyed with a Hoechst counterstain (blue). Scale bar: 50 μm. Figure 1—figure supplement 3—source data 1.PDF file containing original blots for Figure 1—figure supplement 3A, indicating the relevant bands and treatments. Figure 1—figure supplement 3—source data 2.Original files for blot analysis displayed in Figure 1—figure supplement 3A.

To explore the effect of MATR3 on GO growth, the authors first established an oocyte knockdown model of early growing follicles in mouse. This model enables the development of secondary follicles (SFs) into AFs and successful ovulation after gonadotropins hormone supplementation (Figure 1—figure supplement 2A). After injecting *Matr3* siRNA into the oocyte of SF with a diameter of 150 μm in the model, these follicles were in vitro cultured for 5–7 days (Figure 1—figure supplement 2B–D). Photographs were taken daily to record the follicular development status. On the fifth day, oocytes and granulosa cells were collected, respectively, to detect the extrusion rate of the first polar body of oocytes and multiple functional markers of granulosa cells. The results showed that after knocking down *Matr3* in the oocytes of SFs, follicular development was hindered. Specifically, when cultured in vitro for 5 days, the SFs in the negative control (NC) group could develop into AFs, while the proportion of AFs in the knockdown group (si-*Matr3*) was significantly reduced (Figure 1E and H, 64.87±1.531% vs 27.27±5.613%). Consistently, the extrusion rate of the first polar body of oocytes was significantly reduced (Figure 1I, 81.83±3.048% vs 36.35±3.380%). Meanwhile, the protein level of FOXL2 in granulosa cells decreased, the protein level of the proliferation marker PCNA decreased, and the protein level of the hormone-responsive receptor IGFIR decreased as well (Figure 1—figure supplement 2E).

Based on the above in vitro experiments, we utilized a novel mouse model with specific MATR3 deficiency in oocytes to explore the MATR3 function. Specifically, *Matr3* was conditionally knocked out after 3 dpp using *Gdf9*-Cre mice to generate *Matr3*^flox/flox^; *Gdf9*-Cre (cKO) mice and to evaluate its specific effect in oocytes (Figure 1—figure supplement 3A and B). The knockout efficiency of cKO mice was examined by immunofluorescence (Figure 1, Figure 1—figure supplement 3C). Notably, a fertility test showed that cKO mice were infertile during the 6-month mating process (Figure 1G). Thus, MATR3 in oocytes is crucial for the growth of follicles and the maintenance of fertility in mice.

### MATR3 is indispensable for supporting GO growth

**Figure 2.**
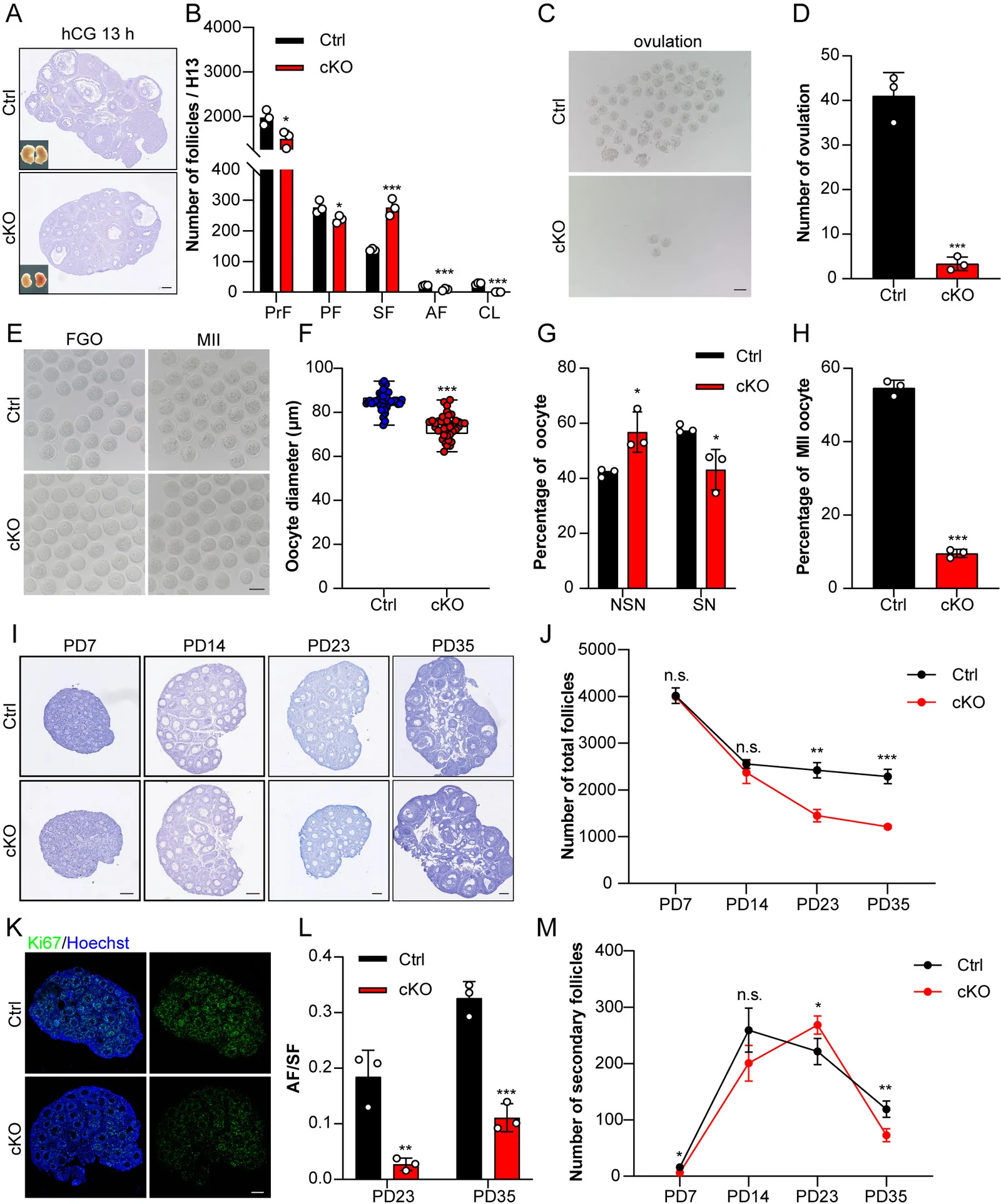
MATR3 was required to support oocyte growth. (**A**) Hematoxylin staining of postnatal day 23 (PD23) ovaries after gonadotropins injection. (**B**) Number of follicles in PD23 ovaries after gonadotropins injection. n=3. (**C**) Morphology and number of oocytes collected from oviducts of PD23 mice after superovulation. n=3. (**D**) Morphology of oocytes derived from PD23 mice primed with pregnant mare serum gonadotropin (PMSG) for 48 hr. n=3. (**E, F**) Diameter of oocytes derived from PD23 mice primed with PMSG for 48 hr. n=40. (**G**) Non-surrounded nucleolus (NSN) and surrounded nucleolus (SN) ratio of oocytes derived from PD23 mice primed with PMSG for 48 hr. n=3. (**H**) MII ratio of oocytes derived from PD23 mice primed with PMSG for 48 hr. n(Ctrl)≥38, n(cKO)≥91. (**I**) Hematoxylin staining of PD7, PD14, PD23, PD35 ovaries. n=3. (**J**) Number of total follicles in PD7, PD14, PD23, PD35 ovaries. n=3. (**K**) Cell proliferation indicated by Ki67-positive GCs in mice ovaries. (**L**) Statistics of antral follicle (AF)/secondary follicle (SF) in PD23 and PD35 ovaries. n=3. (**M**) Number of SFs in PD7, PD14, PD23, PD35 ovaries. n=3. Scale bars: 40 μm in (**C**) and (**E**), 120 μm in (**A**), (**I**), and (**K**). Data are represented as mean ± SD. ***p<0.001, **p<0.01, *p<0.05, n.s., not significant. Figure 2—source data 1.Source data for Figure 2B, D, F–H, J, L and M.

To clarify how MATR3 deficiency hindered mouse fertility, we injected human chorionic gonadotropin (hCG) after intraperitoneal injection of pregnant mare serum gonadotropin (PMSG) for 48 hr to the mouse at postnatal day 23 (PD23), and isolated the ovaries and oocytes after treated for 13 hr (H13). We evaluated the number of follicles at all developmental stages in the ovaries of H13. The results showed that there were no corpora lutea (CL) in the ovaries of cKO mice (Figure 2A and B). Consistently, hCG failed to induce ovulation in cKO mice (Figure 2C and D). Meanwhile, both the number of AFs (Figure 2B) and the ratio of AFs/SFs (Figure 2L) were significantly lower in cKO mice than in the control (Ctrl). GOs from PD14 cKO mice had normal size and morphology (Figure 2—figure supplement 1A and B). While, FGOs from PD23 cKO mice had smaller sizes than Ctrl oocytes (Figure 2E and F) and the ratio of SN-type oocytes in cKO was significantly lower than that in the Ctrl group (Figure 2G). During in vitro maturation, the proportion of oocytes from PD23 cKO mice developing to metaphase II stage was significantly reduced (Figure 2E and H, 54.9±2.08% vs 9.57±1.11%).

**Figure 2—figure supplement 1.**
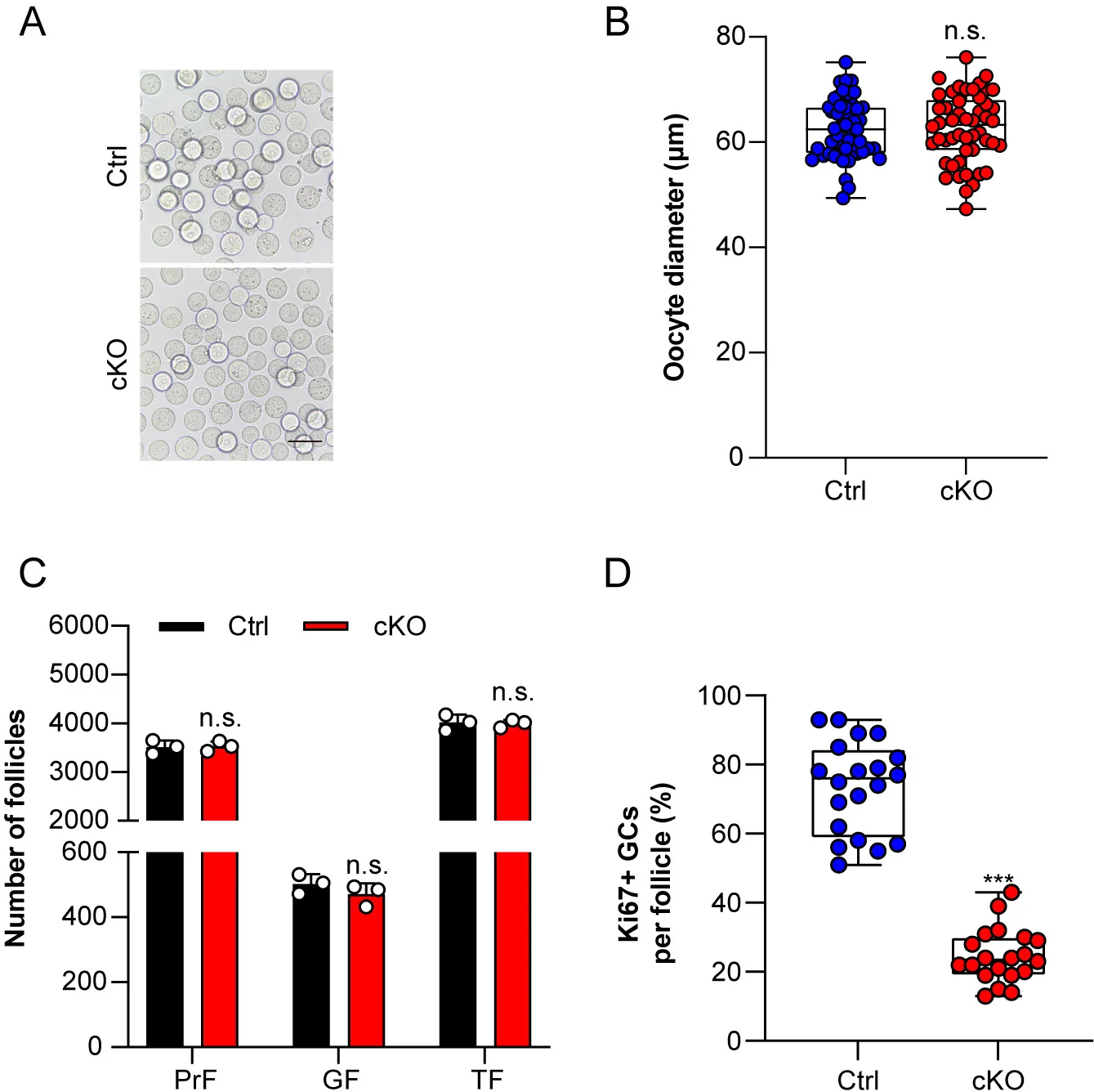
Detection of *Matr3* oocyte-specific knockout mice phenotypes. (**A**) Morphology of oocytes derived from postnatal day 14 (PD14) mice. Scale bar: 50 μm. (**B**) Diameter of oocytes derived from PD14 mice. n=50. (**C**) Number of follicles in PD7 ovaries. PrF: primordial follicle; GF: growing follicle; TF: total follicle. (**D**) Quantification of the ratio of Ki-67-positive granulosa cells within individual follicles. n=20. Data are represented as mean ± SD. ***p<0.001, n.s., not significant. Figure 2—figure supplement 1—source data 1.Source data for Figure 2—figure supplement 1C–E.

**Figure 2—figure supplement 2.**
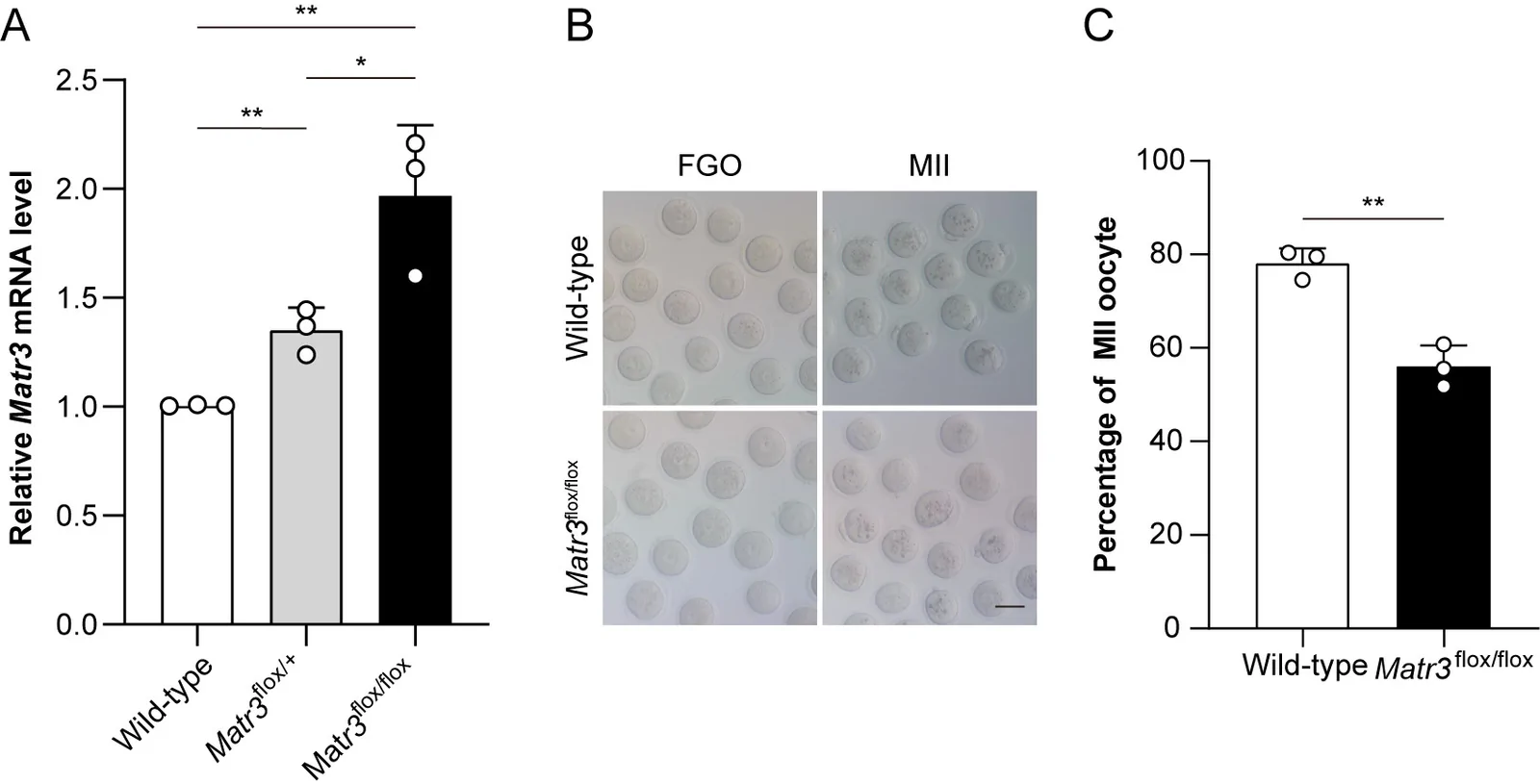
The *Matr3* floxed allele induces a mild hypomorphic effect. (**A**) Relative mRNA expression levels of *Matr3* in mice of different genotypes. n=3. (**B**) Representative images of oocytes derived from postnatal day 23 (PD23) mice primed with pregnant mare serum gonadotropin (PMSG) for 48 hr. Scale bar: 50  μm. (**C**) MII ratio of oocytes derived from PD23 mice primed with PMSG for 48 hr. n (wild-type)>50, n (*Matr3*^flox/flox^)>50. Data are represented as mean ± SD. *p<0.05, **p<0.01. Figure 2—figure supplement 2—source data 1.Source data for Figure 2—figure supplement 2A, C.

To investigate the inner mechanisms of female infertility in cKO mice, we first detected the development of cKO ovaries at different ages (Figure 2I and J). At PD7, a similar morphology of ovaries was found in cKO and Ctrl mice, showing that oocyte deletion of *Matr3* does not affect the formation or survival of dormant primordial follicles (Figure 2—figure supplement 1C). However, the total follicles began to lose at PD14, and a significant difference began to appear at PD23. This was reinforced by the data of available follicles within the ovaries of mice at PD35 (Figure 2I and J). Further statistical results showed that the development of SFs was slowed down from PD7 to PD23, and the conversion rate to AFs was significantly reduced at PD23 (Figure 2M). Consistently, immunofluorescence staining showed that the numbers of Ki67-positive (Figure 2K, Figure 2—figure supplement 1D) in cKO mice were lower than those found in the Ctrl (73.55±13.29% vs 24.65±7.80%).

In summary, the deletion of *Matr3* led to impaired growth of oocytes. Oocyte with impaired growth induced a follicle development disorder in a time-dependent manner attributed to the arrested transition of SFs to AFs.

### MATR3-governed global transcription in GOs is pivotal for follicle growth

To investigate the differences of transcription levels in oocytes between the GOs and FGOs, oocytes were isolated based on their diameters. During the time, 5-ethynyl uridine (EU) was used as probes to measure the levels of newly synthesized mRNA in cells. The results indicated a potential role for MATR3 in the maintenance of high transcriptional levels of GOs, which showed that the expression of MATR3 was consistent with the transcriptional activity of oocytes (Figure 3—figure supplement 1).

**Figure 3.**
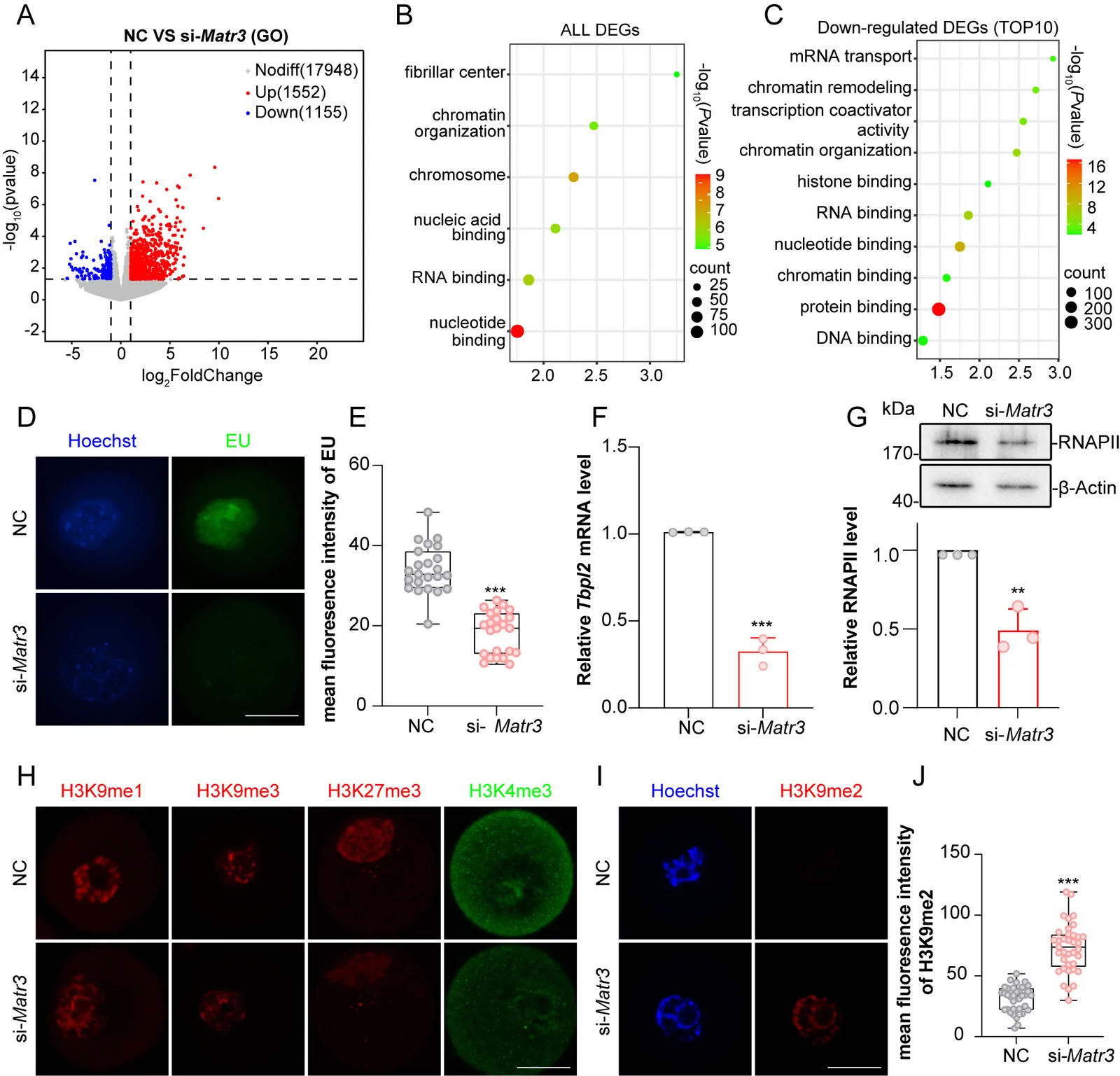
*Matr3* knockdown results in the reduction of transcriptional activity. (**A**) Volcano plot shows differentially expressed genes (DEGs) (upregulated, red; downregulated, blue) in si-*Matr3* oocytes compared to the negative control (NC). (**B**) Key gene ontology (GO) enrichment of all DEGs in GOs. (**C**) Top 10 terms of downregulated DEGs in GOs. (**D**) 5-Ethynyl uridine (EU) staining (green) in GOs collected from NC and si-*Matr3*. n ≥20. (**E**) Quantification of the mean fluorescence intensity of EU in oocytes. (**F**) RT-qPCR results showing *Tbpl2* mRNA level in GOs. (**G**) Western blotting results showing RNAPII protein level in GOs. (**H**) H3K9me1 (red), H3K9me3 (red), H3K27me3 (red), H3K4me3 (green), staining in GOs collected from NC and si-*Matr3*. n ≥20. (**I**) H3K9me2 staining (red) in GOs collected from NC and si-*Matr3*. n ≥37. (**J**) Quantification of the mean fluorescence intensity of H3K9me2 in oocytes. Scale bar: 20 μm. Data are represented as mean ± SD. ***p<0.001, **p<0.01. Figure 3—source data 1.PDF file containing original western blots for Figure 3G, indicating the relevant bands and treatments. Figure 3—source data 2.Original files for western blot analysis displayed in Figure 3G. Figure 3—source data 3.Source data for Figure 3B, C, E–G and J.

To identify the genes regulated by MATR3, the NC and si-*Matr3* oocytes were used for single-cell RNA-sequencing (scRNA-seq). The results of PC clustering analysis and heatmap showed good data parallelism (Figure 3—figure supplement 2). The results showed that 1552 genes were upregulated and 1155 genes downregulated, respectively (|Foldchange|≥2, p-value<0.05, Figure 3A). Gene ontology (GO) enrichment analysis showed that the differentially expressed genes (DEGs) were enriched in the terms related to fibrillar centers, chromatin organization, and nucleotide binding (Figure 3B).

**Figure 3—figure supplement 1.**
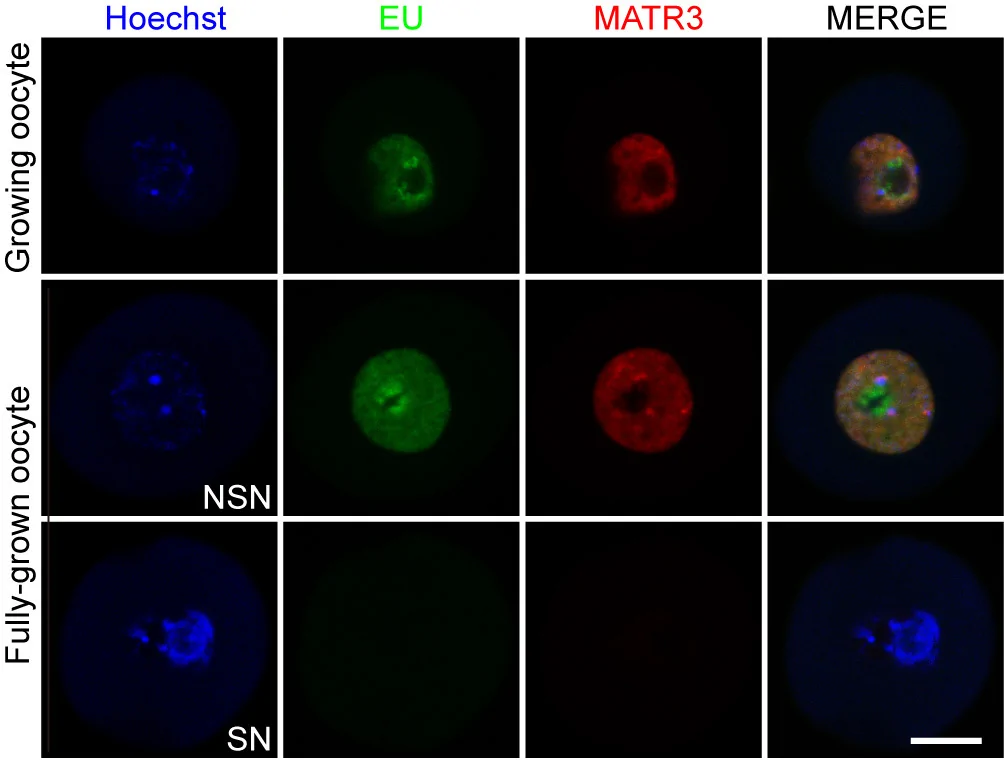
The spatiotemporal-specific localization of MATR3 is adapted to the transcriptional activity of oocytes. Immunofluorescence results showing the relationship between the localization of MATR3 in oocytes at different growth states and the transcriptional activity of oocytes. Red: MATR3; green: 5-ethynyl uridine (EU) (newly synthesized mRNA); blue: Hoechst (cell nucleus). Scale bar: 25 μm.

**Figure 3—figure supplement 2.**
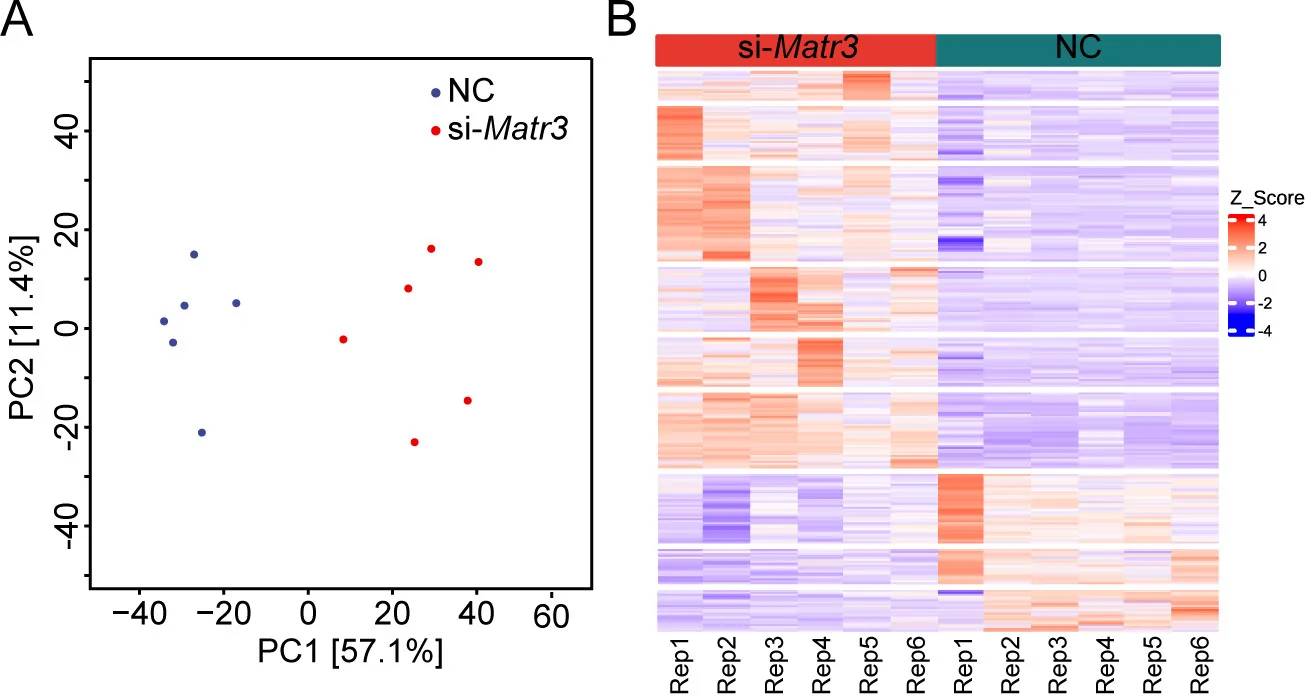
Parallelism analysis of single-cell RNA-sequencing (scRNA-seq) samples. (**A**) PCA shows that the gene ontology (GO) in the negative control (NC) group and the si-*Matr3* group form two subgroups. (**B**) Clustering heatmap analysis demonstrates the global changes in the expression levels of differentially expressed genes in the GOs of the NC group and the si-*Matr3* group based on the results of scRNA-seq data. The horizontal axis represents the types of sample replicates, and the vertical axis represents different genes.

**Figure 3—figure supplement 3.**
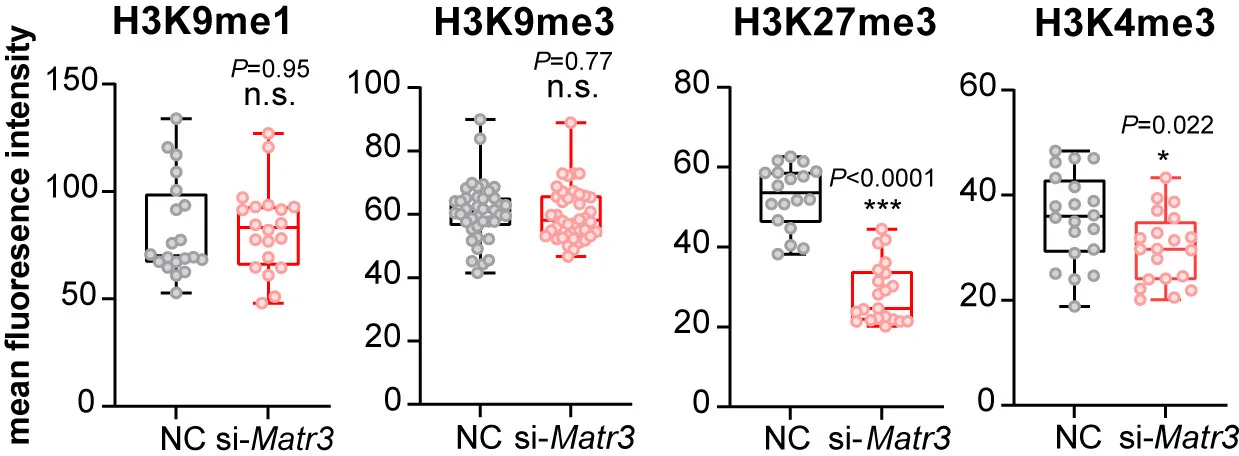
The histone methylation level of oocytes after *Matr3* knockdown in vitro. The statistical results of the fluorescence intensity of histone methylation, with n (H3K9me1)=20, n (H3K9me3)=40, n (H3K27me3)=18, and n (H3K4me3)=20. The statistical results are presented as mean ± SD. Figure 3—figure supplement 3—source data 1.Source data for Figure 3—figure supplement 3.

Subsequently, we performed GO analysis on the downregulated DEGs in oocytes. We found that approximately two-thirds of the top 10 entries were related to transcription (Figure 3C). Based on this, we performed EU staining on oocytes to detect the level of their newly synthesized mRNA. The results showed that, after *Matr3* knockdown, the transcriptional activity of GOs was significantly reduced (Figure 3D and E). The relative mRNA level of the transcription initiation factor *Tbpl2* (Yu et al., 2020), which is specifically expressed in oocytes, was significantly decreased (Figure 3F). Moreover, the relative protein level of RNAPII fell significantly (Figure 3G).

Through GO enrichment analysis of scRNA-seq, it showed that the DEGs were significantly enriched in the ‘chromatin organization’ term, which was highly related to transcription. To prove that MATR3 affects the transcriptional activity of oocytes by regulating the chromatin state, we used immunofluorescence to detect the methylation levels of multiple histones, aiming to find the key molecules responsible for the downregulation of oocyte transcriptional activity. After knocking down *Matr3* in GOs, the protein levels of H3K9me1 and H3K9me3, which were related to gene transcriptional repression, did not change significantly. Contrarily, the protein levels of H3K27me3 and H3K4me3 even decreased significantly (Figure 3H, Figure 3—figure supplement 3). The above results indicate that H3K9me1, H3K9me3, H3K27me3, and H3K4me3 do not play a dominant role in the regulation of GO transcriptional levels mediated by MATR3. Excitingly, the protein level of H3K9me2, which is related to gene transcriptional repression, increased extremely high. The level of H3K9me2, which marks transcriptional repression, was significantly increased either (Figure 3I and J). Based on the above results, MATR3 in GO maintains a high transcriptional level by eliminating the inhibitory modification H3K9me2.

### MATR3 governs follicle growth partially through regulating GDF9 production

During the follicle growth process, a high transcriptional level in GO is a prominent physiological feature. The substantial accumulation of maternal mRNAs, proteins, and metabolites in the cytoplasm of GO is a crucial factor contributing to the gradual increase in oocyte volume. It is also essential for the development of early-growing follicles. Among these, GDF9 plays a vital role in regulating the development of early-growing follicles. Specifically, we examined the key factors related to the GDF9 signaling pathway to verify the paracrine signals.

**Figure 4.**
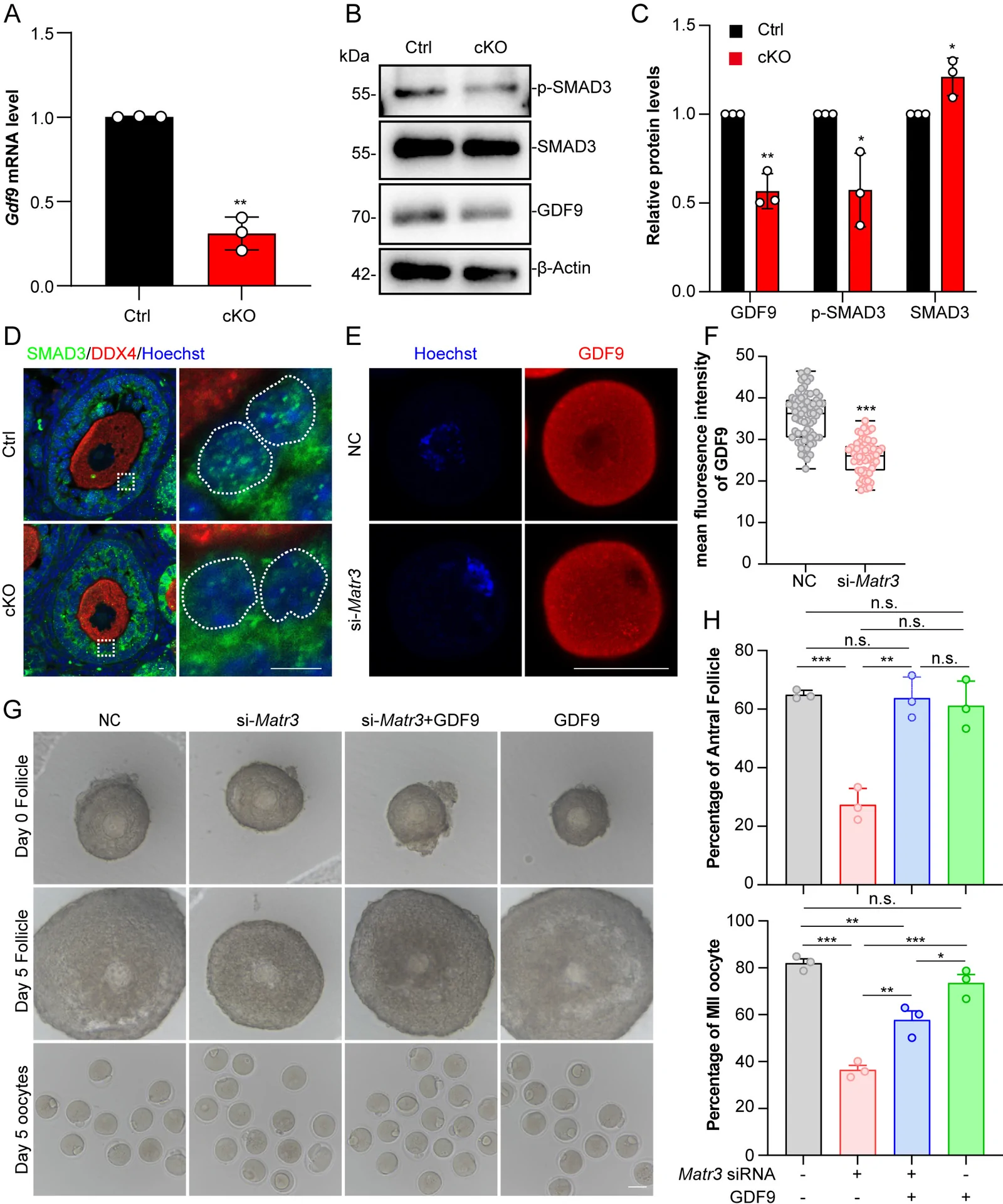
MATR3 controls oocyte growth by regulating GDF9 level. (**A**) RT-qPCR results showing *Gdf9* mRNA level in postnatal day 14 (PD14) ovaries. (**B, C**) Western blotting results showing GDF9, SMAD3, and p-SMAD3 protein levels in PD14 ovaries. (**D**) p-SMAD3 staining (green) in PD14 ovaries. (**E**) GDF9 staining (red) in gene ontologies (GOs) from negative control (NC) and si-*Matr3*. n ≥68. (**F**) Quantification of the mean fluorescence intensity of GDF9 in oocytes. (**G**) Representative image of follicles and oocytes from NC, si-*Matr3*, si-*Matr3*+GDF9, GDF9. (**H**) Percentage of antral follicles and MII oocytes in (**G**). n≥3 per group. Scale bar: 40 μm in (**D**) and (**E**), 80 μm in (**G**). Data are represented as mean ± SD. ***p < 0.001, **p < 0.01, *p < 0.05, n.s., not significant. Figure 4—source data 1.PDF file containing original western blots for Figure 4B, indicating the relevant bands and treatments. Figure 4—source data 2.Original files for western blot analysis displayed in Figure 4B. Figure 4—source data 3.Source data for Figure 4A, C, F and H.

The results showed that the *Gdf9* level was decreased significantly in cKO ovaries, which was confirmed by the mRNA and protein expression assays (Figure 4A–C). When *Matr3* in oocytes was knocked down in vitro, the level of GDF9 was significantly decreased consistently (Figure 4E and F). Generally, the GDF9 signal is transmitted to SMAD3 in the cytoplasm of granulosa cells and enters the nucleus through phosphorylation, thereby promoting the differentiation of granulosa cells. Compared with the Ctrl group, the level of phosphorylated SMAD3 protein was decreased in cKO ovaries (Figure 4B). Although there was no significant difference in SMAD3 protein levels, most of the SMAD3 molecules could not be phosphorylated and were therefore located in the cytoplasm rather than inside the nucleus of the granulosa cells (Figure 4D).

To prove that GDF9 is a key downstream molecule whose production is affected by MATR3, we conducted a rescue experiment on growing follicles with *Matr3*-knocked-down oocytes by adding pure GDF9 in vitro. We divided the in vitro-cultured follicles into four groups: the NC group, the *Matr3*-knocked-down group, the group with GDF9 added after *Matr3* knockdown, and the group with GDF9 added alone. Consequently, after knocking down *Matr3* in the oocytes of SFs and culturing them in vitro for 5 days, we photographed and recorded the follicle diameters, and counted the extrusion rate of the first polar body of oocytes (Figure 4G). The results showed that the follicle diameter of the group with GDF9 added after *Matr3* knockdown significantly rescued that of the group with *Matr3* knocked down alone. And, there was no significant difference compared with the control group. The extrusion rate of the first polar body in the group with GDF9 added after *Matr3* knockdown also significantly rescued that of the group with *Matr3* knocked down alone, but there was still a significant difference compared with the extrusion rate of the first polar body in the NC group (Figure 4H). These results indicate that GDF9 can partially rescue the follicular development arrest caused by *Matr3* knockdown and is a key functional molecule in MATR3-regulated follicular development.

### MATR3 promotes the expression of *Gdf9* by recruiting KDM3B

**Figure 5.**
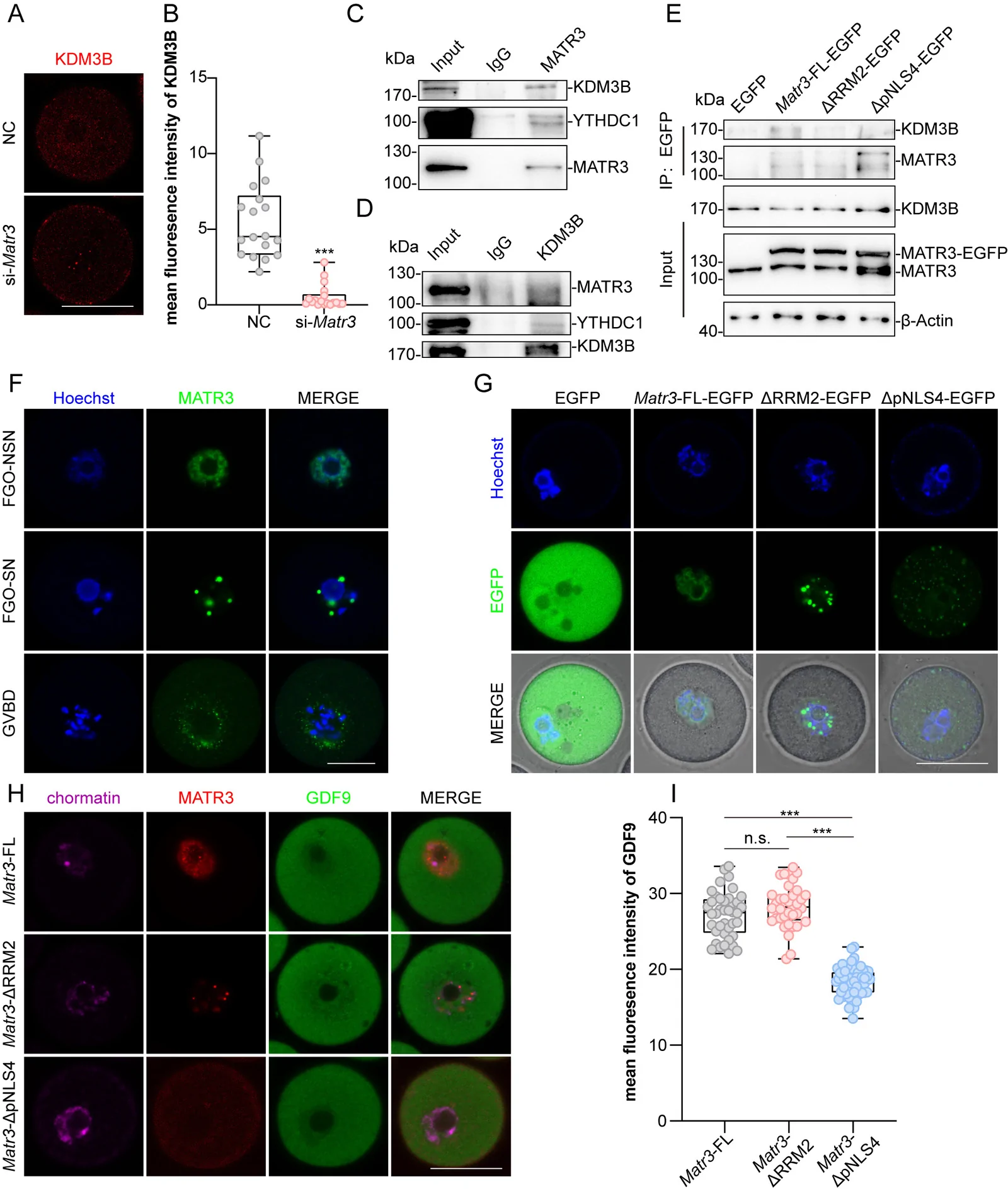
MATR3 promoted GDF9 by recruiting KDM3B. (**A**) KDM3B staining (red) in gene ontology (GO) collected from negative control (NC) and si-*Matr3*. n ≥18. (**B**) Quantification of the mean fluorescence intensity of KDM3B (**A**) in oocytes. (**C, D**) Co-immunoprecipitation (co-IP) showing protein interactions between MATR3 and KDM3B in HEK293T cells. (**E**) Co-IP results after HEK293T cells were treated with pCDNA3.1-EGFP, pCDNA3.1-*Matr3*-EGFP (*Matr3*-FL-EGFP), pCDNA3.1-*Matr3*-ΔRRM2-EGFP (ΔRRM2-EGFP), or pCDNA3.1-*Matr3*-ΔpNLS4-EGFP (ΔpNLS4-EGFP) plasmid. (**F**) Live-cell imaging of oocytes after injecting *Matr3-*EGFP mRNA for 12 hr. (**G**) Live-cell imaging of oocytes after injecting EGFP, *Matr3*-FL-EGFP, ΔRRM2-EGFP, or ΔpNLS4-EGFP mRNA for 12 hr. (**H**) MATR3 (red) and GDF9 (green) staining in GO which had knocked down MATR3 protein before injected *Matr3*-FL, *Matr3*-ΔRRM2, or *Matr3*-ΔpNLS4 mRNA. n ≥36. (**I**) Quantification of the mean fluorescence intensity of GDF9 (**H**) in oocytes. Scale bar: 40 μm. Data are represented as mean ± SD. ***p<0.001, n.s., not significant. Figure 5—source data 1.PDF file containing original western blots for Figure 5C, indicating the relevant bands and treatments. Figure 5—source data 2.Original files for western blot analysis displayed in Figure 5C. Figure 5—source data 3.PDF file containing original western blots for Figure 5D, indicating the relevant bands and treatments. Figure 5—source data 4.Original files for western blot analysis displayed in Figure 5D. Figure 5—source data 5.Source data for Figure 5B, I.

To explore how MATR3 is involved in the regulatory synthesis of GDF9, we detected the level of lysine (K)-specific demethylase 3B (KDM3B), which directly regulates H3K9me2. Under physiological conditions, KDM3B is localized in the cytoplasm and nucleus of oocytes, and is highly expressed in GO (Figure 5—figure supplement 1). This study showed that KDM3B was downregulated as the transcriptional activity of oocytes decreased (Figure 5A and B). To further clarify how MATR3 regulates KDM3B, previous literature indicates that RBPs can recruit KDM3B to regulate the level of H3K9me2 (Kim et al., 2012; Li et al., 2020). Then, to prove this hypothesis, we detected the interaction between MATR3 and KDM3B in the HEK293T cell line using co-immunoprecipitation (co-IP). The results showed that there was an interaction between MATR3 and KDM3B (Figure 5C and D). To identify the specific domains of MATR3 for recruiting KDM3B, we first constructed the MATR3-EGFP fusion protein. The western blotting results demonstrated the successful construction of the vector after we transfected the HEK293T cell line with the constructed plasmids (Figure 5—figure supplement 2A and B).

**Figure 5—figure supplement 1.**
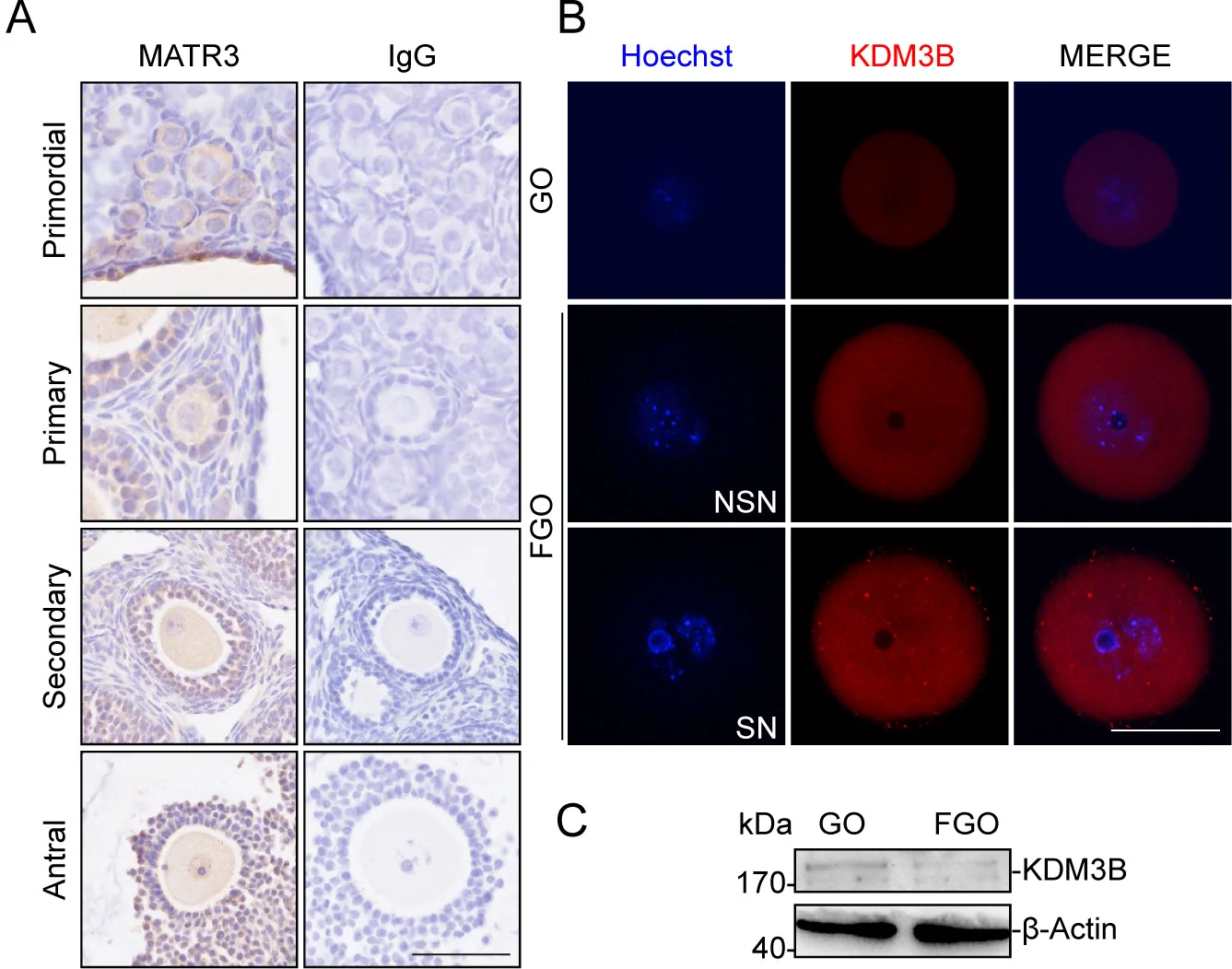
The expression pattern of KDM3B in the ovaries of mice. (**A**) Immunohistochemistry results showing KDM3B location in mice ovaries. (**B**) Immunofluorescence results showing the location of KDM3B in gene ontology (GO) and fully grown oocyte (FGO). (**C**) Western blotting results showing KDM3B protein levels in GO and FGO. Scale bar: 50 μm. Figure 5—figure supplement 1—source data 1.PDF file containing original western blots for Figure 5—figure supplement 1C, indicating the relevant bands and treatments. Figure 5—figure supplement 1—source data 2.Original files for western blot analysis displayed in Figure 5—figure supplement 1C.

**Figure 5—figure supplement 2.**
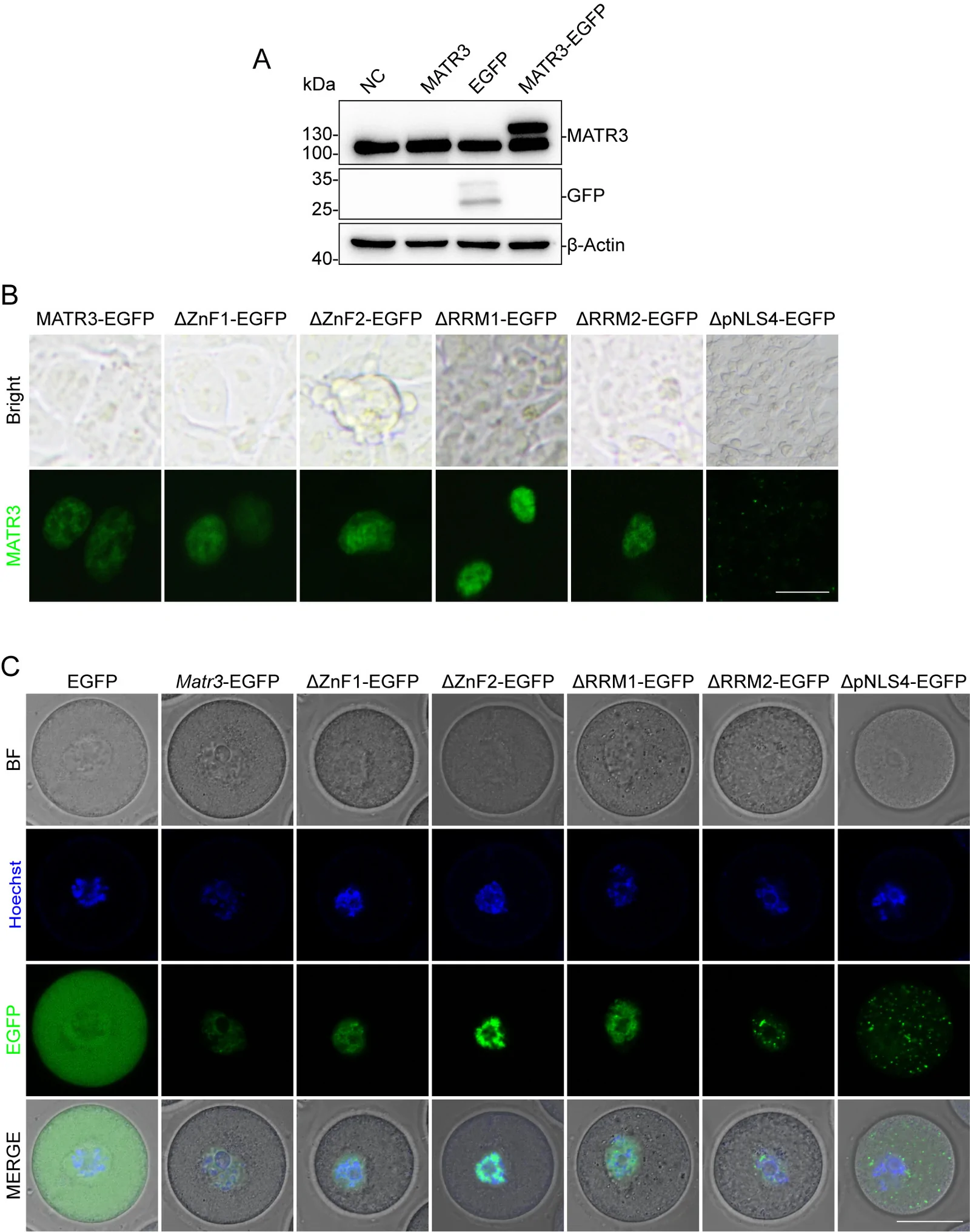
Construction and localization detection of MATR3 truncated forms. (**A**) Western blotting was used to detect the protein expression levels of the 293 cell line after being transfected with negative control (NC), MATR3, EGFP, and MATR3-EGFP plasmids for 48 hr, respectively. β-Actin is used as an internal reference to calibrate the protein loading level. (**B**) Immunofluorescence was used to detect the protein localization of the 293 cell line after transfection with MATR3-EGFP, ΔZnF1-EGFP, ΔZnF2-EGFP, ΔRRM1-EGFP, ΔRRM2-EGFP, and ΔpNL4-EGFP plasmids for 48 hr. (**C**) Immunofluorescence was used to detect the effect of the functional domains of MATR3 on its localization in living gene ontology (GO). Green: EGFP, MATR3 forming a fusion protein with EGFP; blue: Hoechst (nucleus). Scale bar: 10 μm in (**B**), 25 μm in (**C**). Figure 5—figure supplement 2—source data 1.PDF file containing original western blots for Figure 5—figure supplement 2A, indicating the relevant bands and treatments. Figure 5—figure supplement 2—source data 2.Original files for western blot analysis displayed in Figure 5—figure supplement 2A.

We injected low-concentration of *Matr3*-EGFP mRNA into either GOs or FGOs and performed live-cell fluorescence imaging. The results showed that in GOs and NSN-type FGOs, MATR3 was localized in the nucleus, while in SN-type oocytes, MATR3 presented as aggregated punctate structures and diffused into the cytoplasm as meiosis resumes (Figure 5F). In addition, we injected the truncated form into germinal vesicle (GV) oocytes to further detect the localization of MATR3 (Figure 5—figure supplement 2C). The co-IP results indicated that MATR3 interacts with KDM3B through the second RNA recognition motif (RRM2) and the fourth nuclear localization signal (pNLS4) (Figure 5E). Furthermore, we injected in vitro-transcribed mRNAs (from EGFP, *Matr3*-EGFP, *Matr3*-ΔRRM2-EGFP, *Matr3*-ΔpNLS4-EGFP plasmids) into GOs and carried out living-cell fluorescence imaging. The results showed that the functional truncation mutants of the RRM2 motif and the pNLS4 motif both change the spatiotemporal-specific localization of MATR3 (Figure 5G).

To determine the domain that affects the physiological function of MATR3 in oocytes, we first knocked down the endogenous level of MATR3 in GOs and then injected *Matr3*-EGFP, *Matr3*-ΔRRM2-EGFP, or *Matr3*-ΔpNLS4-EGFP mRNAs, respectively, so as to detect the protein level of GDF9. The results showed that after deleting RRM2, there was no significant change in the GDF9 protein level, while after deleting the pNLS4 domain, the GDF9 level decreased significantly (Figure 5H and I). Together, these results suggested that MATR3 is localized in the nucleus of GOs through the pNLS4 domain, thereby recruiting KDM3B and promoting the expression of GDF9.

### MATR3 promotes the expression of *Rdx* to facilitate GDF9 secretion

**Figure 6.**
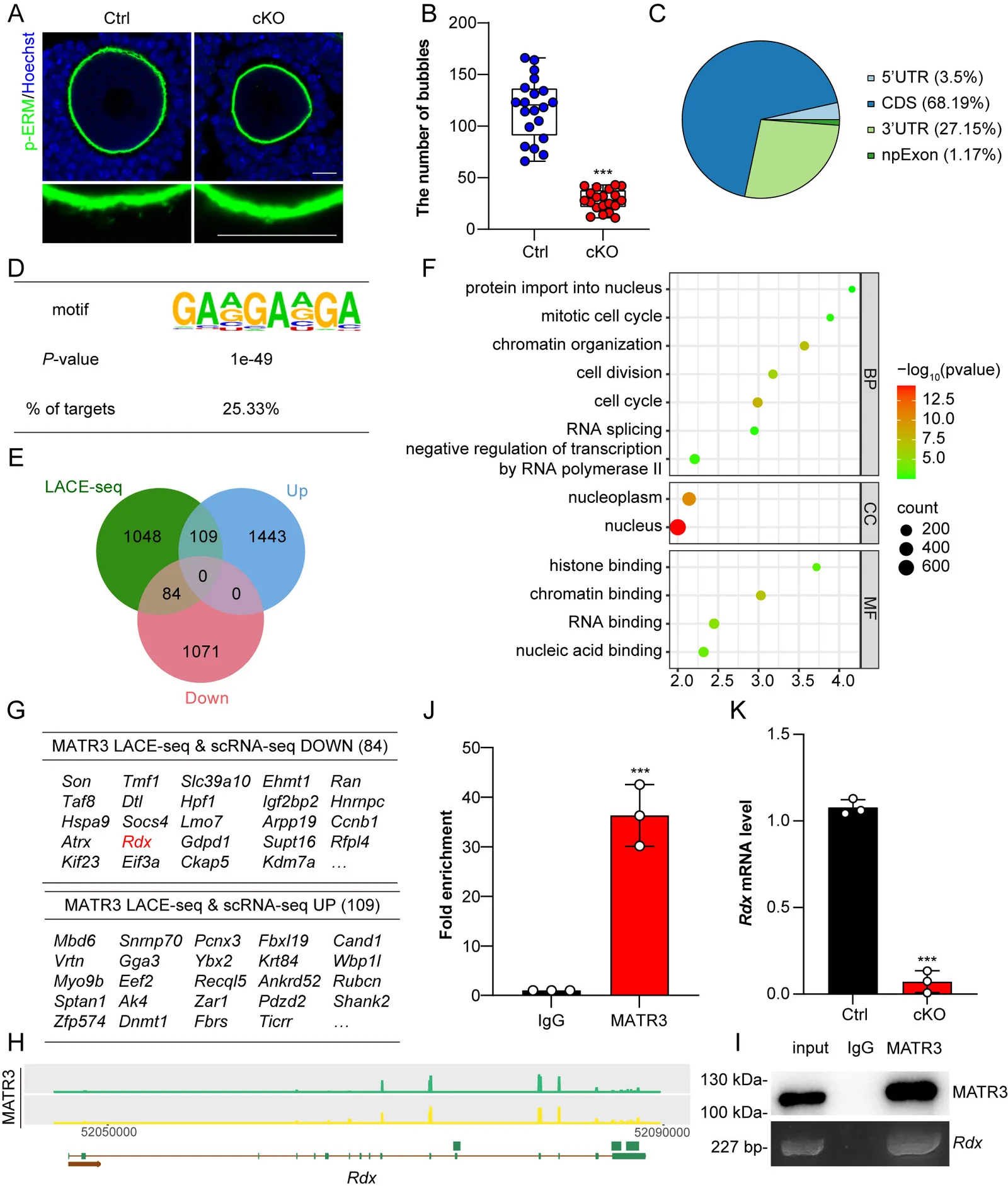
MATR3 is required for the oocyte-derived mushroom-like microvilli (OO-Mvi) in mouse oocyte by regulating *Rdx* mRNA level. (**A**) p-ERM staining (green) showing the OO-Mvi in antral follicles (AFs) from Ctrl and cKO. (**B**) Quantification of the number of Oo-Mvi vesicles (n = 20). (**C**) Pie chart showing the proportion of the reads values of 10- to 12-day-old mice MATR3 low-input affinity cleavage enrichment sequencing (LACE-seq). (**D**) Table showing the base sequences to which MATR3 mainly binds. (**E, G**) Venn diagrams (**E**) and table (**G**) showing overlapping genes binding with MATR3, upregulated genes, and downregulated genes in single-cell RNA-sequencing (scRNA-seq). (**F**) Key gene ontology (GO) enrichment of all differentially expressed genes (DEGs) in LACE-seq. (**H**) IGV snapshot of MATR3 peaks distribution in 10- to 12-day-old mice ovaries. (**I, G**) RNA immunoprecipitation (RIP) showing the interaction between MATR3 and *Rdx* mRNA in 10- to 12-day-old mice ovaries. Western blotting showing the reliability of MATR3 antibody (**I**). Enrichment degrees of MATR3 on the *Rdx* mRNA, respectively (**J**). IgG served as the negative control and input (2%) served as the positive control. n=3. (**K**) RT-qPCR results showing *Rdx* mRNA level in postnatal day 14 (PD14) oocytes. Scale bar: 20 μm. Data are represented as mean ± SD. ***p<0.001. Figure 6—source data 1.PDF file containing original western blots and gels for Figure 6I, indicating the relevant bands and treatments. Figure 6—source data 2.Original files for western blot and gel analysis displayed in Figure 6I. Figure 6—source data 3.Source data for Figure 6 B, F, J and K.

As a key OSF of oocyte paracrine, both the transcription and secretion of GDF9 are regulated by MATR3. Notably, our immunofluorescence results showed that the structure of Oo-Mvi in cKO mice was damaged (Figure 6A and B), which may impair the communication of oocyte and the surrounding cumulus cells.

**Figure 6—figure supplement 1.**
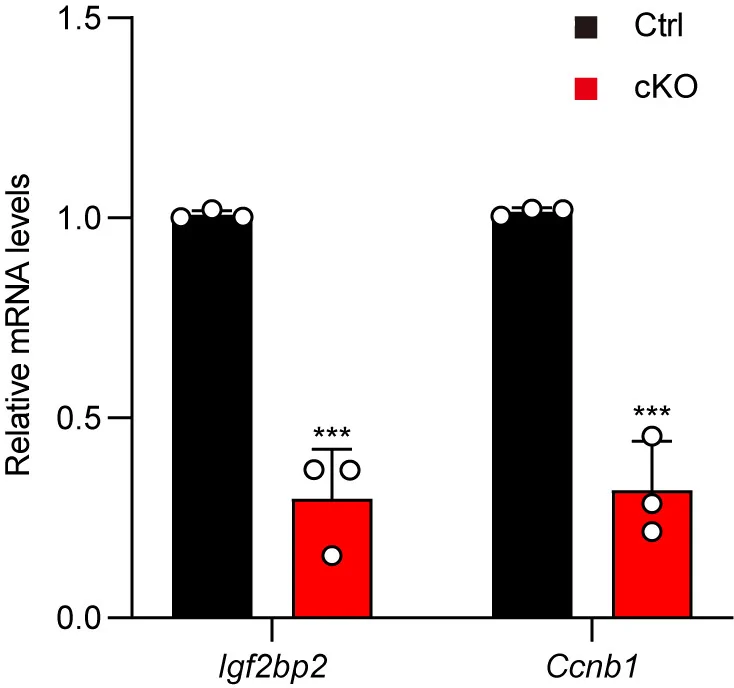
Targets with high binding intensity and significant expression changes from the low-input affinity cleavage enrichment sequencing (LACE-seq) data. RT-qPCR results showing *Igf2bp2* and *Ccnb1* mRNA level in postnatal day 14 (PD14) oocytes. Data are represented as mean ± SD. ***p<0.001. Figure 6—figure supplement 1—source data 1.Source data for Figure 6—figure supplement 1.

To test if MATR3 in GO affects oocyte-somatic cell communication by binding to the DNA sequences pivotal for oocyte growth, the low-input affinity cleavage enrichment sequencing (LACE-seq) assay was performed by collecting GOs from 10- to 12-day-old mice. The sequencing results showed that MATR3 directly binds to the coding sequence (CDS) region and 3' untranslated region of mRNAs (Figure 6C). Motif analysis indicated that MATR3 tends to bind to regions rich in GA (Figure 6D). The results of GO enrichment analysis showed that the genes bound by MATR3 were significantly enriched in biological processes such as nucleocytoplasmic protein shuttling, chromatin assembly, and RNA splicing (Figure 6F).

Later, a joint analysis of LACE-seq and scRNA-seq was performed to further confirm our assumption that MATR3 is very important for oocyte-cumulus cell communication. The results showed that among the genes directly bound by MATR3, 84 genes such as *Son*, *Ehmt1*, *Ran*, *Igf2bp2*, *Ccnb1* (Figure 6—figure supplement 1), and *Rdx* simultaneously showed a significant decrease while 109 genes simultaneously showed a significant increase in the oocytes of the knockdown group (Figure 6E and G). Of which, RDX is a specific Oo-Mvi-related protein in oocytes that contributes to the formation of Oo-Mvi in oocyte development. The sequencing data were visualized by IGV. The results showed that MATR3 directly binds to *Rdx* mRNA (Figure 6H). Fifty of the ovaries of 10- to 12-day-old mice were collected for RNA immunoprecipitation (RIP). Afterward, RIP was used to verify the direct binding of MATR3 to *Rdx* mRNA in the ovaries. Consistent with the sequencing results, MATR3 directly binds to *Rdx* mRNA (Figure 6I and J). Furthermore, the *Rdx* mRNA level in the GOs of the cKO group was significantly decreased (Figure 6K), respectively, as compared with the mice in the Ctrl group. In sum, the above results indicate that in GOs, MATR3 directly binds to *Rdx* mRNA and regulates its level, thereby participating in the GDF9 secretion through Oo-Mvi.

## Discussion

**Figure 7.**
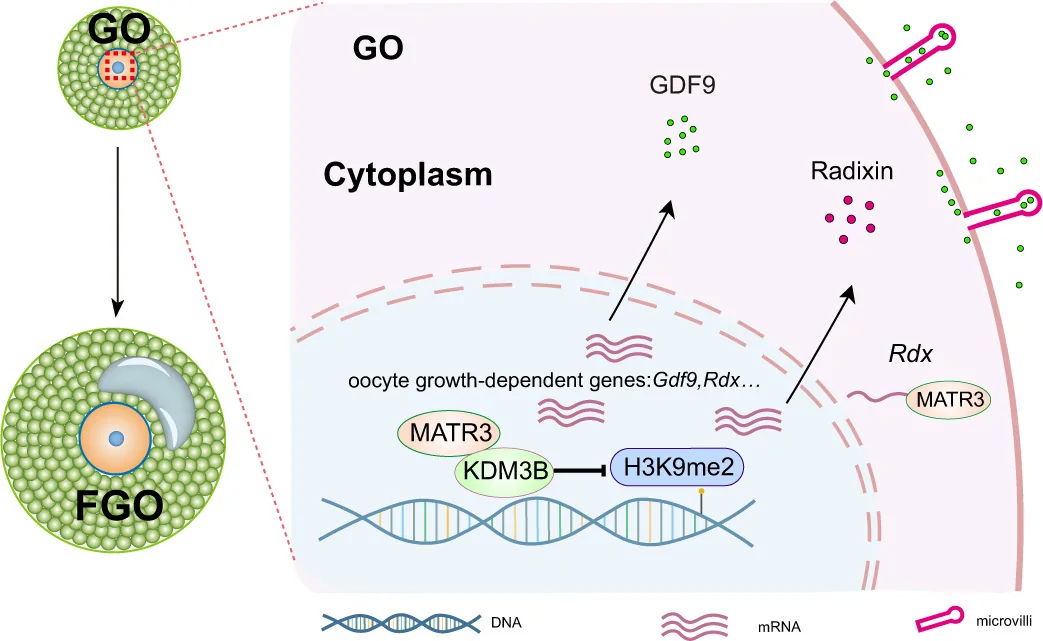
A proposed model: MATR3 regulates the synthesis and secretion of oocyte-secreted factors (OSFs), such as GDF9, thereby ensuring growing oocyte (GO) growth. Under physiological conditions, MATR3 in the GOs promotes the transcription of a large number of genes required for cell growth. When promoting the transcription of the *Gdf9* gene, MATR3 localizes in the nucleus through the pNLS4 domain to recruit KDM3B, thereby promoting the high-level expression of *Gdf9*. In ensuring the secretion of GDF9, MATR3 directly binds to *Rdx* mRNA to promote the growth of microvilli, which in turn lays the foundation for ensuring the secretion of factors, such as GDF9, and establishing the physical connection between oocytes and somatic cells.

This study shows that the spatiotemporal-specific localization of MATR3 in GO is required for female fertility. MATR3 in GO not only affects the transcription of specific genes but influences the overall chromatin organizational conformation. Further, in the condition that Matr3 is deleted in vivo or is silenced in vitro, few growing follicles could develop into AFs. Consequently, the efficiency of superovulation is markedly downregulated, indicating a poor response to gonadotropins in cKO mice. When FGOs were cultured in vitro, the extrusion rate of the first polar body was similarly decreased. The first polar body extrusion rate of the control group remained at approximately 55%, which may be attributed to a mild hypomorphic effect induced by the *Matr3* floxed allele (Figure 2—figure supplement 2). In vitro, GDF9 partially rescued the phenotype of AF failure, as well as oocyte maturation, caused by si-*Matr3*, implying that GDF9 is one of the downstream molecules of MATR3. Furthermore, reduced GDF9 and RDX productions induced by MATR3 missing impairs the mutual communication between GO and surrounding granulosa cells. In agreement with the existing reports, this is one of the typical examples to emphasize the importance of OSFs on granulosa cell proliferation, as well as GO growth (Gilchrist et al., 2008). We further demonstrated that MATR3 may exert its action either by coordinating KDM3B-directed histone methylation and chromosome structure loosening or by improving gene transcription through directly binding to the promoters of targeted genes. Since the protein expression patterns of MATR3 in oocytes of humans, porcine, and mice are similar to each other, MATR3 is a conservative key regulator of GO growth and follicle development among mammals (Figure 7). Therefore, MATR3 is a key regulator of oocyte growth and follicular development.

During follicle growth, the high transcriptional activity in GO is a prominent physiological feature of oocytes. The uniqueness of GOs lies in their high transcriptional activity when compared with oocytes at other developmental stages. When primordial follicles are activated, oocytes in the follicles are transformed from a dormant state to the growing state. At this time, a large number of mRNAs start to be transcribed within the oocytes. As GOs progress to FGOs, transcriptional silencing occurs, which persists until the two-cell stage. This indicates that the synthesis and accumulation of mRNAs for subsequent oocyte maturation, fertilization, and early embryo development mainly occur in the GO. Consequently, the large amounts of maternal mRNAs, proteins, and metabolites accumulated in the cytoplasm of FGOs are important reasons for the gradual increase in oocyte volume and are also essential for the oocytes to acquire the abilities of meiosis, fertilization, and embryo development (Su et al., 2021). Therefore, the high transcriptional level of mRNAs during the growth process of GOs is of particular importance.

MATR3 could also be one of the candidate causative RBPs contributing to OMA in females. Recently, mutations of some RBPs pivotal for maternal mRNA homeostasis have been linked to the consequence of OMA, including PATL2 (Hu et al., 2024), PABPC1L (Wang et al., 2023), and ZFP36L2 (Wan et al., 2024). Here, the localization of MATR3 changes in oocytes of OMA patients as well. Specifically, MATR3 is highly expressed in the nucleus of NSN oocytes and exits the nucleus when the nuclear type transforms to SN in healthy people. However, in an OMA oocyte that remained at the GV stage after ICSI, the nuclear localization of MATR3 disappeared. Moreover, the diameter of this oocyte was smaller than that of normal FGOs. Furthermore, we noticed that deletion of MATR3 in mouse GOs resulted in significant chromosome configuration disorders, as the percentage of SN vs NSN FGOs was reversed in cKO mice. These findings imply that MATR3 is pivotal for chromosome configuration. In agreement with existing studies explaining that the SN chromatin configuration correlates with transcriptional silence and full oocyte developmental competence (Bouniol-Baly et al., 1999; Zuccotti et al., 2005), this study also found that multiple oocyte-specific genes and genes involved in oocyte/granulosa cell communication are direct targets of MATR3, including *Ehmt1* (Demond et al., 2023), *Ran* (Dehapiot and Halet, 2013), *Igf2bp2* (Li et al., 2021), *Ccnb1* (Wang et al., 2024a), *Rdx* (Zhang et al., 2021), *Zar1* (Rong et al., 2019), and *Dnmt1* (Shelby et al., 2023). The transcription of these genes changed in the condition of MATR3 loss. Therefore, MATR3 is pivotal for chromosome configuration and global gene transcription in GOs, which is meaningful for the growth of oocytes. Despite these findings, however, it is too early to treat MATR3 as one of the biomarkers for OMA since we could not collect more oocytes from OMA to confirm the conclusion so far. More clinical studies are needed to give a confirmed relationship between MATR3 and OMA. Possibly, analysis of any mutations or irregular expression levels of MATR3 in immature oocytes of OMA patients may be pivotal to confirm the causal relationship.

This study provided additional evidence to highlight the crucial roles of OSFs in supporting follicular development at different stages (Dong et al., 1996). Studies including ours have shown that GDF9 is indispensable for granulosa cell proliferation during the transition from primary follicles to SFs (Dong et al., 1996; Ackert et al., 2001; Gao et al., 2024). In the differentiated cumulus cells of AFs, GDF9 inhibits the synthesis of estrogen and the expression of LH receptors, thereby maintaining the differentiated state of cumulus cells. Meanwhile, it promotes the expression of NPR2 in cumulus cells, upregulates the levels of cGMP and cAMP in oocytes, and contributes to the oocyte meiotic arrest (Hinckley et al., 2005; Norris et al., 2009; Zhang et al., 2010). In vitro, GDF9 promotes the synthesis of new transzonal projections in the cumulus-oocyte complex (COC) (El-Hayek et al., 2018). Besides the powerful roles in regulating multiple gene expressions responsible for oocyte development and oocyte/granulosa cell mutual communication, this study specifically emphasized the importance of MATR3-controlled production and secretion of GDF9 on AF formation, as well as oocyte maturation, with the assistance of RDX for establishing profound Oo-Mvi. This is one of the typical examples to emphasize the importance of OSFs on granulosa cell proliferation, GO growth, as well as AF formation, which are all pivotal for supporting high-quality oocyte development. This study highlights the importance of a sustaining feedback regulation between the oocyte and the surrounding somatic cells on GO growth and follicle development.

Previous studies reported that GOs could be divided into three stages based on the diameter, namely GO1 (30–45 μm), GO2 (50–55 μm), and GO3 (60–65 μm) (Gu et al., 2019). According to our results, there is no significant difference in the abundance of transcripts and chromatin accessibility among the three states of GO, but the DNA methylation levels vary significantly. After knocking down *Matr3* in GO2, the levels of H3K4me3 and H3K27me3 decreased significantly, which is contrary to the increasing trend of H3K4me3 and H3K27me3 levels with oocyte growth depicted in the DNA methylation map. The results prove that MATR3 is a positive regulator on GO growth. During the GO growth, the levels of classical histone methylation modifications H3K4me3 and H3K9me3 increase and reach their peaks in FGO (Kageyama et al., 2007). Here, the levels of H3K9me1 and H3K9me3 linked to gene transcriptional repression were unchanged while H3K27me3 and H3K4me3 decreased significantly, indicating that classical histone methylations do not play dominant roles in MATR3-mediated transcription in GO. Instead, the level of H3K9me2, which is related to gene transcriptional repression, increased significantly. Consistently, the level of KDM3B, which directly regulates H3K9me2, decreased significantly (Kim et al., 2012). So, H3K9me2 is crucial for MATR3-mediated high transcriptional levels in GO. Specifically, the pNLS4 domain of MATR3 recruits KDM3B and regulates the transcriptional synthesis of GDF9 by removing the inhibitory H3K9me2 mark. Besides its cooperation with KDM3B to regulate gene transcription ability, MATR3 may directly bind to the promoters of target genes, such as *Rdx*, to regulate transcription.

Overall, this study identifies MATR3 as a pivotal RBP essential for oocyte growth and female fertility, with conserved roles across multiple species, including human, porcine, and mouse. We demonstrate that MATR3 operates through a dual molecular mechanism: it epigenetically promotes the transcriptional synthesis of *Gdf9* by recruiting the H3K9me2 demethylase KDM3B and post-transcriptionally facilitates GDF9 secretion via binding and stabilizing *Rdx* mRNA. Disruption of this coordinated regulatory network impairs granulosa cell communication and arrests follicular development at the secondary stage, ultimately leading to infertility. Given its functional conservation and central role in oocyte maturation, MATR3 represents a promising diagnostic marker and therapeutic target for clinical OMA.

## Materials and methods

### Animals

*Gdf9*-Cre mice were generated by Prof. Fengchao Wang (Transgenic Animal Center, National Institute of Biological Sciences, Beijing, China). *Matr3* ^flox/flox^ mice were generated by Prof. Fengchao Wang using CRISPR-Cas9 technology. The sgRNAs were prepared using MEGAshortscript T7 Transcription Kit (Ambion) according to the manufacturer’s instructions. Cas9 protein, sgRNAs, and donor templates were injected into C57BL/6J fertilized eggs. Injected zygotes were transferred into pseudo-pregnant CD1 female mice. The sequences of gRNA and primers used for genotyping were listed in Supplementary file 1.

### Mouse fertility and ovulation assay

For the mice fertility test, a 2-month-old cKO female and its littermate control (Ctrl) female were housed with a 2-month-old C57BL/6J male with normal fertility. Each male mouse was housed with one cKO and one Ctrl female mouse simultaneously. Mating cages were monitored daily. The number of pups (both alive and dead) was counted on the first day of delivery. The mating process lasted 6 months.

For superovulation, cKO and Ctrl mice at post parturition 23 days were intraperitoneally injected with 5 IU PMSG (Ningbo Sansheng Biological Technology, Cat#110251283), followed by 5 IU hCG (Ningbo Sansheng Biological Technology, Cat#110041282) 46 hr later. After an additional 13 hr, oocytes of each mouse were collected from oviducts, and the number of oocytes was counted after digesting with 0.3% hyaluronidase (Merck, Cat#MR-051-F).

### Follicle counting

Fresh ovarian samples were fixed in 4% paraformaldehyde (Santa Cruz, Cat#30525-89-4) overnight, embedded in paraplast (Leica, Cat#39601095), and sectioned serially at 8 μm. Tissue sections were stained with hematoxylin (Solarbio, Cat#G4070) to count the number of follicles. Sections were examined and photographed using VENTANA DP200 (Roche).

We classified the follicle stage by its morphological characteristics. A PrF contained an oocyte with a diameter less than 20 μm and one layer of flattened pre-granulosa cells (GCs); a PF contained a larger oocyte and one layer of cubical GCs; an SF had multilayer GCs; an AF had antral cavity.

The number of every follicle stage was summarized by counting all sequential sections. In both cases, only follicles containing clearly visible oocyte nuclei in each individual section were counted to avoid repetitive counting.

### Immunostaining and biological assays

The ovarian sections were deparaffinized, rehydrated, and subjected to high-temperature (95–98°C) antigen retrieval for 16 min with 0.01% sodium citrate buffer (pH 6.0). Immunohistochemistry assay was performed using Histostain-SP Kits (ZSGB-BIO, Cat#PV-9001) and DAB peroxidase substrate kits (ZSGB-BIO, Cat#ZLI-9017) according to the manufacturer’s protocols. Nuclei were stained with hematoxylin (Solarbio, Cat#G4070). Primary antibodies and dilution rates were as follows: rabbit anti-MATR3 (1:400, Abcam, Cat#ab151714). Sections were examined and photographed using VENTANA DP200 (Roche).

For immunofluorescence assay, ovarian paraffin sections were deparaffinized, rehydrated, and subjected to high-pressure antigen repair with 0.01% sodium citrate buffer (pH = 6.0) for 16 min. The sections were then rinsed thoroughly with phosphate-buffered saline (PBS) for 10 min and blocked with 10% normal donkey serum (Yesean, Cat#36116ES10) in PBS for 1 hr at room temperature and incubated with primary antibodies (diluted with PBS) for 16 hr at 4°C. Primary antibodies and dilution rates are as follows: mouse anti-DDX4 (1:400, Abcam, Cat#ab27591); goat anti-FOXL2 (1:400, Novus, Cat#NB100-1277); rabbit anti-p-ERM (1:300, Cell Signaling Technology, Cat#3726T); rabbit anti-SMAD3 (1:200, Cell Signaling Technology, Cat#9523); rabbit anti-Ki67 (1:400, Cell Signaling Technology, Cat#D385). Next, ovarian sections were rinsed thoroughly with PBS for 1 hr and incubated with Alexa Fluor 488- or 555-conjugated secondary antibody (1:200, Yesean, Cat#33106ES60) for 1 hr at 37°C. Subsequently, the sections were again rinsed thoroughly with PBS, stained with Hoechst33342 (1:100, Sigma, Cat#14533) for 1 min, and sealed in anti-fade fluorescence mounting medium (Applygen, Cat#C1210) with microscope cover glass (Citoglas, Cat#10212450C). Sections were examined and photographed using a Nikon A1 confocal microscope.

For Ki67-positive GC analysis, the percentage was quantified as the number of GCs with positive signal divided by the total number of GCs per maximum follicular cross-section in the SFs of Ctrl and cKO mice. Sections were examined and photographed using a Nikon A1 confocal microscope.

### Early growing follicle isolation and culture in vitro

10- to 12-day-old ICR mice were obtained from Beijing Vital River Laboratory Animal Technology Co., Ltd. and housed at China Agricultural University. To collect the follicles, ovaries were dissected, discarded the interstitial tissues, and incubated in modified Leibovitz’s L-15 medium (Gibco 11415064) added with 1× penicillin-streptomycin (Gibco 15070063, 100×). The pre-AFs with indistinguishable antral structure based on relative size of 150–200 μm were confirmed using a stereomicroscope. Under the stereomicroscope, the ovaries were cut into small pieces using a 1 mL insulin syringe needle with the ‘cross-shaped division method’. Early growing follicles with a diameter of approximately 150–200 μm and five to six layers of granulosa cells were selected under the microscope, and then isolated from the ovarian tissue as intact individual follicles using the insulin needle. Early growing follicles for each experiment were collected from two to three mice. At least 20–30 follicles were collected for each group.

Isolated early growing follicles were cultured on 0.4 µm pore size inserts (Millipore, Cat#PICM0RG50) in six-well culture plates (NEST, Cat#703002) for 5 days. Basal culture medium comprised 1.6 mL MEM-α medium, 1× insulin-transferrin-sodium selenite media supplement (Sigma I3146, 100×), 25 mM NaHCO_3_, 5% fetal bovine serum (Gibco 16000-044), 1× penicillin-streptomycin, and 10 ng/mL follicle-stimulating hormone purchased from the National Hormone and Peptide Program. As a control, follicles were microinjected with *Matr3* siRNA (20 μM, Sigma-Aldrich, Cat#EMU014021) or NC siRNA (20 μM, GenePharma). Approximately half of the medium in each well was replaced with fresh medium every other day. Follicles were maintained at 37°C under 5% CO_2_ and 95% air.

### Oocyte isolation and culture in vitro

For GOs, the ovaries consisted of pre-AFs of 10- to 12-day-old mice were dissected in modified M2 medium (Millipore, MR-015-D). The ovaries were digested carefully in M2 medium containing 0.25 ng/mL collagenase type IV (Sigma-Aldrich, C5138), and the isolated GOs were cultured in M2. Upon the overnight culture in the M2, GOs containing an intact GV and indistinguishable zona pellucida were used for further assays.

For FGOs, mice at PD23 received intraperitoneal injections of 5 IU PMSG (Ningbo Sansheng Biological Technology, Cat#110251283). After 46 hr, the FGOs were released into the M2 medium. The GV-stage oocytes were manually collected using a mouth pipette and temporarily cultured in M2 medium supplemented with 2.5 μM milrinone (J&K Scientific, 245167). Only fully grown GV oocytes were selected for subsequent IVM. The selected oocytes were cultured in M16 medium (Millipore, MR-016) covered with mineral oil (Sigma, M8410) at 37°C. GV breakdown and the extrusion of the first polar body (MII) were observed and recorded at 2 and 16 hr, respectively.

### Plasmid construction

Full-length mouse *Matr3* cDNA was obtained from GO and cloned into the pcDNA3.1(+) vector using BamHI and XhoI endonuclease. To generate *Matr3*-EGFP, the EGFP open reading frame with a 14 amino acid N-terminal linker was amplified from pLV-EGFP-Cre by PCR using forward primer 5’- AAG GAA ACT GTG AGC AAG GGC GAG GAG C -3’ and reverse primer 5’- AGT GGA TCC GAG CTC GGT ACC CTA CTT GTA CAG CTC GTC CAT GCC -3’. To create pcDNA3.1(+)-*Matr3*-EGFP, the *Matr3* open reading frame from pCDNA3.1(+)-*Matr3* was amplified by PCR with forward primer 5’- GGG AGA CCC AAG CTG GCT AGC ATG TCC AAG TCA TTC CAG CAG TC-3’ and reverse primer 5’- CCC TTG CTC ACA GTT TCC TTC TTC TGC CTC CG -3‘, digested with NheI and KpnI, and inserted into the corresponding sites in pcDNA3.1(+). All constructs were confirmed by sequencing prior to transfection in HEK293T cells. Domain deletion mutants were described in previous research (Malik et al., 2018).

The HEK293T cell line was obtained from Abcam (Cat# ab282205, RRID:CVCL_0063). Cell identity was authenticated by short tandem repeat profiling. Mycoplasma contamination was monitored by PCR detection, and all cell lines were mycoplasma-negative. No cell lines from the ICLAC list of commonly misidentified cell lines were used in this study.

### In vitro transcription of mRNAs

Recombinant pcDNA3.1(+) plasmid containing the CDS of desired fragments was digested with XbaI. The DNA purification procedure was performed using TIANgel Midi Purification (TIANGEN BIOTECH, DP209) kit. Purified linearized plasmid templates were dissolved in RNase-free water and transcribed using the mMessage mMACHINE T7 ULTRA (Thermo Fisher Scientific, AM1345) kit to generate the capped mRNA with poly(A) tails. For removing unincorporated nucleotides and most proteins, the mRNA synthesis reactions were stopped and precipitated with 50 μL lithium chloride precipitation solution at –20°C overnight. On the following day, the mRNA pellet was centrifuged to remove the unincorporated nucleotides and LiCl and resuspended in the RNase-free water. The final mRNA concentration was determined via NanoDrop (Thermo Fisher Scientific). All the kits were used as per the manufacturer’s respective protocols.

### Oocyte immunofluorescence and confocal microscopy

The oocytes were fixed with 4% paraformaldehyde in PBS for 20 min at room temperature, followed by membrane permeabilization treatment with 0.5% Triton X-100 in PBS for 20 min at room temperature. After permeabilization, the oocytes were transferred into blocking buffer supplemented with 0.01% Triton X-100, 0.1% Tween 20, and 1% BSA for 1 hr at room temperature, and the oocytes were then incubated with primary antibodies at 4°C overnight. Primary antibodies and dilution rates are as follows: rabbit anti-MATR3 (1:400, Abcam, Cat#ab151714); rabbit anti-KDM3B (1:50, Abcam, Cat#ab70797); rabbit anti-p-ERM (1:300, Cell Signaling Technology, Cat#3726T); mouse anti-α-Tubulin-488 (1:100, Abcam, Cat#ab195887); mouse anti-H3K9me2 (1:200, Abcam, Cat#ab1220); rabbit anti-H3K27me3 (1:100, Active Motif, Cat#39155); rabbit anti-H3K9me1 (1:100, Abcam, Cat#ab176880); mouse anti-H3K9me3 (1:200, Active Motif, Cat#61013); rabbit anti-H3K4me3 (1:300, Abcam, Cat#ab8580); goat anti-GDF9 (1:100, Biotechne, Cat#AF739). Following the removal of the primary antibodies, the oocytes were washed three times in PBS washing buffer with 0.01% Triton X-100 and 0.1% Tween 20. For the secondary antibodies and staining, Alexa Fluor Plus 488 donkey anti-goat IgG (H+L) secondary antibody (Invitrogen, A32816) was used at a dilution of 1:100 for 2 hr at room temperature. After the secondary antibody incubation, oocytes were washed in washing buffer three times, and DNA was stained with Hoechst 33342 (1 μg/mL in blocking buffer) for 10 min at room temperature. Finally, the oocytes were dispersed and mounted on glass slides with DABCO-containing blocking buffer and analyzed by laser-scanning confocal microscopy.

### RT-qPCR

Total RNA was isolated from ovaries with TRIzol (Invitrogen, Cat#15596018). The quantity and quality of total RNA were determined using NanoDrop. 1 μg of total RNA of each sample was reverse-transcribed into cDNA according to the manufacturer’s recommendation (Takara, Cat#RR047A). For oocyte, 50–100 oocytes were collected as one sample. Each sample was reverse-transcribed into cDNA according to the manufacturer’s recommendation (TRAN, Cat#AT301). RT-qPCR was performed in 96-well plates (Roche, Cat#04729692001) using FastStart Universal SYBR Green Master (Roche, Cat#61396600) with LightCycler 96 Real-Time PCR System (Roche). Reaction parameters were as follows: 10 min at 95°C, followed by 45 cycles of 10 s at 95°C and 30 s at 60°C. Data were normalized to *Actb*. Primers are presented in Supplementary file 2.

### Western blotting

Total protein from ovaries was extracted with TRIzol (Invitrogen, Cat#15596018). For oocyte, 250 GOs or 200 FGOs were collected as one sample. Protein was separated on 10% sodium dodecyl sulfate-polyacrylamide gel electrophoresis (SDS-PAGE) and transferred to PVDF (polyvinylidene fluoride) membranes (Millipore, Cat# IPVH00). The membranes were blocked in 5% skim milk (Solarbio, Cat#D8340) for 1 hr at room temperature and incubated with relevant primary antibodies (diluted with TBST [TBS plus 0.05% Tween-20]) overnight at 4°C. After rinsing with TBST, the membranes were incubated with the HRP-linked secondary antibody (1:4000, ZSGB-BIO, Cat#ZB-2301/ZB-2305) for 1 hr at room temperature and rinsed again with TBST. The membranes were visualized using the SuperSignal detection system (Thermo Fisher Scientific, Prod 34080). The image was quantified using Adobe Photoshop CS6. Primary antibodies and dilution rates were as follows: rabbit anti-MATR3 (1:1000, Abcam, Cat#ab151714); rabbit anti-KDM3B (1:500, Abcam, Cat#ab70797); rabbit anti-GAPDH (1:1000, Proteintech, Cat#10494); rabbit anti-RNAPII (1:10,000, Abcam, Cat#ab5095); goat anti-FOXL2 (1:500, Novus, Cat#NB100-1277); rabbit anti-β-Actin (1:10,000, Abmart, Cat#T40104); rabbit anti-GDF9 (1:500, Abcam, Cat#ab38544); rabbit anti-PCNA (1:500, Beyotime Biotechnology, Cat#AF1363).

### scRNA-seq

GO of NC and si-*Matr3* were collected one by one, and RNA was extracted by a commercial RNA extraction kit (Vazyme, Cat#N712). cDNA libraries were sequenced on the NovaSeq 6000 Illumina sequencing platform and analyzed by Novogene Co., Ltd. (Beijing, China). DEGs were analyzed using the DESeq2 R package (1.20.0). Generally, genes with p-values less than 0.05 and absolute fold-change larger than 2 were considered DEGs.

### LACE-seq

GOs were isolated from the ovaries of 10- to 12-day-old mice for LACE-seq. Oocytes were washed three times with cold PBS and immediately irradiated on ice with UV light at 400 mJ/cm^2^ for two times. The UV light-treated oocytes were stored at −80°C until 500 oocytes required for one experiment were accumulated. LACE‐seq was carried out as described previously, with the same experiment repeated independently twice.

### RNA immunoprecipitation

Ovaries were isolated from the ovaries of 10- to 12-day-old mice for RIP. RIP assays were performed by using the EZ-Magna RIP RBP Immunoprecipitation Kit (Millipore; 17-701) following the manufacturer’s protocol. The first-strand RIP cDNA libraries were synthesized using the M-MLV synthesis kit (Invitrogen, C28025-032) with random primers. Quantitative real-time PCR assay was performed using FastStart Universal SYBR Green Master (ROX) (Roche, 04913914001), and primers related to RT-PCR are shown in Supplementary file 2. The fold enrichment of *Rdx* in the anti-MATR3 antibody-precipitated ovaries was determined relative to the IgG-precipitated sample.

### Co-immunoprecipitation

The antibodies and beads were combined and incubated overnight in a shaker at 4°C. HEK293T cells (Abcam, Cat#ab282205, RRID:CVCL_0063) were collected and lysed in RIPA lysate with PMSF. Then the supernatant was combined with beads that bind antibodies and kept overnight at 4°C. The beads were then eluted with the eluents in the Immunoprecipitation Kit (Invitrogen, 10006D). The precipitates were heated in the SDS-PAGE sample buffer, and western blotting was performed.

### Statistical analysis

All experiments were repeated at least three times. Results were expressed as the mean ± SD. Statistical analyses were conducted using GraphPad Prism 9 software (GraphPad Software, La Jolla, CA, USA). The statistical significance of the differences between the groups was measured by a two-sided ANOVA test. The statistical significances were defined as: *, p<0.05; **, p<0.01; ***, p<0.001; n.s., non-significant.

### Study approval

All experiments were endorsed by the General Hospital of Ningxia Medical University, No. KYLL-2021-758 and complied with the Declaration of Helsinki. Oocytes were retrieved from female patients of Chinese Han ethnicity (East Asian ancestry) undergoing ovarian stimulation for IVF at the General Hospital of Ningxia Medical University. All participating couples provided informed consent for the use of their COCs and/or sperm to create embryos for research purposes. In the clinical IVF laboratory, embryologists randomly selected donated COCs that had failed to fertilize and remained at the GV stage, which were then transported to the research laboratory. Mice were housed in mouse facilities under 12/12 hr light/dark cycles at 26°C and 40–70% humidity with access to chow and water ad libitum, according to the guidelines for the care and use of laboratory animals. All procedures were conducted in accordance with the guidelines of the Animal Research Committee of the China Agricultural University and were approved under approval No. AW71014202-3-1. All animal experiments involved ethical and humane treatment.

## Data Availability

All data generated or analyzed during this study are included in the manuscript and supporting files; source data files have been provided for all figures.

## Additional files

Supplementary file 1. gRNA and primer sequences for *Matr3* knockout mouse construction and genotyping.

Supplementary file 2. RT-PCR primer sequences for target gene detection.

MDAR checklist
