# Supplementary material for: MATR3 is essential for oocyte growth and maturation quality through a dual molecular mechanism
Source: eLife. 2026 Sep 21;15:RP110703. doi: 10.7554/eLife.110703 (PMC13592816; doi:10.7554/eLife.110703)
Supplement: Supplementary file 2 [file elife-110703-supp1.docx]

| gRNA | Sequence |
| --- | --- |
| gRNA-1 for *Matr3* ^flox/flox^ mice | TTAATACGACTCACTATAGGCACCCAAACATCTCACCCTGTTTTAGAGGCTAGAAATAG |
| gRNA-2 for *Matr3* ^flox/flox^ mice | TTAATACGACTCACTATAGGAAAGCACATTGACTTCAGGGTTTTAGAGGCTAGAAATAG |
| Primers for cloning | Sequence |
| Matr3-SalI-F | ACGCGTCGACTTTCATCCACTGTTCACTAACTCTTCTCTTC |
| Matr3-BamHI-F | GATGGATCCGGTTGGGTGAGATGTTTGGGTGCATCAA |
| Matr3-BamHI-Loxp1-F1 | TGCTATACGAAGTTATCGCCGAGTCCCTTGGATTTAAATGTTTTTAAGCTCAA |
| Matr3-BamHI-Loxp1-F2 | GATGGATCCATAACTTCGTATAATGTATGTATGCTATACGAAGTTATCGCCGAGTCCC |
| Matr3-Nhe-Loxp2-R1 | CATACATTATACGAAGTTATGCGAGAGGGGAAAAATGCTTAAAACAATTCAACA |
| Matr3-Nhe-Loxp2-R2 | ATAGCTAGCATAACTTCGTATAGCATACATTATACGAAGTTATGCGAGAGG |
| Matr3-NheI-F | ATAGCTAGCCTGTCCTGAAGTCAATGTGCTTTCCATTTAGG |
| Matr3-NheI-R | GGAAAAGCGGCCGCAAAGATTTACAGTTCTTTAAGATCATTTTCATT |
| Matr3-screen-F1 | GCCTGCTGTCCTTTGTGGCATTTT |
| Matr3-screen-R1 | ACATTTAAATCCAAGGGACTCGGCG |
| Matr3-screen-R2 | GGAAAGCACATTGACTTCAGGACAGG |
| Matr3-screen-F3 | CACCCAAACATCTCACCCAACCG |
| Matr3-screen-F4 | TGTTTTAAGCATTTTTCCCCTCTCGC |
| Matr3-screen-R3 | TCTGACAATCCTCTTATGTTCCTTTCTTCC |
| Primers for genotyping | Sequence |
| *Matr3*-F | GAAGAAGCCCATCAATGTTTTCACTGCAC |
| *Matr3*-R | AAGGACTGTAGAATCTTCTTCAAGCTGAG |
| *Gdf9*-F | TCTGATGAAGTCAGGAAGAACC |
| *Gdf9*-R | GAGATGTCCTTCACTCTGATTC |
