# Supplementary material for: MATR3 is essential for oocyte growth and maturation quality through a dual molecular mechanism
Source: eLife. 2026 Sep 21;15:RP110703. doi: 10.7554/eLife.110703 (PMC13592816; doi:10.7554/eLife.110703)
Supplement: Supplementary file 3 [file elife-110703-supp2.docx]

| Gene | Forward Primer | Reverse Primer |
| --- | --- | --- |
| *Matr3* | TTGAGAAAAAGAGGGGCGCT | TCGACGACTGTGACTTGCTC |
| *Gdf9* | TCACCTCTACAATACCGTCCGG | GAGCAAGTGTTCCATGGCAGTC |
| *Rdx* | TCAGTGTGACCTTCTCATGCC | AGTCCCATGTCTTGTCTGTGG |
| *Actb* | CATTGCTGACAGGATGCAGAAGG | TGCTGGAAGGTGGACAGTGAGG |
