# Supplementary material for: MATR3 is essential for oocyte growth and maturation quality through a dual molecular mechanism
Source: eLife. 2026 Sep 21;15:RP110703. doi: 10.7554/eLife.110703 (PMC13592816; doi:10.7554/eLife.110703)
Supplement: Supplementary file 4 [file elife-110703-fig1-data1.zip › Figure 1-source data 1.pdf]

GAPDH:

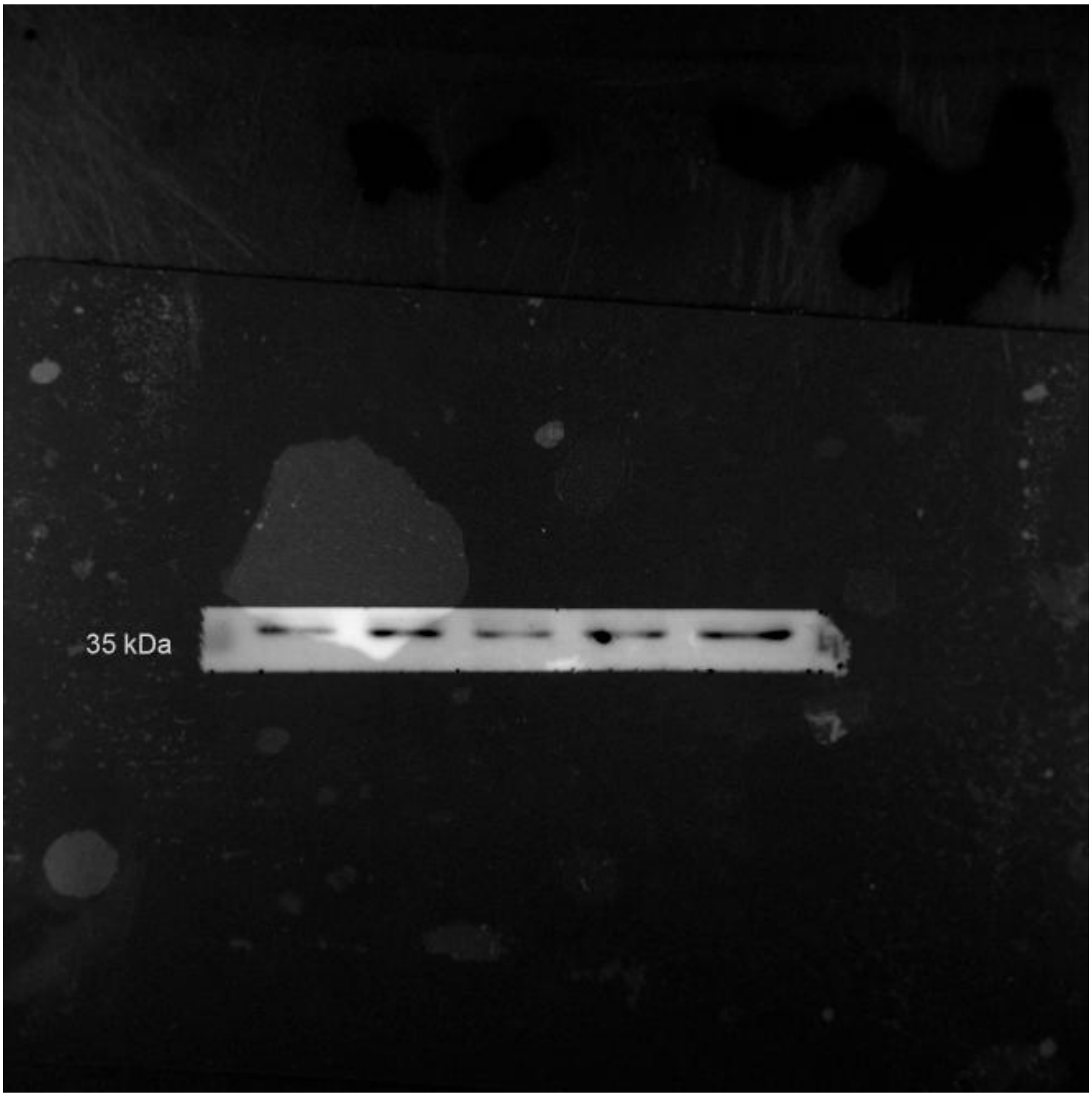

MATR3:

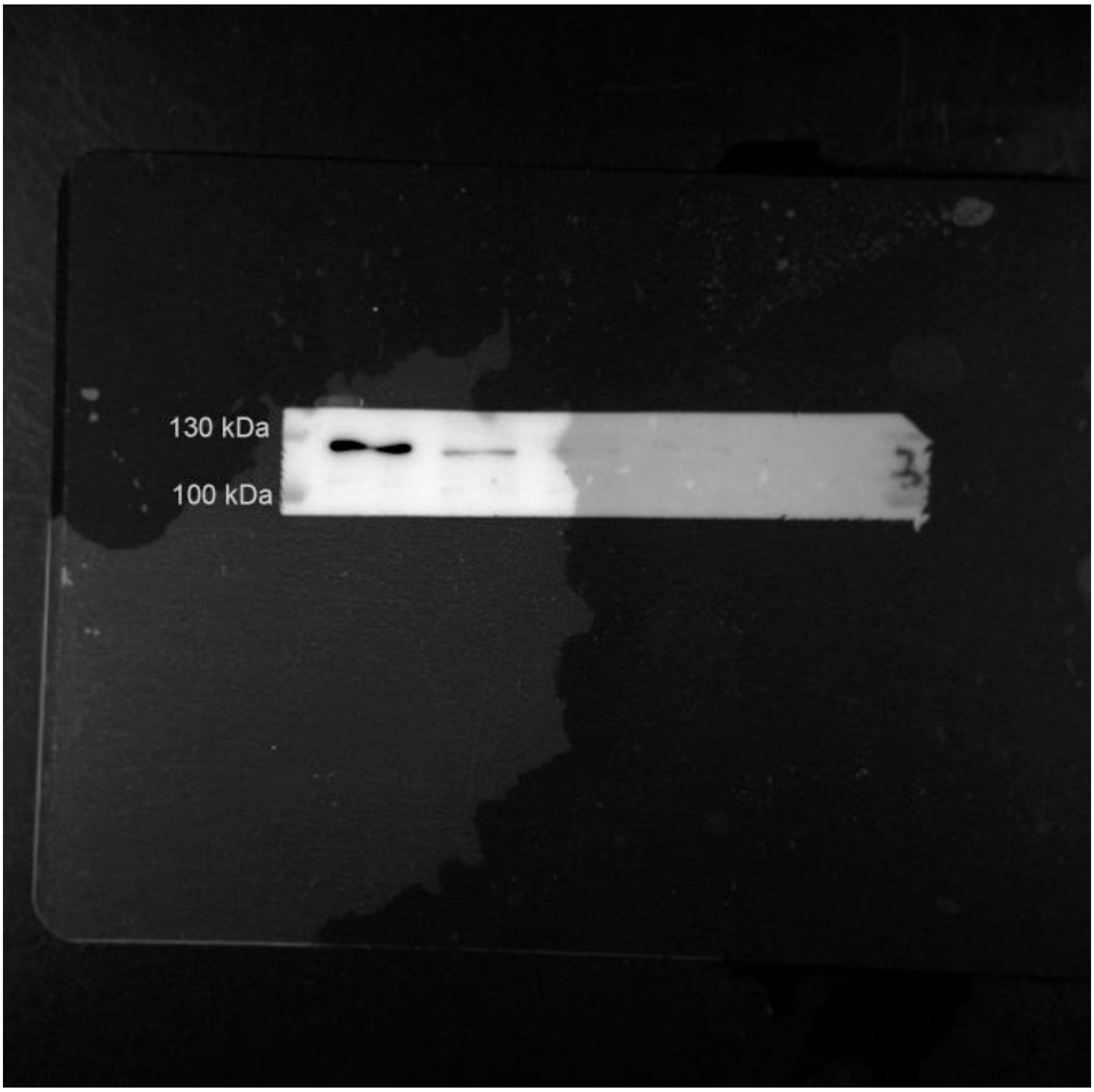

Figure 1, Source Data 1. Original membranes corresponding to Figure 1, panel B.
Lanes correspond to oocytes at the GO, FGO, GVBD, MI and MII stages, with 200 oocytes loaded per lane. The 180 kDa Plus Prestained Protein Marker (Vazyme MP201) was employed.
