# Supplementary material for: MATR3 is essential for oocyte growth and maturation quality through a dual molecular mechanism
Source: eLife. 2026 Sep 21;15:RP110703. doi: 10.7554/eLife.110703 (PMC13592816; doi:10.7554/eLife.110703)
Supplement: Supplementary file 6 [file elife-110703-fig1-figsupp1-data1.zip › Figure 1-figure supplement 1-source data 1.pdf]

β-ACTIN:

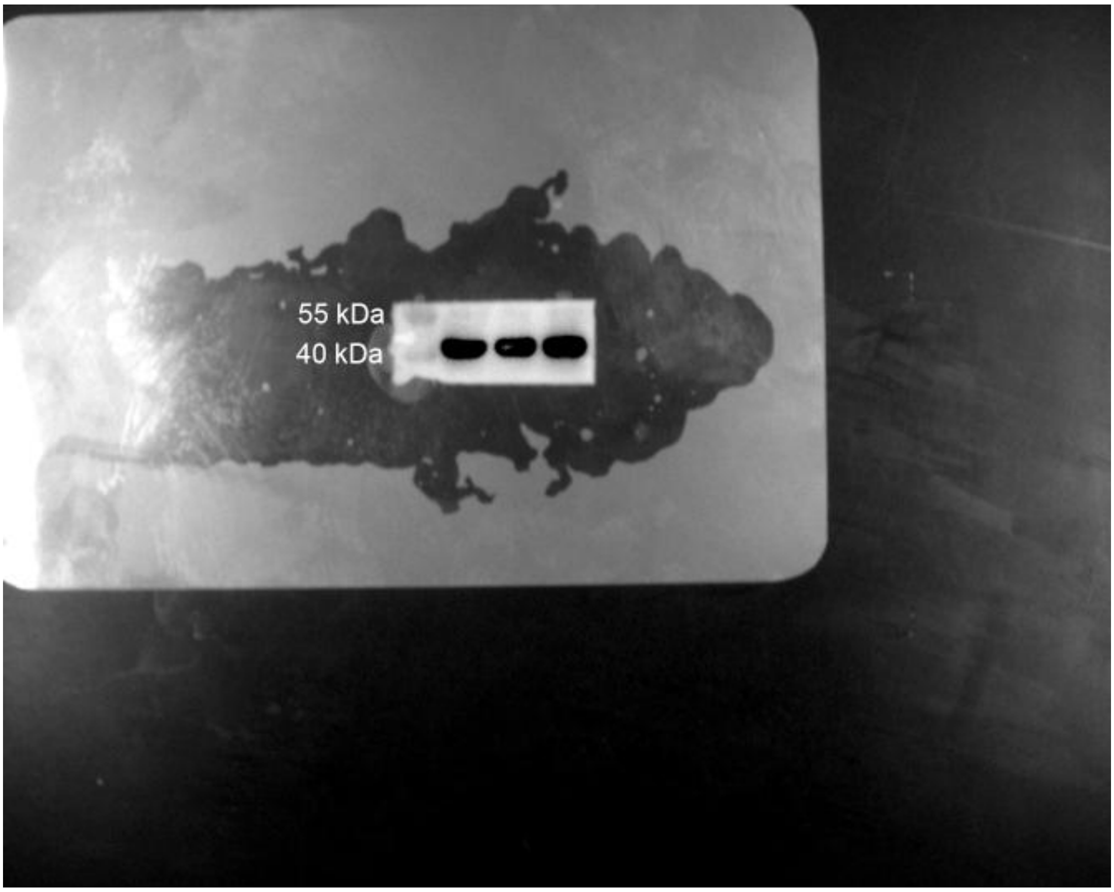

MATR3:

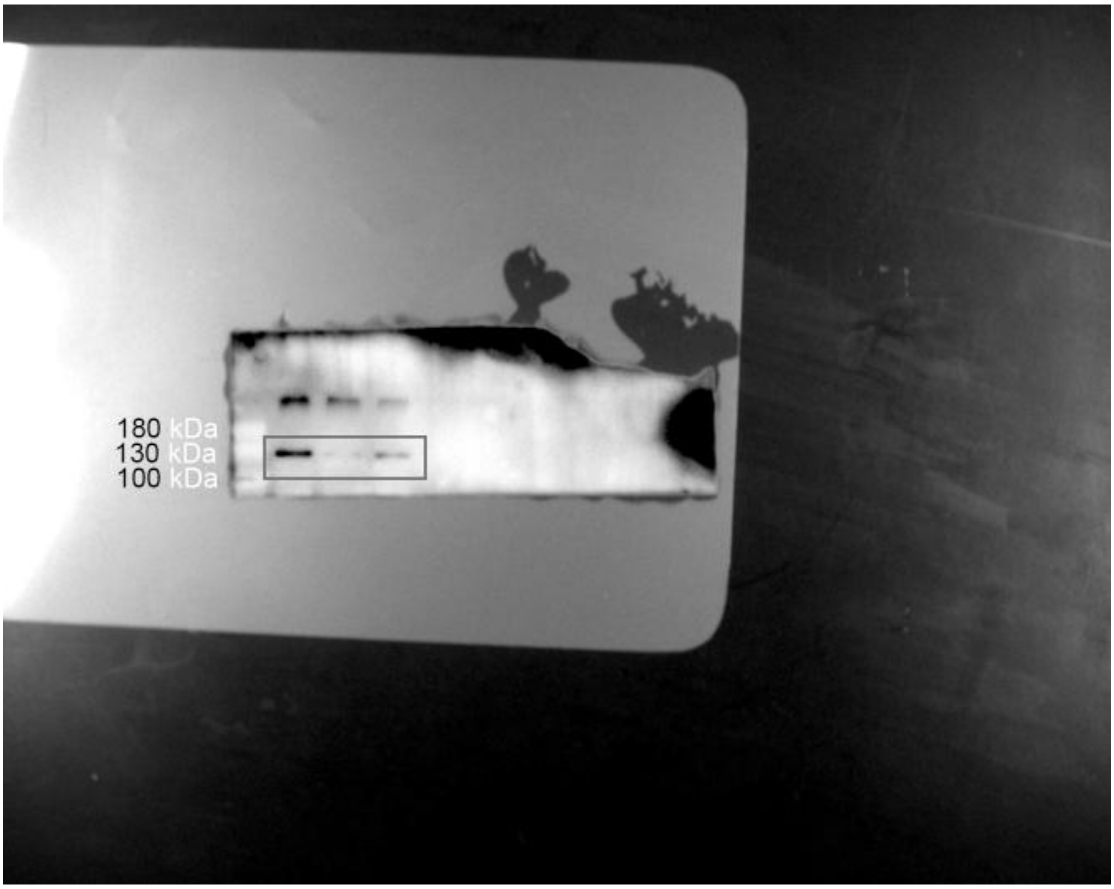

Figure 1-figure supplement 1-Source Data 1. Original membranes corresponding to Figure 1- figure supplement 1, panel C.

Lanes correspond to granulosa cells from porcine follicles of < 2 mm, 2-6 mm and > 6 mm. The 180 kDa Plus Prestained Protein Marker (Vazyme MP201) was employed.
