# Supplementary material for: MATR3 is essential for oocyte growth and maturation quality through a dual molecular mechanism
Source: eLife. 2026 Sep 21;15:RP110703. doi: 10.7554/eLife.110703 (PMC13592816; doi:10.7554/eLife.110703)
Supplement: Supplementary file 8 [file elife-110703-fig1-figsupp1-data3.zip › Figure 1-figure supplement 1-source data 3.pdf]

β-ACTIN:

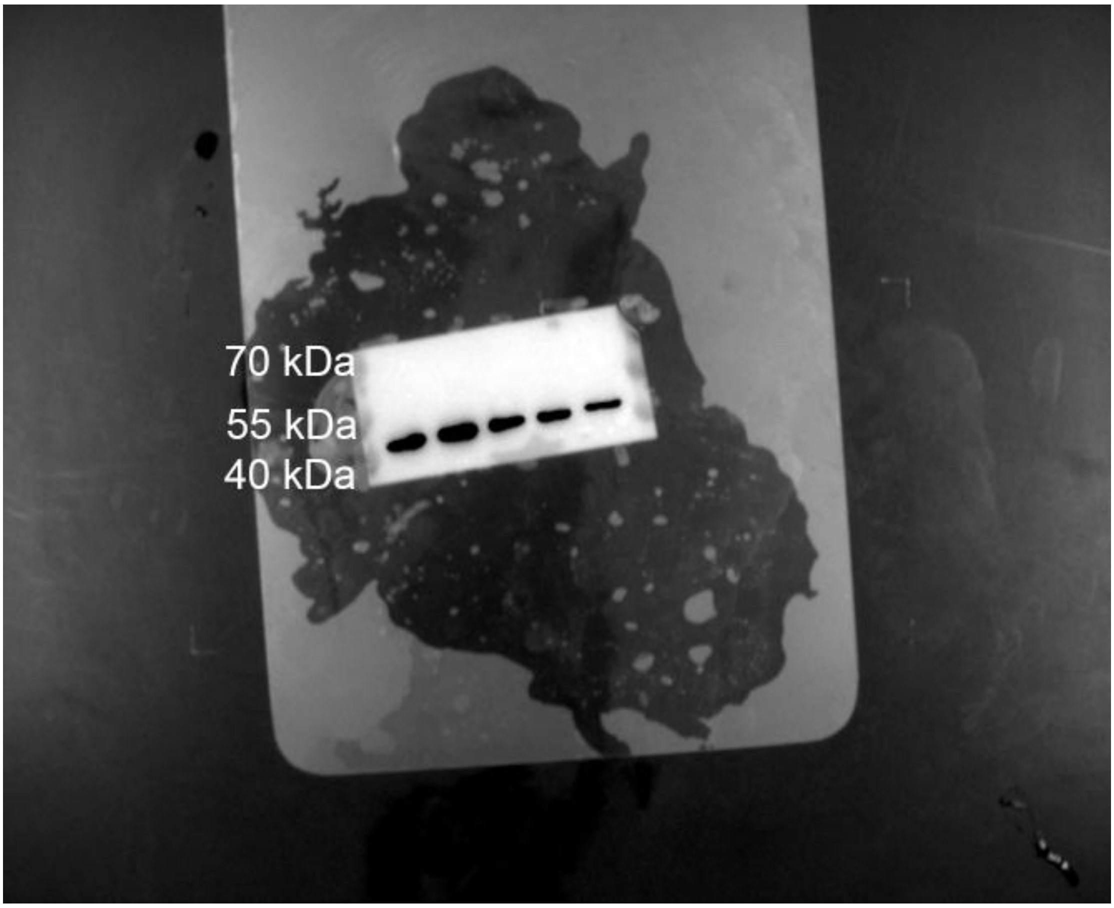

MATR3:

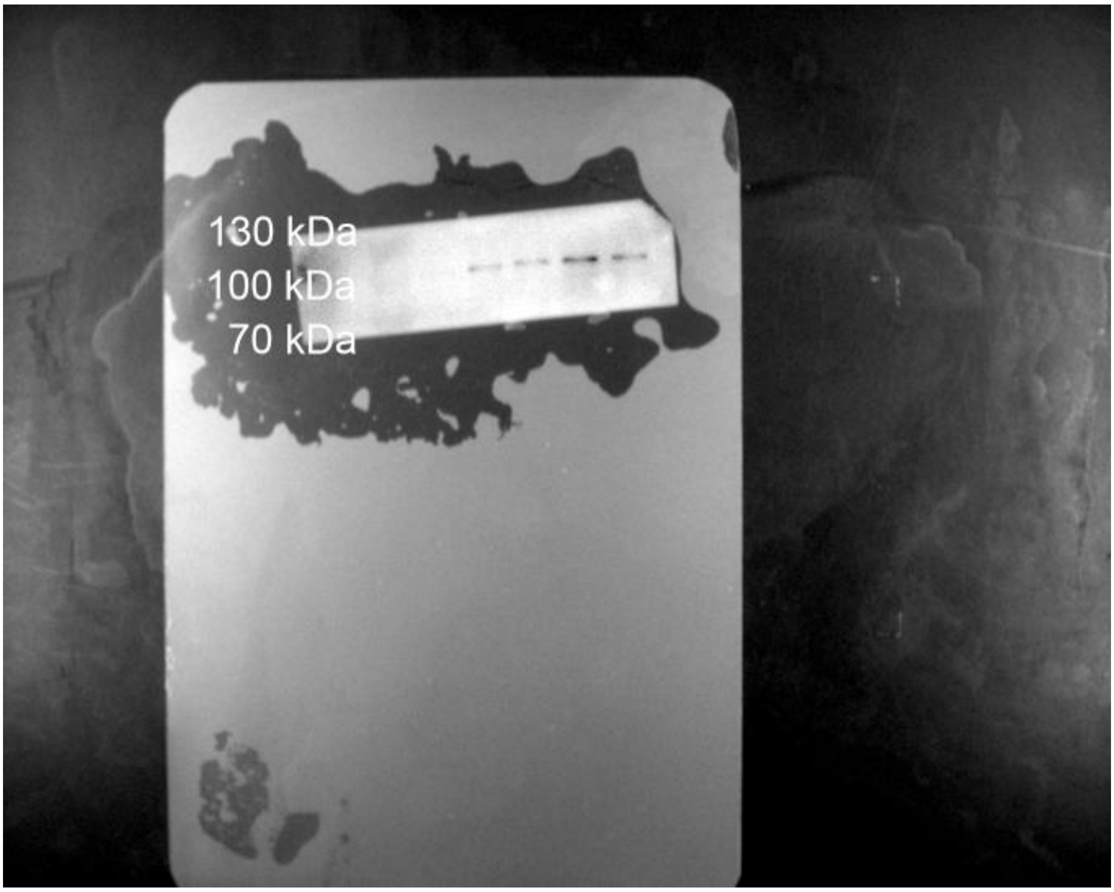

Figure 1-figure supplement 1-Source Data 3. Original membranes corresponding to Figure 1-figure supplement 1, panel D.

Lanes correspond to untreated porcine ovarian granulosa cells (Control), granulosa cells after FSH treatment for 48 h (FSH 48 h), and granulosa cells treated with LH for 1, 2 and 3 h following 48 h of FSH pretreatment (LH 1 h, LH 2 h, LH 3 h). The 180 kDa Plus Prestained Protein Marker (Vazyme MP201) was employed.
