# Supplementary material for: MATR3 is essential for oocyte growth and maturation quality through a dual molecular mechanism
Source: eLife. 2026 Sep 21;15:RP110703. doi: 10.7554/eLife.110703 (PMC13592816; doi:10.7554/eLife.110703)
Supplement: Supplementary file 10 [file elife-110703-fig1-figsupp2-data1.zip › Figure 1-figure supplement 2-source data 1.pdf]

β-ACTIN:

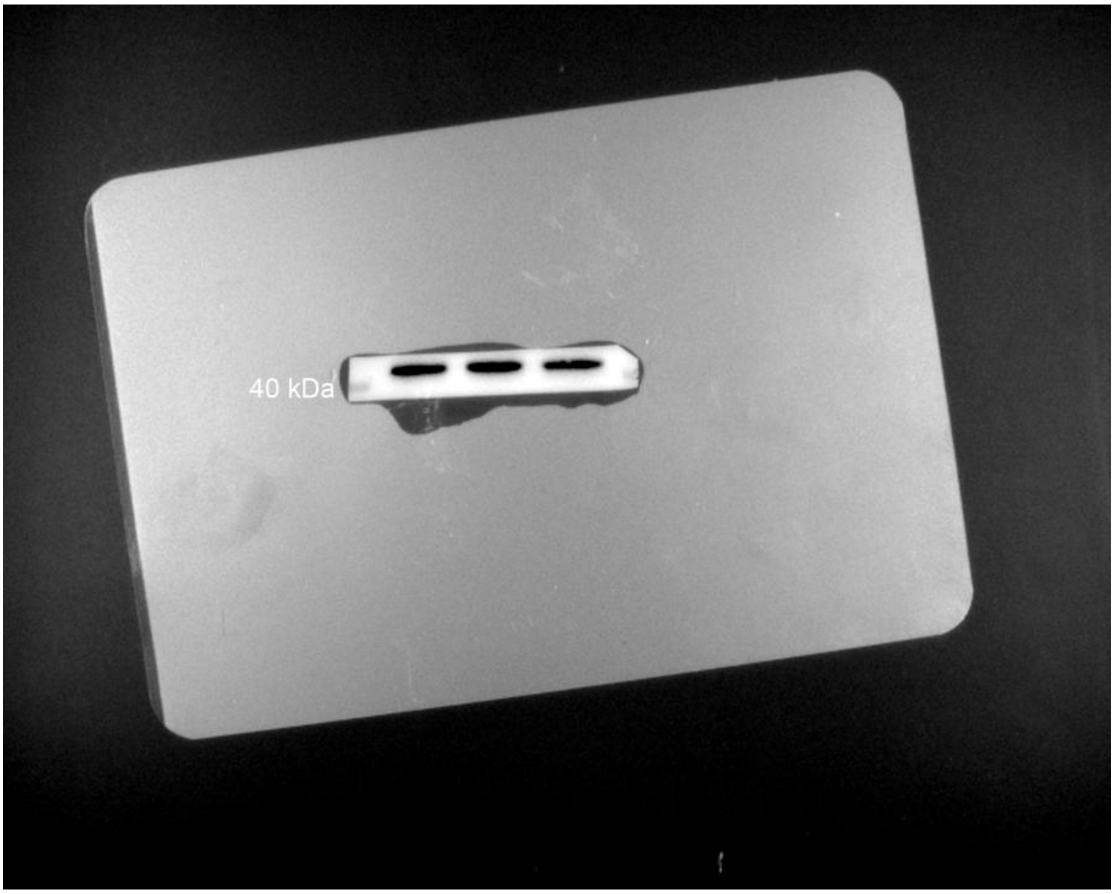

MATR3:

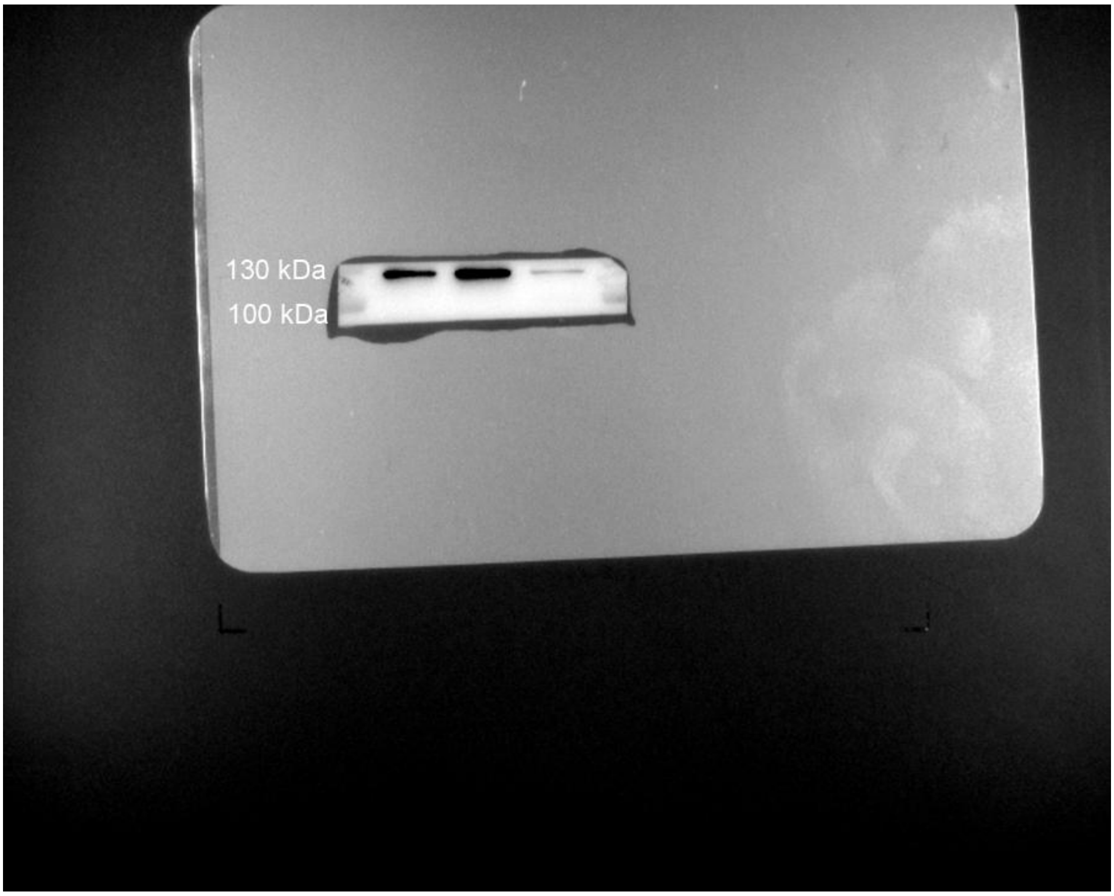

Figure 1-figure supplement 2-Source Data 1. Original membranes corresponding to Figure 1- figure supplement 2, panel B.

Lanes correspond to untreated cells, cells transfected with Negative Control for 48 h, and cells transfected with Matr3 siRNA for 48 h. The 180 kDa Plus Prestained Protein Marker (Vazyme MP201) was employed.
