# Supplementary material for: MATR3 is essential for oocyte growth and maturation quality through a dual molecular mechanism
Source: eLife. 2026 Sep 21;15:RP110703. doi: 10.7554/eLife.110703 (PMC13592816; doi:10.7554/eLife.110703)
Supplement: Supplementary file 12 [file elife-110703-fig1-figsupp2-data3.zip › Figure 1-figure supplement 2-source data 3.pdf]

β-ACTIN:

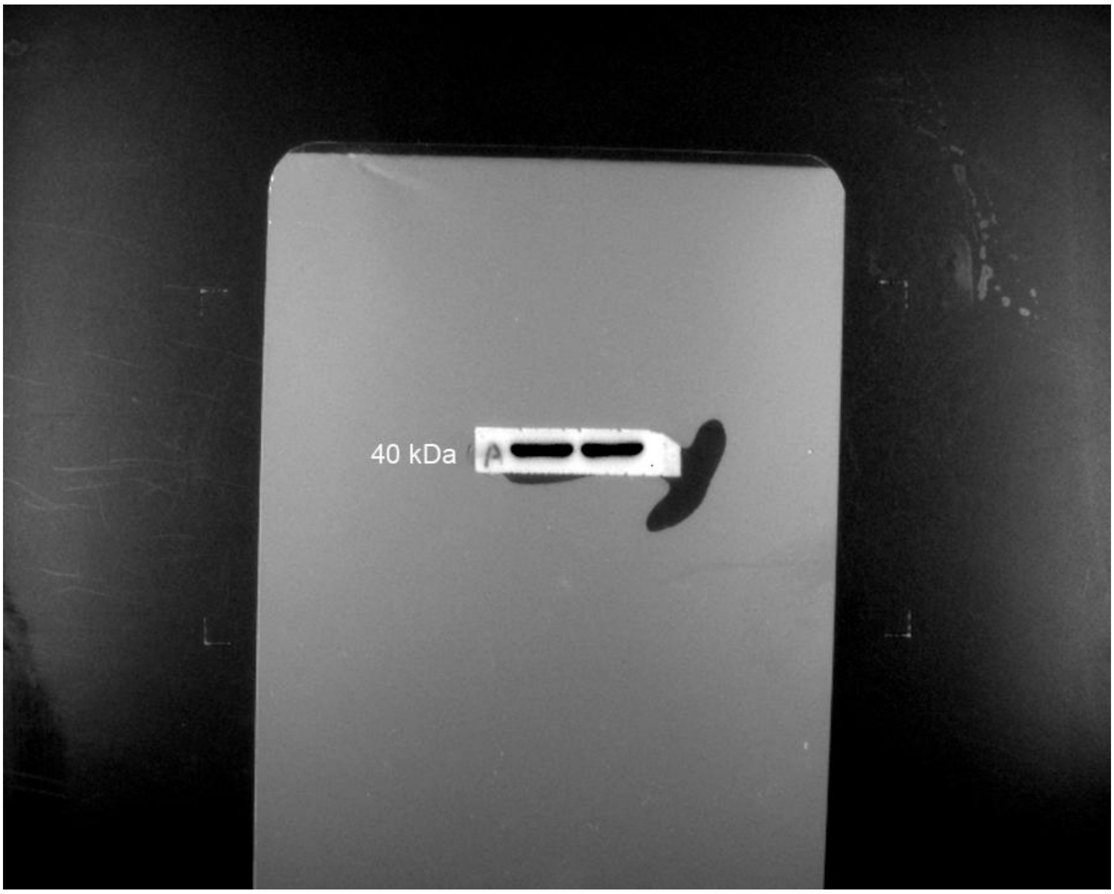

FOXL2:

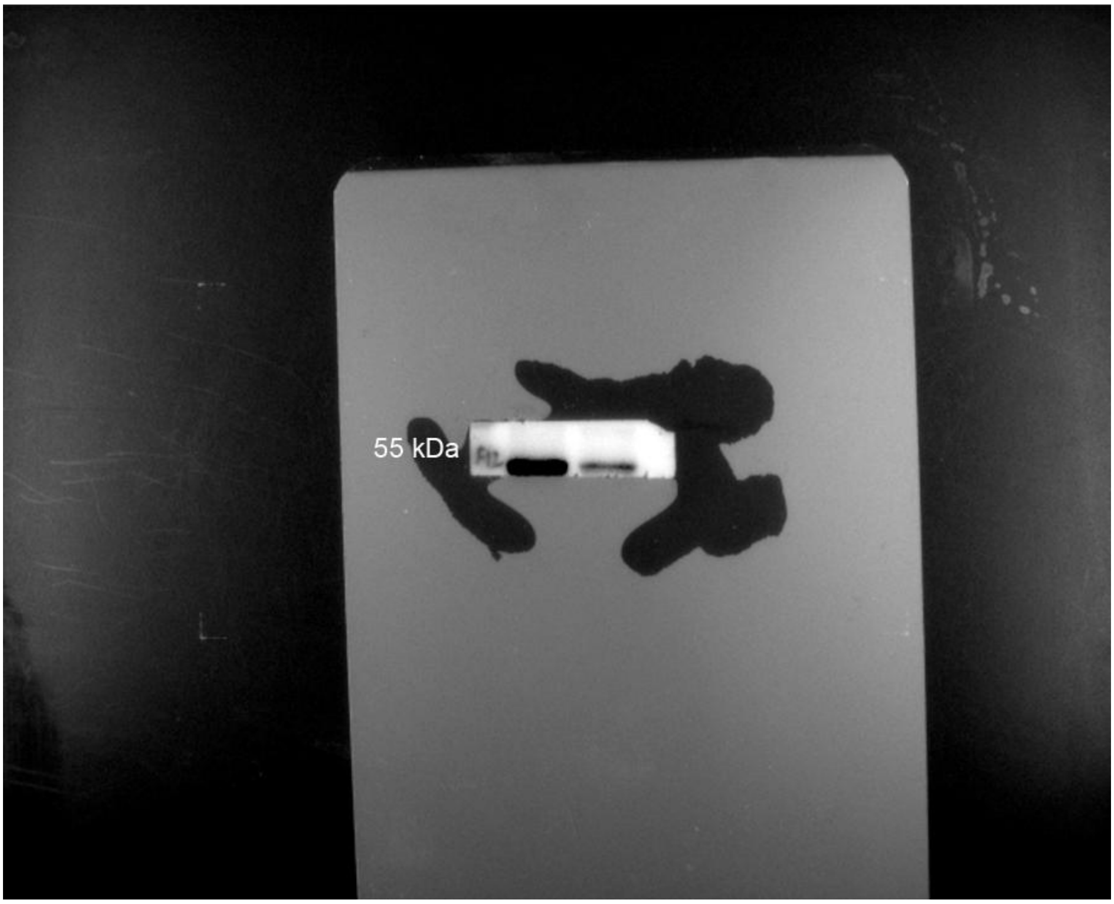

IGFIR:

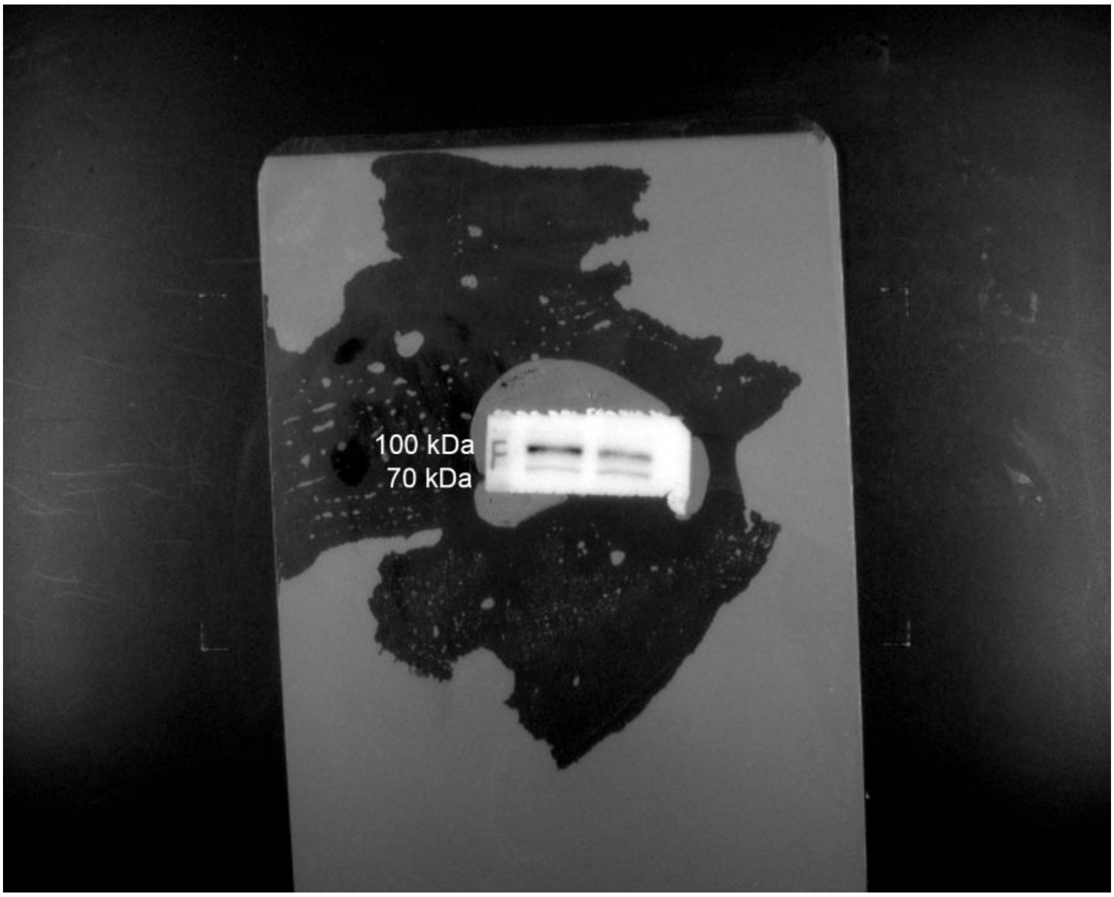

PCNA：

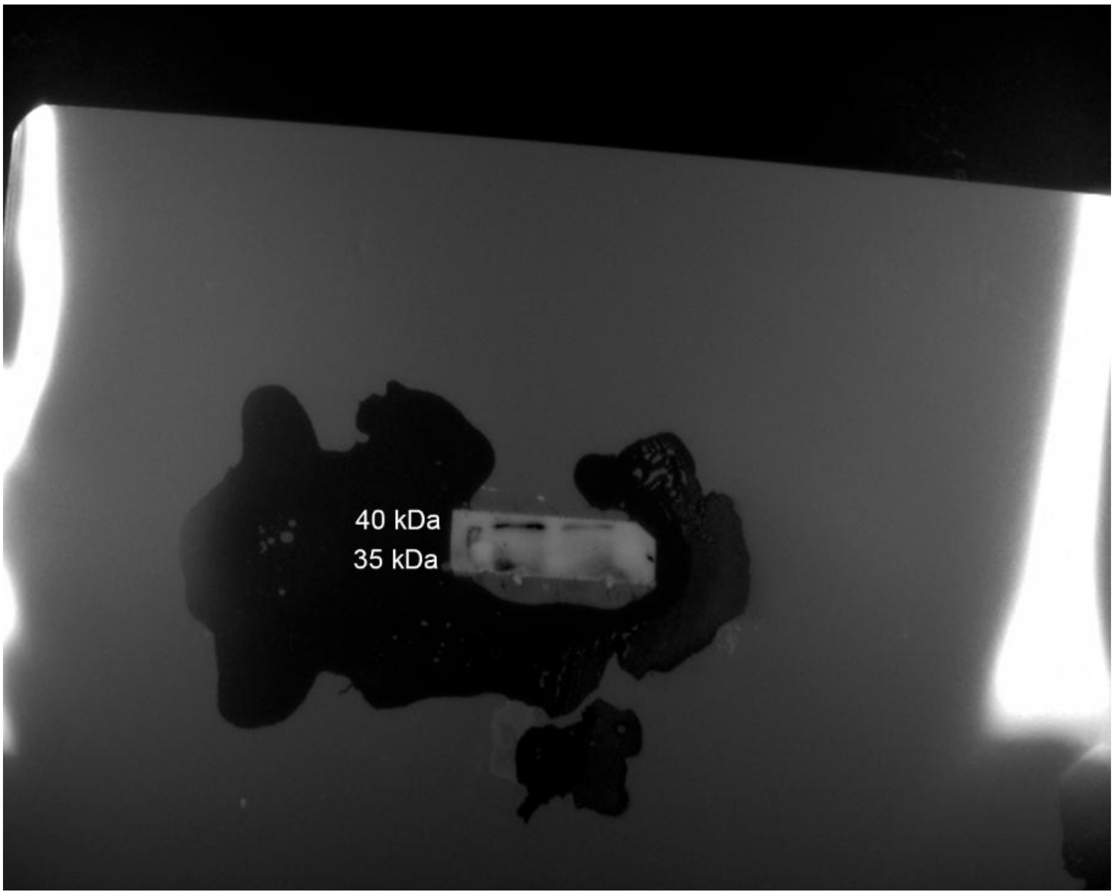

Figure 1-figure supplement 2-Source Data 3. Original membranes corresponding to Figure 1- figure supplement 2, panel E.

Lanes correspond to granulosa cells derived from preantral follicle oocytes injected with Negative Control and those injected with *Matr3* siRNA after 5 days of culture. The 180 kDa Plus Prestained Protein Marker (Vazyme MP201) was employed.
