# Supplementary material for: MATR3 is essential for oocyte growth and maturation quality through a dual molecular mechanism
Source: eLife. 2026 Sep 21;15:RP110703. doi: 10.7554/eLife.110703 (PMC13592816; doi:10.7554/eLife.110703)
Supplement: Supplementary file 14 [file elife-110703-fig1-figsupp3-data1.zip › Figure 1-figure supplement 3-source data 1.pdf]

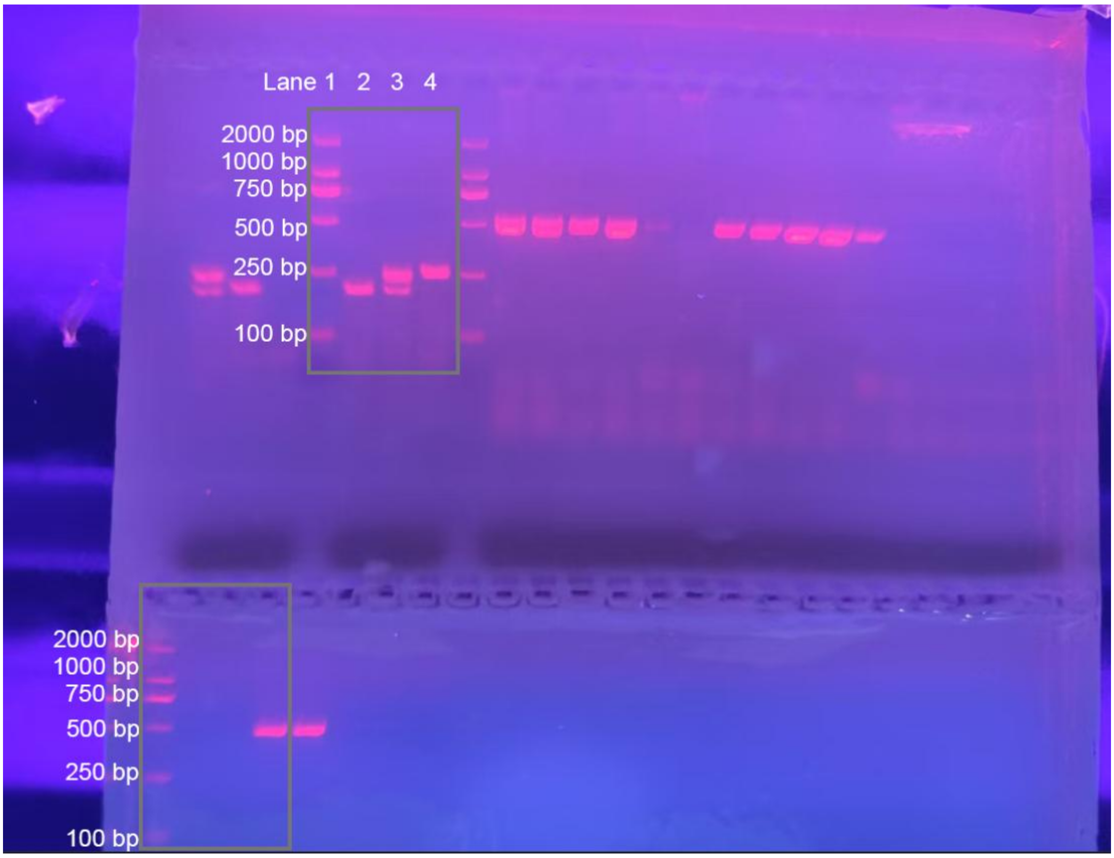

Figure 1-figure supplement 3-Source Data 1. Original membranes corresponding to Figure 1-figure supplement 3, panel A.

Lanes 1 and 5 correspond to DNA marker DL2000. Lanes 2 and 6 correspond to DNA from WT mice. Lanes 3 and 7 correspond to DNA from *Matr3*$^{flox/+}$ mice. Lanes 4 and 8 correspond to DNA from *Matr3*$^{flox/flox}$; *Gdf9*-Cre mice. The remaining lanes contain samples unrelated to this experiment.
