# Supplementary material for: MATR3 is essential for oocyte growth and maturation quality through a dual molecular mechanism
Source: eLife. 2026 Sep 21;15:RP110703. doi: 10.7554/eLife.110703 (PMC13592816; doi:10.7554/eLife.110703)
Supplement: Supplementary file 16 [file elife-110703-fig3-data1.zip › Figure 3-source data 2.pdf]

β-Actin:

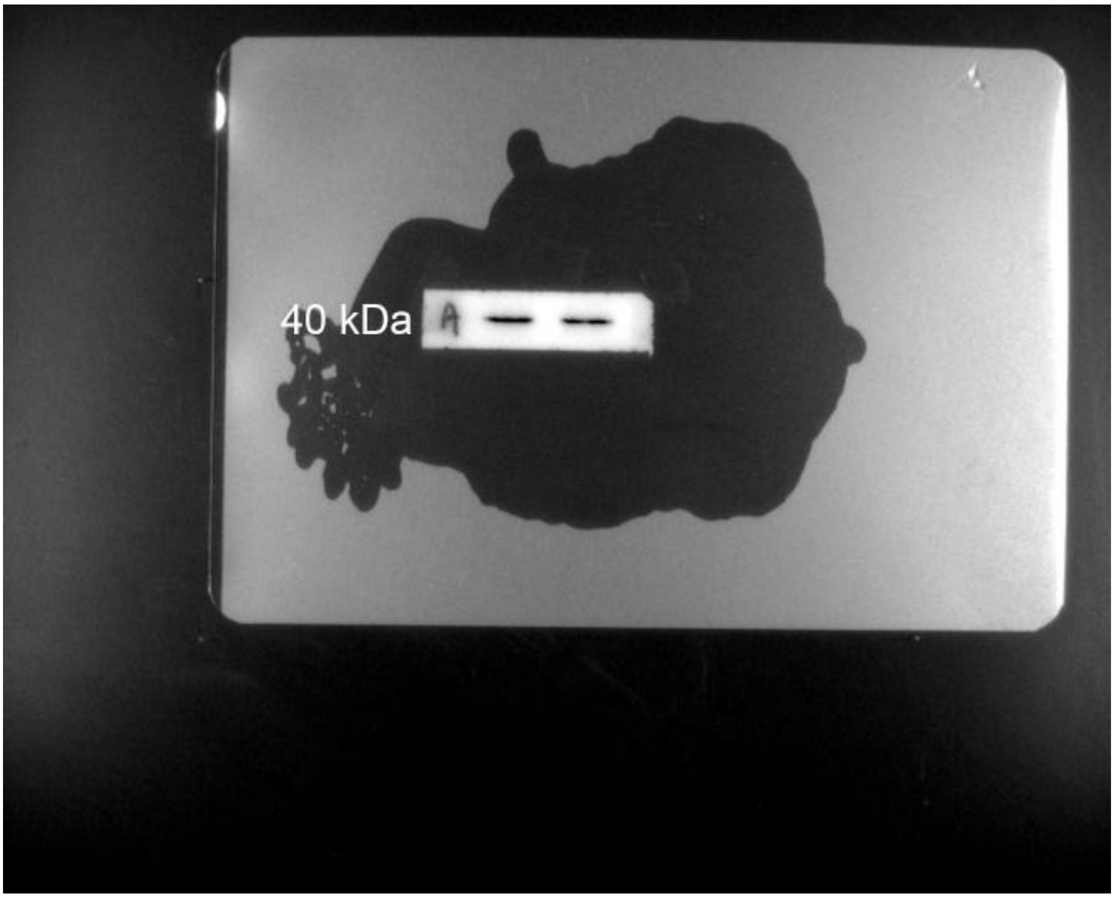

RNAPII:

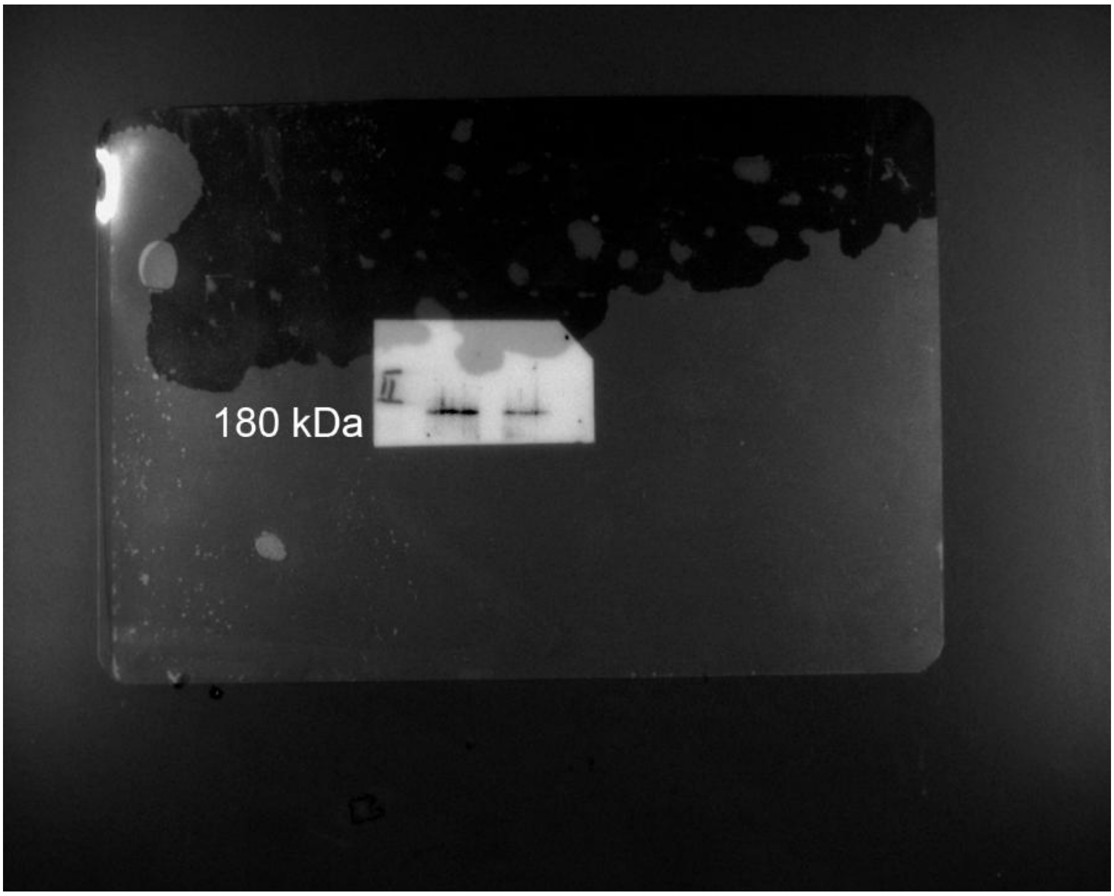


Figure 3, Source Data 1. Original membranes corresponding to Figure 3, panel G.
Lanes correspond to growing oocytes injected with Negative Control or *Matr3* siRNA, with 200 oocytes loaded per lane. The 180 kDa Plus Prestained Protein Marker (Vazyme MP201) was employed.
