# Supplementary material for: MATR3 is essential for oocyte growth and maturation quality through a dual molecular mechanism
Source: eLife. 2026 Sep 21;15:RP110703. doi: 10.7554/eLife.110703 (PMC13592816; doi:10.7554/eLife.110703)
Supplement: Supplementary file 18 [file elife-110703-fig4-data1.zip › Figure 4-source data 1.pdf]

β-Actin:

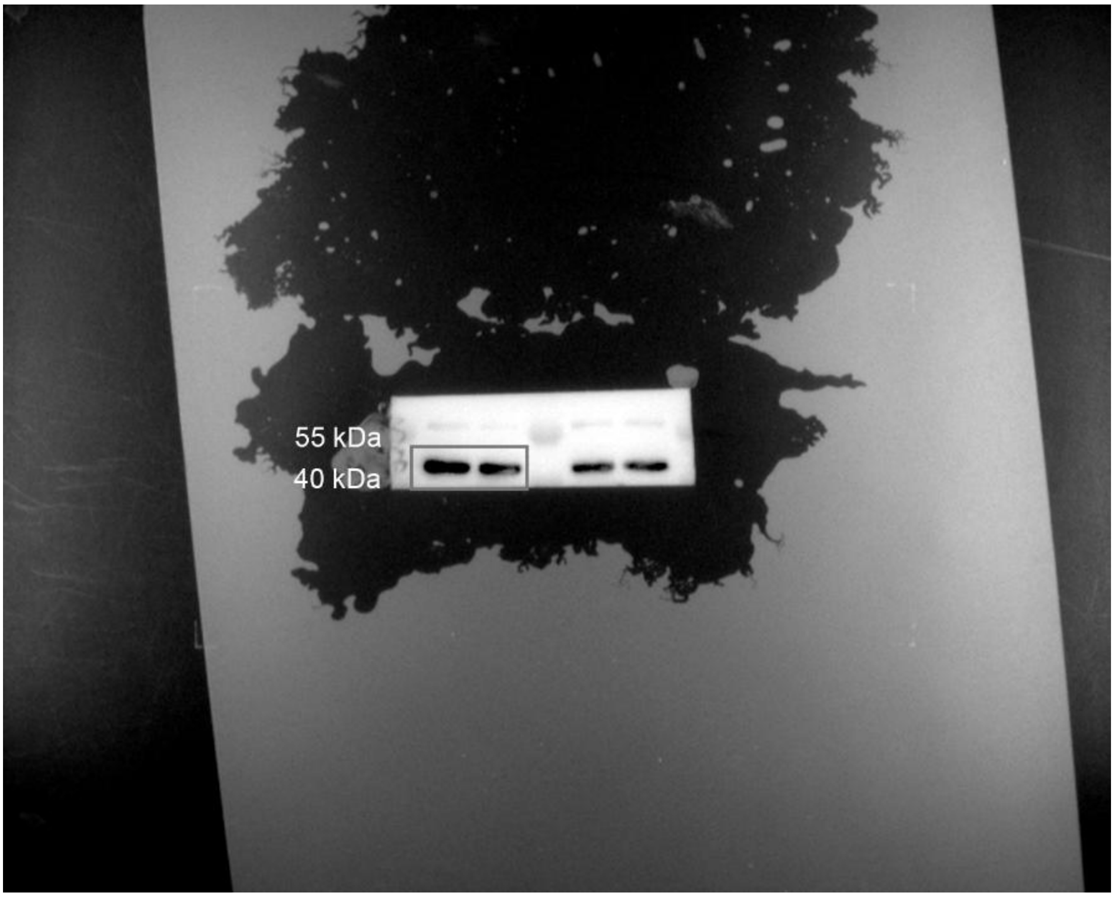

GDF9:

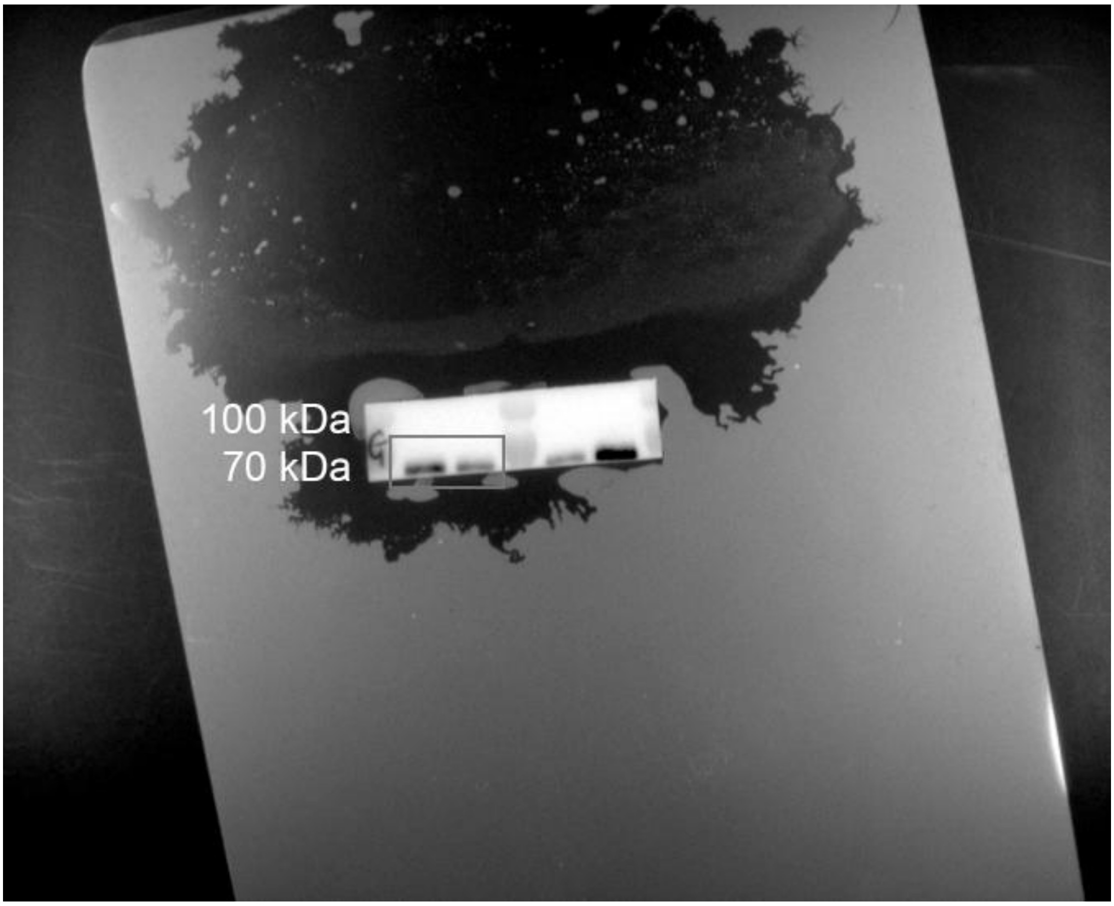

SMAD3:

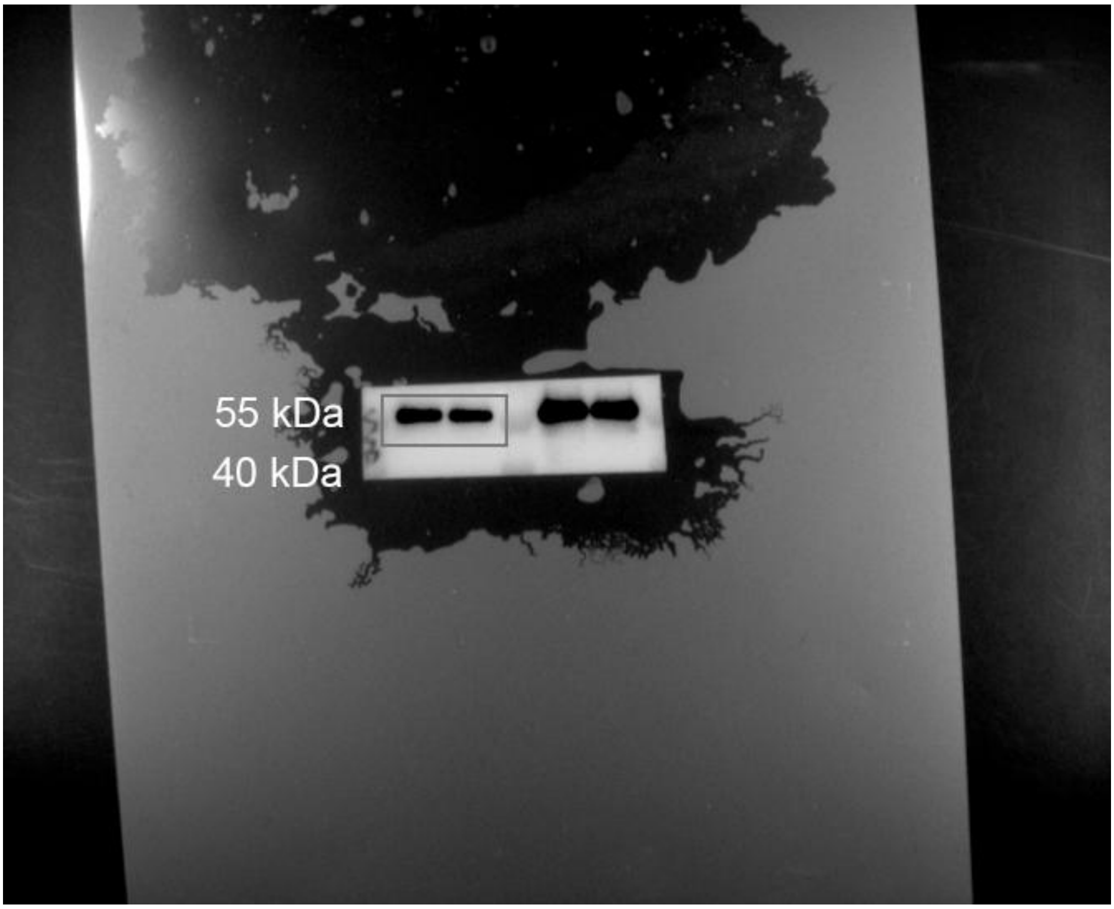

p-SMAD3:

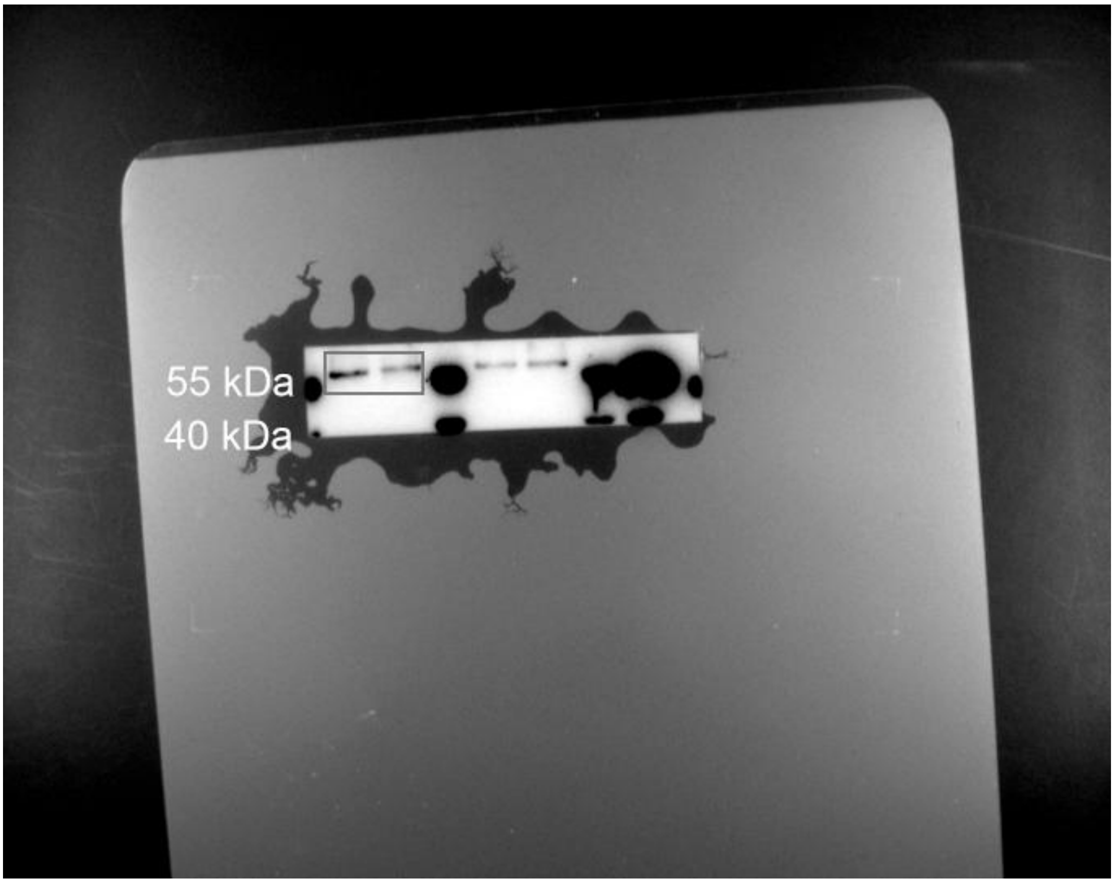

Figure 4, Source Data 1. Original membranes corresponding to Figure 4, panel B.

Lanes 1 and 2 correspond to PD14 ovaries from Ctrl and cKO mice, respectively. Lanes 4 and 5 contain samples unrelated to this experiment. The 180 kDa Plus Prestained Protein Marker (Vazyme MP201) was employed.
