# Supplementary material for: MATR3 is essential for oocyte growth and maturation quality through a dual molecular mechanism
Source: eLife. 2026 Sep 21;15:RP110703. doi: 10.7554/eLife.110703 (PMC13592816; doi:10.7554/eLife.110703)
Supplement: Supplementary file 20 [file elife-110703-fig5-data1.zip › Figure 5-source data 1.pdf]

MATR3:

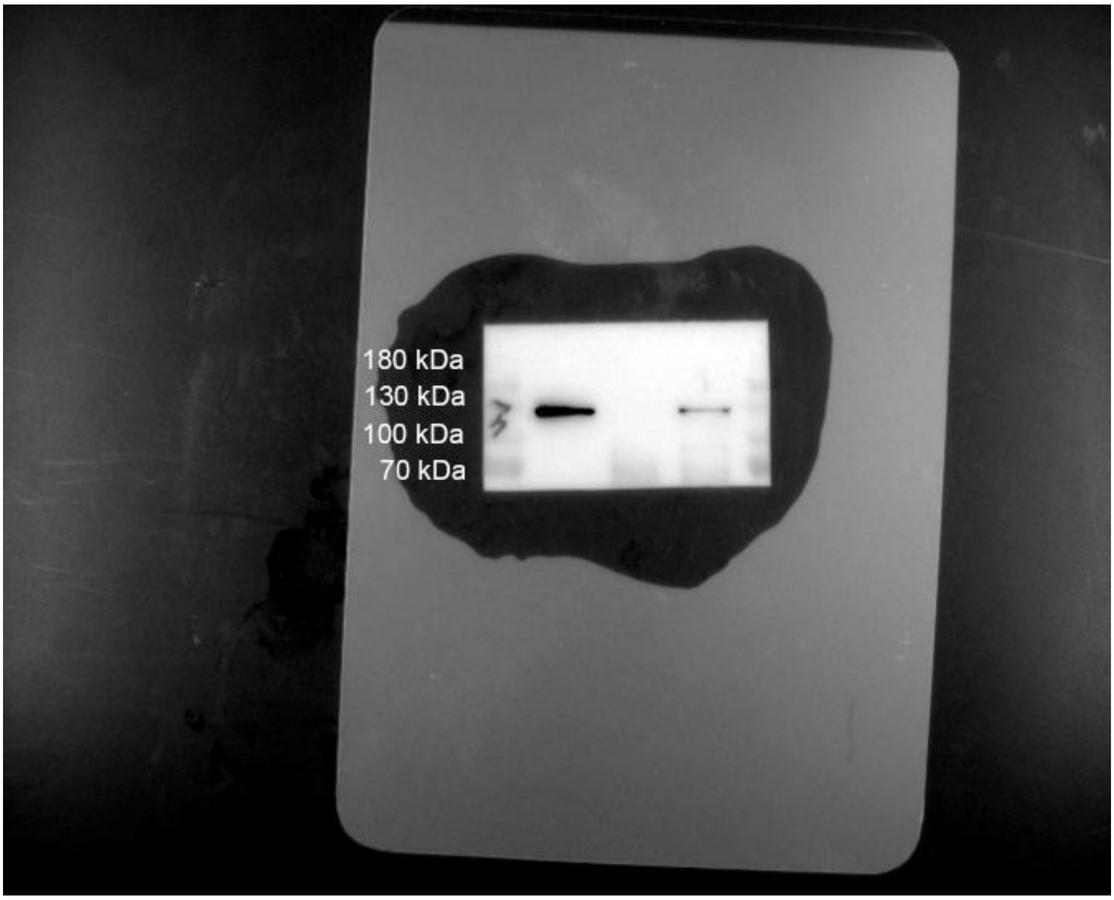

YTHDC1:

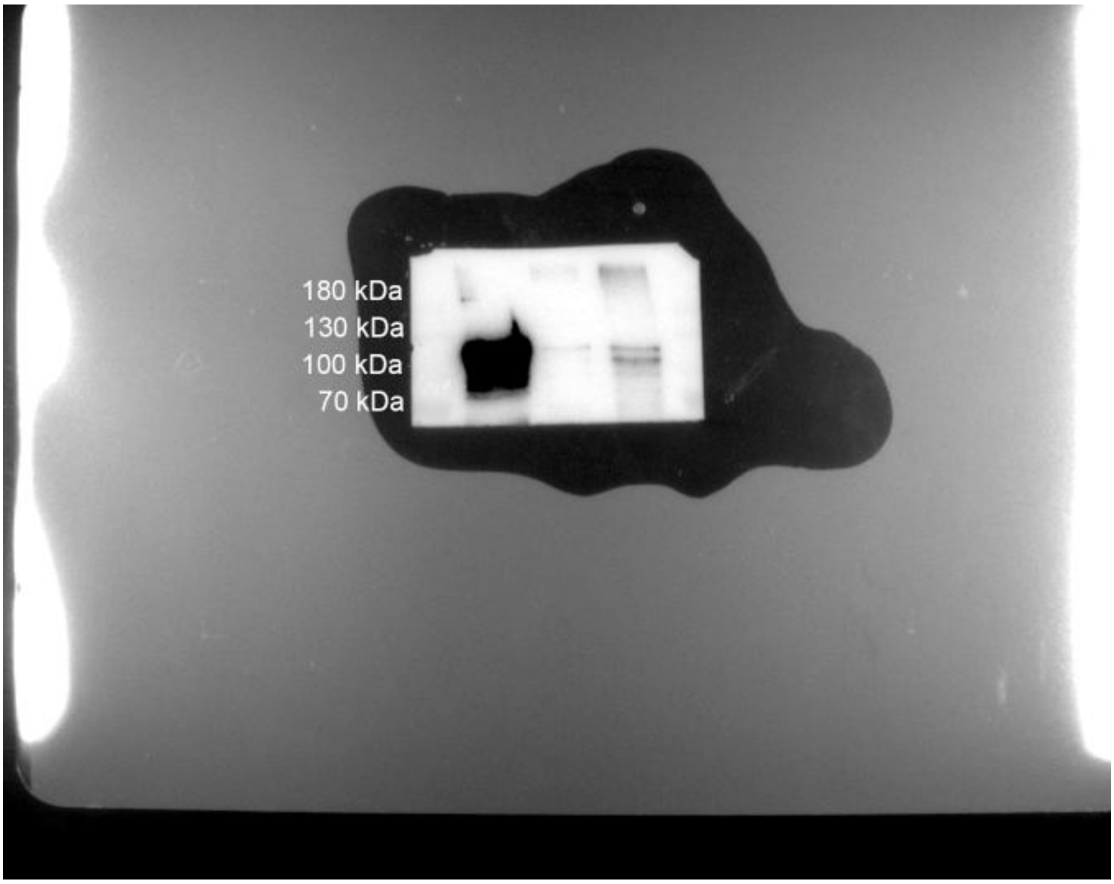

KDM3B:

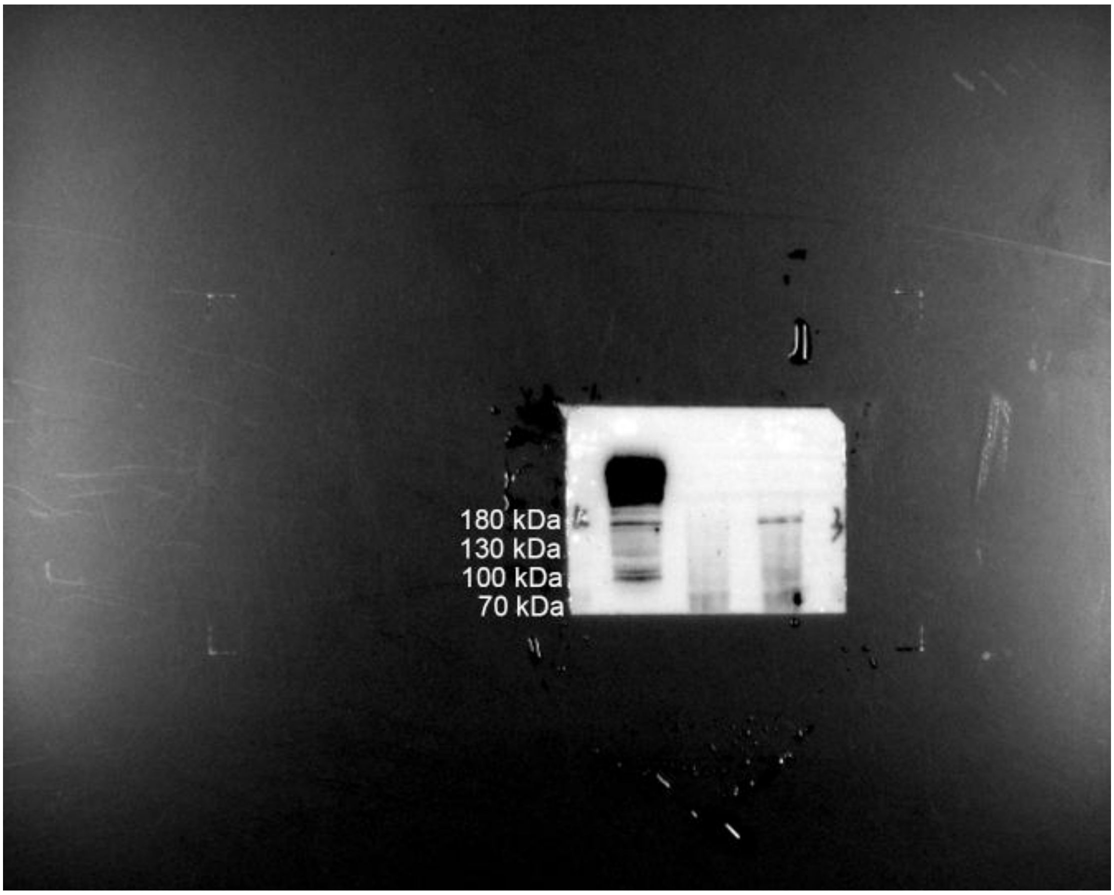

Figure 5, Source Data 1. Original membranes corresponding to Figure 5, panel C.
Lanes correspond to Input, IgG and IP samples, respectively. The Input Lane represents 10% of the total cell lysate reserved before immunoprecipitation (positive control). The IgG lane represents the negative control using a non-specific IgG antibody. The IP lane represents the precipitated complex using an anti-MATR3 antibody. The 180 kDa Plus Prestained Protein Marker (Vazyme MP201) was employed.
