# Supplementary material for: MATR3 is essential for oocyte growth and maturation quality through a dual molecular mechanism
Source: eLife. 2026 Sep 21;15:RP110703. doi: 10.7554/eLife.110703 (PMC13592816; doi:10.7554/eLife.110703)
Supplement: Supplementary file 24 [file elife-110703-fig5-figsupp1-data1.zip › Figure 5-figure supplement 1-source data 1.pdf]

β-Actin:

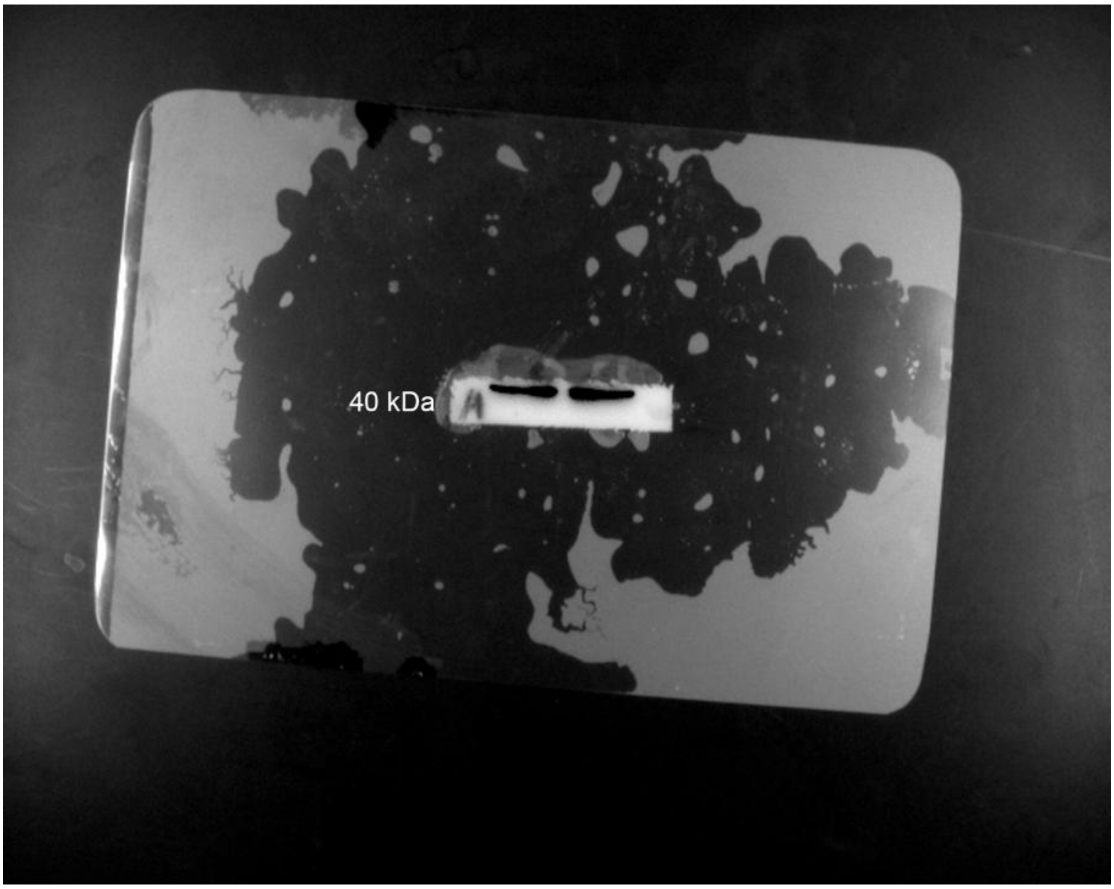

KDM3B:

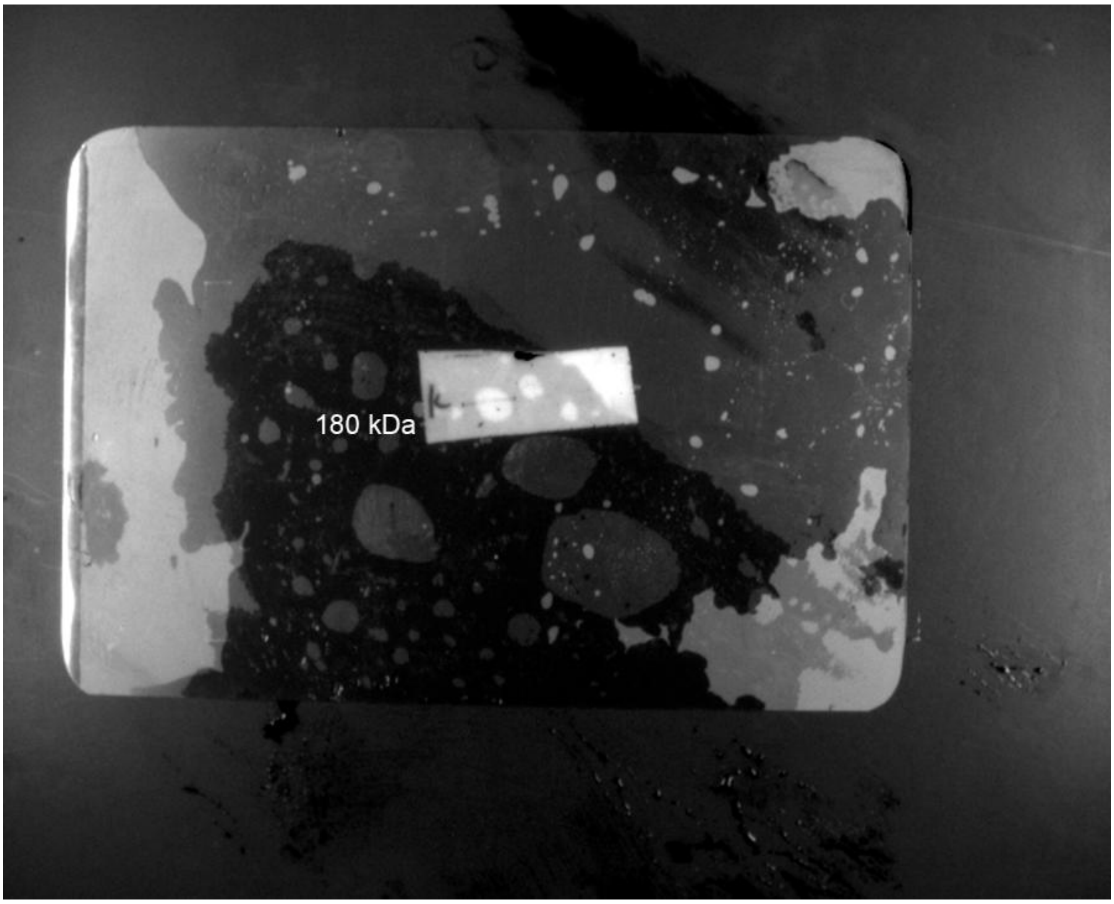

Figure 5-figure supplement 1, Source Data 1. Original membranes corresponding to Figure 5-figure supplement 1, panel C.

Lanes correspond to oocytes at the GO and FGO, with 200 oocytes loaded per lane. The 180 kDa Plus Prestained Protein Marker (Vazyme MP201) was employed.
