# Supplementary material for: MATR3 is essential for oocyte growth and maturation quality through a dual molecular mechanism
Source: eLife. 2026 Sep 21;15:RP110703. doi: 10.7554/eLife.110703 (PMC13592816; doi:10.7554/eLife.110703)
Supplement: Supplementary file 26 [file elife-110703-fig5-figsupp2-data1.zip › Figure 5-figure supplement 2-source data 1.pdf]

β-Actin:

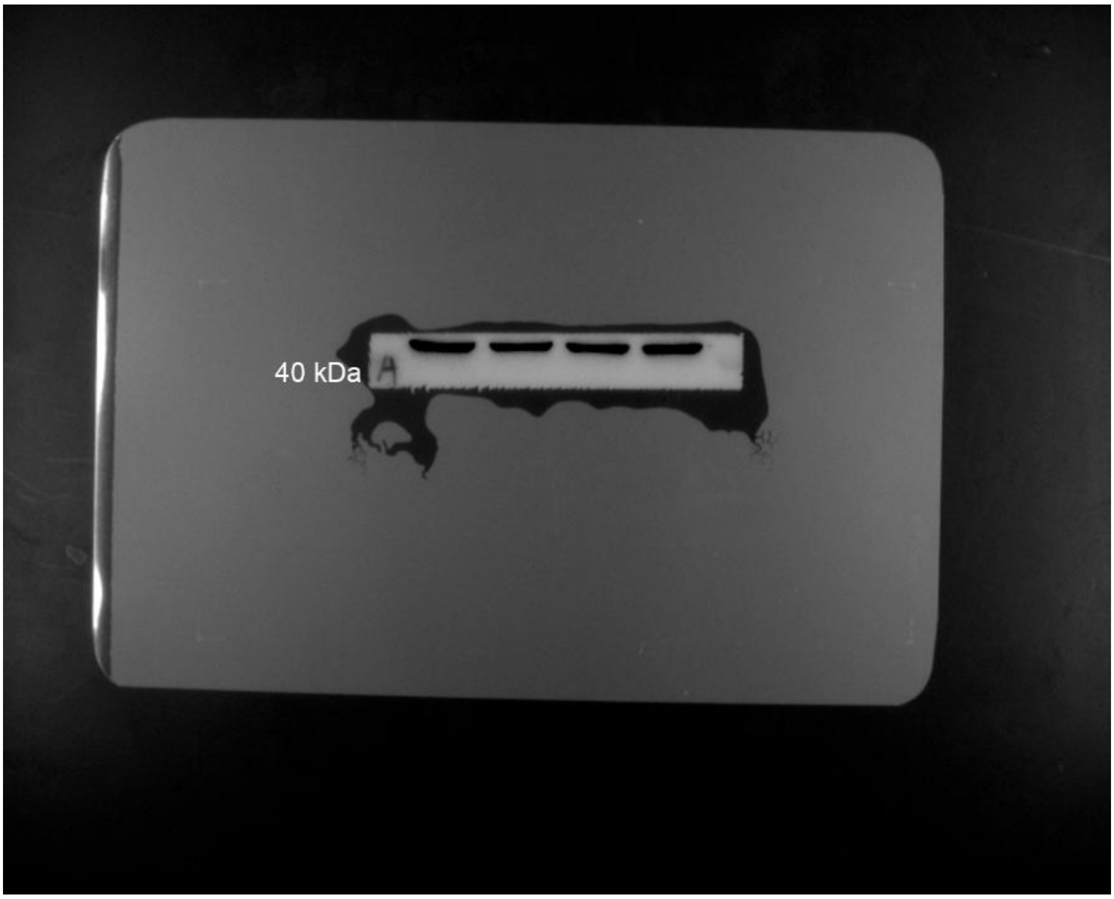

GFP：

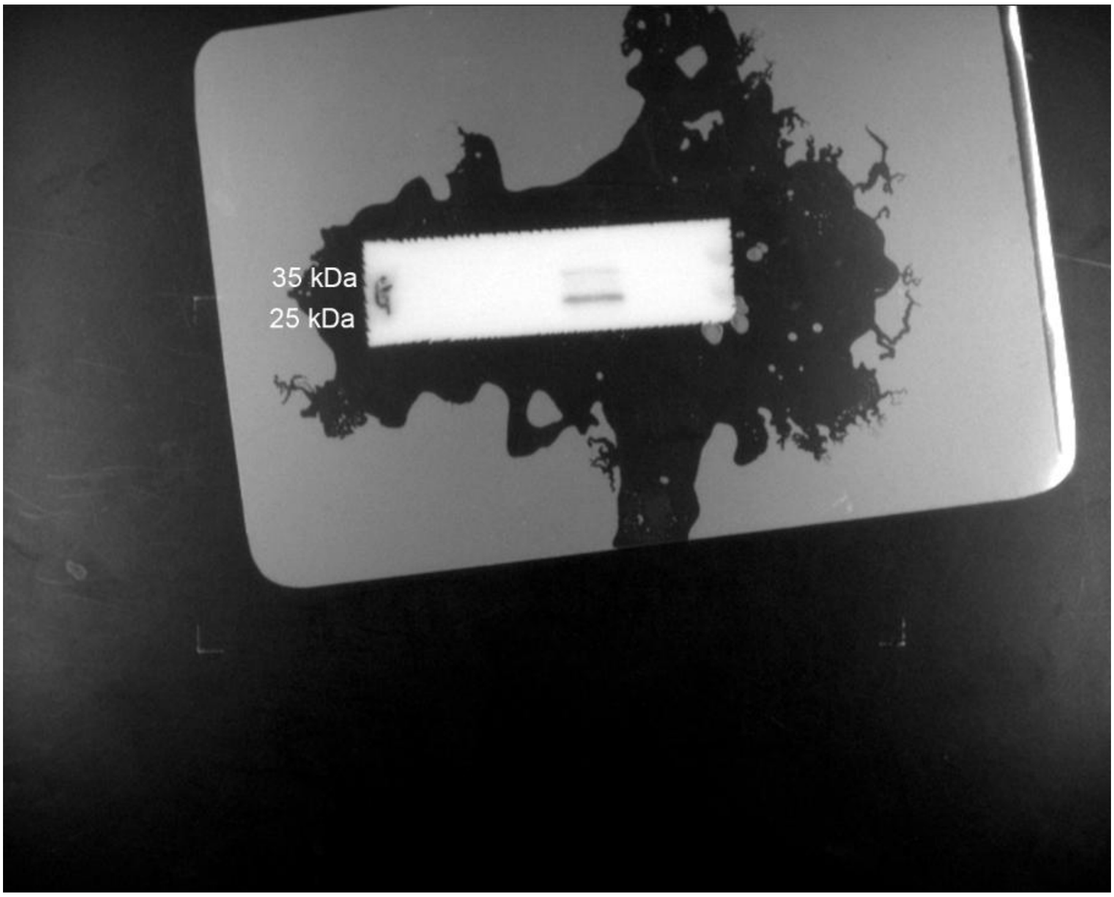

MATR3:

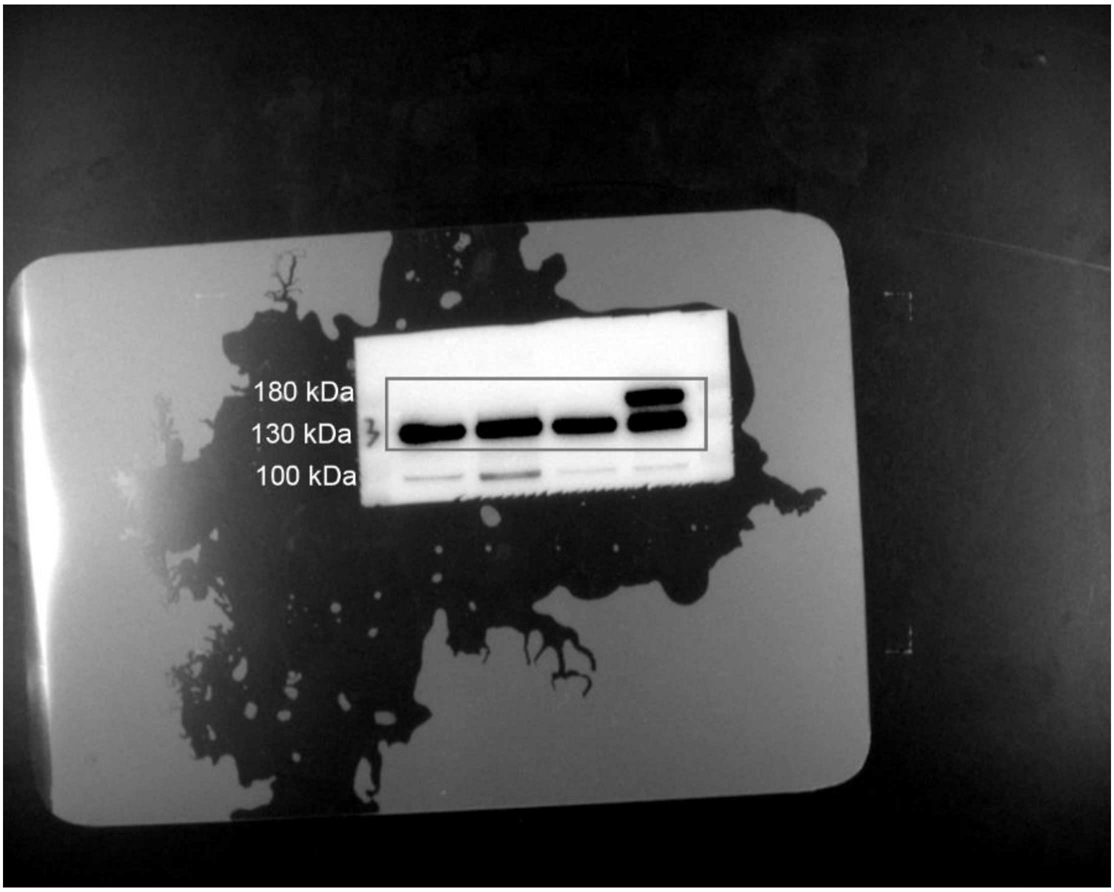

Figure 5-figure supplement 2, Source Data 1. Original membranes corresponding to Figure 5-figure supplement 2, panel A.

Lanes correspond to cells transfected with Negative Control, MATR3 plasmid, EGFP plasmid, and MATR3-EGFP plasmid for 48 h. The 180 kDa Plus Prestained Protein Marker (Vazyme MP201) was employed.
