# Supplementary material for: MATR3 is essential for oocyte growth and maturation quality through a dual molecular mechanism
Source: eLife. 2026 Sep 21;15:RP110703. doi: 10.7554/eLife.110703 (PMC13592816; doi:10.7554/eLife.110703)
Supplement: Supplementary file 28 [file elife-110703-fig6-data1.zip › Figure 6-source data 1.pdf]

RIP-MATR3:

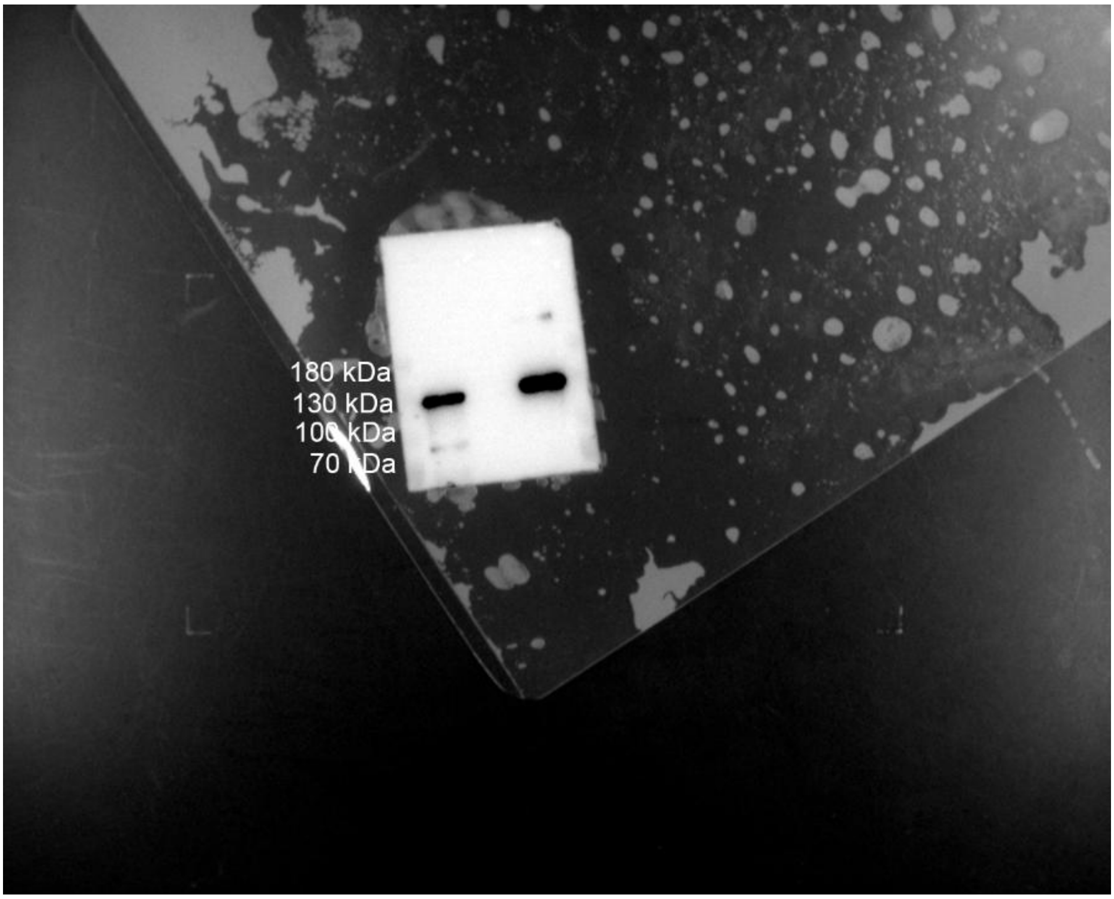

RIP-*Rdx*:

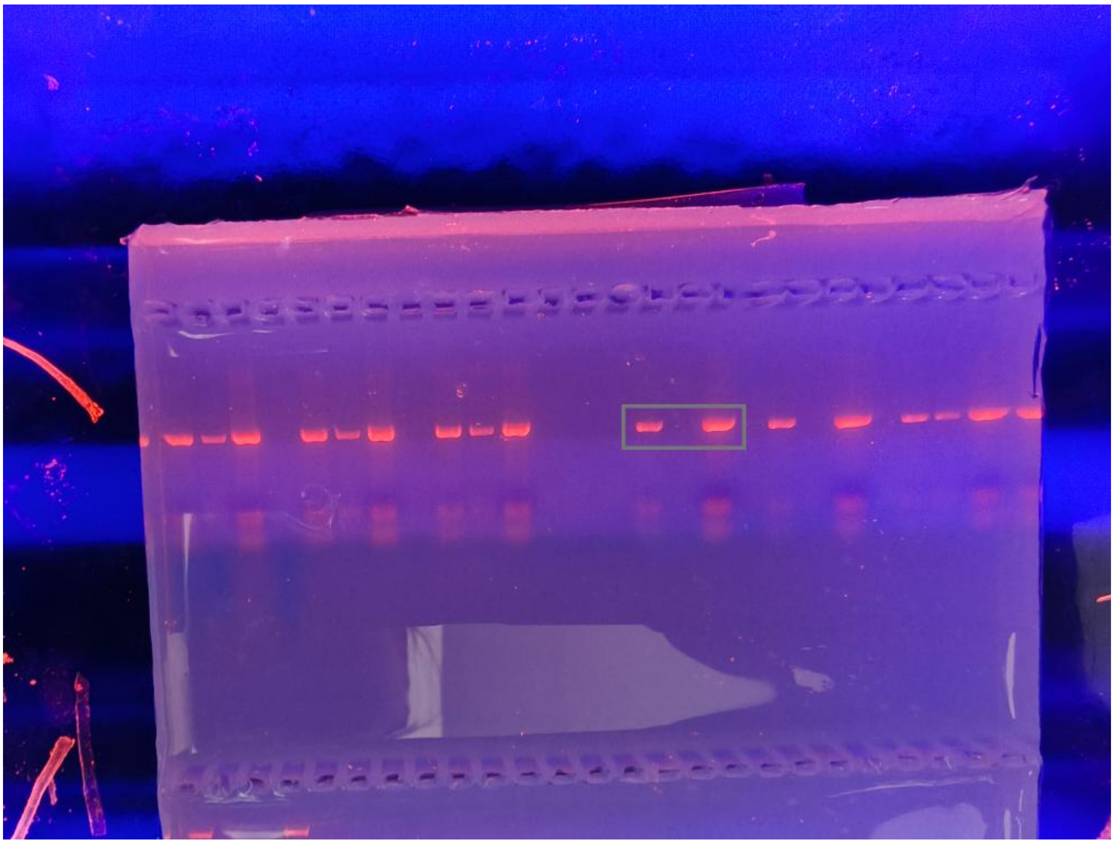

Figure 6, Source Data 1. Original membranes corresponding to Figure 6, panel I.

Lanes correspond to Input, IgG, and RIP samples. The 180 kDa Plus Prestained Protein Marker (Vazyme MP201) was employed. Western blotting validation of protein enrichment in RIP assays performed using a MATR3 antibody. Input samples represent 10% of total cell lysates before immunoprecipitation. IgG and IP samples correspond to eluted products after immunoprecipitation. Specific MATR3 protein enrichment in the IP fraction and negligible background signals in the IgG control confirm the specific and efficient pull-down of MATR3, validating the reliability of the subsequent RIP-qPCR results.

Agarose gel electrophoresis of products from RIP-qPCR assays performed using a MATR3 antibody. Lanes 14, 15, and 16 correspond to input, IgG, and RIP samples, respectively.
