# Supplementary material for: MATR3 is essential for oocyte growth and maturation quality through a dual molecular mechanism
Source: eLife. 2026 Sep 21;15:RP110703. doi: 10.7554/eLife.110703 (PMC13592816; doi:10.7554/eLife.110703)
Supplement: Peer review history

# Editor report: eLife Assessment

**DOI:** 10.7554/eLife.110703.3.sa0

**Authors:** Darryl L Russell

**Affiliations:** The University of Adelaide Australia

**Keywords:** Solid, Valuable

This **valuable** study provides insights into the role of MATR3 in oocyte maturation and folliculogenesis, using conditional knockout mice and in vitro follicle culture systems to show that MATR3 is required for oocyte growth and gene transcription, with downstream effects on follicle development. The evidence is **solid**, but some minor inadequacies in replication of key methods and independent validation reduce confidence in the conclusions. The work will be of interest to researchers in reproductive biology and fertility.

# Referee report: Reviewer #1 (Public review)

**DOI:** 10.7554/eLife.110703.3.sa1

Summary:

This study aims to clarify MATR3's function and molecular mechanism in oocyte growth and maturation, explore its association with OMA and its potential as a diagnostic and therapeutic target using specific knockout mouse models, human OMA samples and multi-omics technologies. And it has fully achieved preset objectives with results strongly supporting conclusions. Specifically, it addresses the gap in the synergistic mechanism of epigenetic and secretory signals regulated by RNA-binding proteins (RBPs) in oocyte growth and enriches the molecular etiological spectrum of oocyte maturation disorders. It is the first time to reveal the conservative function of MATR3 in multiple species, providing a paradigm for cross-species research on RBPs in the field of reproductive biology. And it provides a new candidate target for OMA, a clinically refractory infertility disease, and is expected to promote the optimization of assisted reproductive technology and the development of precision medicine.

Strengths:

The strengths of this study are significant and prominent. First, the research system is comprehensive, integrating knockout mouse models, in vitro knockdown models, multi-species (mouse, porcine and human) verification, combined with scRNA-seq, LACE-seq, CO-IP and other multi-omics and molecular biology technologies, forming a complete and progressive evidence chain. Second, the mechanism analysis is in-depth, clarifying the dual molecular mechanisms of MATR3 regulating the transcriptional synthesis and secretion of GDF9 through "recruiting KDM3B to regulate H3K9me2 demethylation" and "directly binding to Rdx mRNA", with a clear logical closed loop. Third, the clinical correlation is close. It is the first time to find abnormal nuclear localization of MATR3 in oocytes of OMA patients, providing new clues for clinical disease mechanism research, and verifying the downstream function of GDF9 through rescue experiments, effectively enhancing the translational value of the results.

Weaknesses:

This study included only one OMA patient's oocyte sample. Without clinical screening for MATR3 mutations or abnormal expression, establishing a causal relationship between MATR3 and OMA remains difficult.

# Referee report: Reviewer #2 (Public review)

**DOI:** 10.7554/eLife.110703.3.sa2

Summary:

This study investigates the role of MATR3 in oocyte development and folliculogenesis using conditional knockout mouse models together with in vitro follicle culture and molecular analyses. The authors aim to determine whether MATR3 regulates oocyte maturation and follicle development and to explore potential mechanisms linking MATR3 function to transcriptional and epigenetic regulation in growing oocytes.

Strengths:

A major strength of the work is the use of a conditional knockout mouse model combined with complementary in vitro follicle culture approaches, which together provide a useful framework for examining gene function during oocyte development. The study also attempts to integrate cellular phenotypes with molecular analyses of transcriptional activity and epigenetic markers.

Weaknesses:

Several weaknesses limit the strength of the conclusions. These include insufficient validation of key experimental manipulations (such as the efficiency of MATR3 knockdown in siRNA experiments), limited quantification or statistical analysis for some datasets, inconsistencies between the text and presented data in certain figures, and incomplete methodological descriptions that make it difficult to fully evaluate reproducibility.

Comments on revised version.

Thank you for submitting the revised manuscript. I believe the revisions have substantially improved the quality and clarity of the study, and the authors have addressed the major concerns raised during the initial review.

# Referee report: Reviewer #3 (Public review)

**DOI:** 10.7554/eLife.110703.3.sa3

Summary:

The study aims to elucidate the dual molecular mechanisms of the RNA-binding protein MATR3 in oocyte growth and maturation. The authors propose that MATR3, highly expressed in growing oocytes (GOs), regulates oocyte quality through two pathways: epigenetically, by recruiting KDM3B to remove the repressive H3K9me2 mark at the Gdf9 locus to activate transcription; and post-transcriptionally, by binding Rdx mRNA to maintain microvillus structure for GDF9 secretion. This mechanism ensures oocyte-granulosa cell communication and female fertility. The study also explores the link between MATR3 and human oocyte maturation arrest (OMA).

Strengths:

The study proposes an innovative dual-mechanism model encompassing "epigenetic transcriptional activation and cytoskeletal regulation," which not only expands the functional understanding of RNA-binding proteins in chromatin regulation but also reveals the coordination between nuclear transcription and organelle structure. By integrating scRNA-seq and LACE-seq, the authors constructed a comprehensive regulatory network for MATR3, identifying both key targets and numerous potential molecules, thereby providing rich resources for future mechanistic studies. Furthermore, the inclusion of oocyte samples from human OMA patients directly links the basic findings to clinical reproductive disorders. Despite the limited sample size, this approach demonstrates strong translational potential.

Weaknesses:

The partial phenotypic improvement achieved by exogenous GDF9 supplementation suggests that the downstream effector pathways may involve a more complex network regulation, implying that the current interpretation of GDF9 central role could be further explored. Regarding the developmental abnormalities of granulosa cells in the conditional knockout model, their pathological origins require in-depth analysis to determine whether they represent primary alterations or secondary adaptive responses resulting from the loss of oocyte signaling.

# Author comment: Author response

**DOI:** 10.7554/eLife.110703.3.sa4

**Authors:** Yibing Bao, Zhenzi Zuo, Tengteng Wang, Lin Lin, Meng Gao, Shaogang Qin, Qingfeng Yang, Bingying Liu, Wanyuan Sun, Jie Ma, Tianhua Zhu, Guoliang Xia, Bo Zhou, Rong Hu, Hua Zhang, Fengchao Wang, Chao Wang

**Affiliations:** China Agricultural University Beijing China; China Agricultural University Beijing China; China Agricultural University Beijing China; China Agricultural University Beijing China; China Agricultural University Beijing China; China Agricultural University Beijing China; China Agricultural University Beijing China; China Agricultural University Beijing China; China Agricultural University Beijing China; Ningxia Medical University General Hospital Yinchuan China; China Agricultural University Beijing China; China Agricultural University Beijing China; China Agricultural University Beijing China; Ningxia Medical University General Hospital Ningxia China; China Agricultural University Beijing China; National Institute of Biological Sciences, Beijing Beijing China; China Agricultural University Beijing China

The following is the authors’ response to the original reviews.

**Public Reviews:**

**Reviewer #1 (Public review):**

Summary:

This study aims to clarify MATR3's function and molecular mechanism in oocyte growth and maturation, explore its association with OMA, and its potential as a diagnostic and therapeutic target using specific knockout mouse models, human OMA samples, and multi-omics technologies. And it has fully achieved preset objectives with results strongly supporting conclusions. Specifically, it addresses the gap in the synergistic mechanism of epigenetic and secretory signals regulated by RNA-binding proteins (RBPs) in oocyte growth and enriches the molecular etiological spectrum of oocyte maturation disorders. It is the first time the conservative function of MATR3 has been revealed in multiple species, providing a paradigm for cross-species research on RBPs in the field of reproductive biology. It also provides a new candidate target for OMA, a clinically refractory infertility disease, and is expected to promote the optimization of assisted reproductive technology and the development of precision medicine.

Strengths:

The strengths of this study are significant and prominent. First, the research system is comprehensive, integrating knockout mouse models, in vitro knockdown models, multi-species (mouse, porcine, and human) verification, combined with scRNA-seq, LACE-seq, CO-IP, and other multi-omics and molecular biology technologies, forming a complete and progressive evidence chain. Second, the mechanism analysis is in-depth, clarifying the dual molecular mechanisms of MATR3 regulating the transcriptional synthesis and secretion of GDF9 through "recruiting KDM3B to regulate H3K9me2 demethylation" and "directly binding to Rdx mRNA", with a clear logical closed loop. Third, the clinical correlation is close. It is the first time to find abnormal nuclear localization of MATR3 in oocytes of OMA patients, providing new clues for clinical disease mechanism research, and verifying the downstream function of GDF9 through rescue experiments, effectively enhancing the translational value of the results.

Weaknesses:

This study included only one OMA patient's oocyte sample. Without clinical screening for MATR3 mutations or abnormal expression, establishing a causal relationship between MATR3 and OMA remains difficult.

We greatly appreciate positive comments and constructive feedback on our manuscript.

We are encouraged that you recognize the novelty, rigour, and clinical relevance of our study on MATR3 in oocyte development and OMA. We have carefully considered your comments and revised the manuscript accordingly. We will further expand the OMA patient cohort in future studies to verify the causal relationship between MATR3 and OMA.

**Reviewer #2 (Public review):**

Summary:

This study investigates the role of MATR3 in oocyte development and folliculogenesis using conditional knockout mouse models together with in vitro follicle culture and molecular analyses. The authors aim to determine whether MATR3 regulates oocyte maturation and follicle development and to explore potential mechanisms linking MATR3 function to transcriptional and epigenetic regulation in growing oocytes.

Strengths:

A major strength of the work is the use of a conditional knockout mouse model combined with complementary in vitro follicle culture approaches, which together provide a useful framework for examining gene function during oocyte development. The study also attempts to integrate cellular phenotypes with molecular analyses of transcriptional activity and epigenetic markers.

Weaknesses:

Several weaknesses limit the strength of the conclusions. These include insufficient validation of key experimental manipulations (such as the efficiency of MATR3 knockdown in siRNA experiments), limited quantification or statistical analysis for some datasets, inconsistencies between the text and presented data in certain figures, and incomplete methodological descriptions that make it difficult to fully evaluate reproducibility.

We greatly appreciate your constructive comments and suggestions. We are grateful for the recognition of our conditional knockout mouse model and experimental design. We have carefully addressed all the weaknesses mentioned by the reviewer, including the validation of key experiments, quantitative and statistical analysis, consistency between text and figures, and detailed methodological descriptions. Details are described point-by-point below.

**Reviewer #3 (Public review):**

Summary:

The study aims to elucidate the dual molecular mechanisms of the RNA-binding protein MATR3 in oocyte growth and maturation. The authors propose that MATR3, highly expressed in growing oocytes (GOs), regulates oocyte quality through two pathways: epigenetically, by recruiting KDM3B to remove the repressive H3K9me2 mark at the Gdf9 locus to activate transcription; and post-transcriptionally, by binding Rdx mRNA to maintain microvillus structure for GDF9 secretion. This mechanism ensures oocyte-granulosa cell communication and female fertility. The study also explores the link between MATR3 and human oocyte maturation arrest (OMA).

Strengths:

The study proposes an innovative dual-mechanism model encompassing "epigenetic transcriptional activation and cytoskeletal regulation," which not only expands the functional understanding of RNA-binding proteins in chromatin regulation but also reveals the coordination between nuclear transcription and organelle structure. By integrating scRNA-seq and LACE-seq, the authors constructed a comprehensive regulatory network for MATR3, identifying both key targets and numerous potential molecules, thereby providing rich resources for future mechanistic studies. Furthermore, the inclusion of oocyte samples from human OMA patients directly links the basic findings to clinical reproductive disorders. Despite the limited sample size, this approach demonstrates strong translational potential.

Weaknesses:

The partial phenotypic improvement achieved by exogenous GDF9 supplementation suggests that the downstream effector pathways may involve a more complex network regulation, implying that the current interpretation of GDF9's central role could be further explored. Regarding the developmental abnormalities of granulosa cells in the conditional knockout model, their pathological origins require in-depth analysis to determine whether they represent primary alterations or secondary adaptive responses resulting from the loss of oocyte signaling.

We greatly appreciate your positive and insightful comments on our study. We are grateful for the recognition of our novel dual-mechanism model, comprehensive multi-omics analysis, and translational potential from basic research to clinical OMA. We have carefully addressed the weaknesses raised by the reviewer, including in-depth discussion of the GDF9-centered regulatory network and clarification of the origin of granulosa cell abnormalities. More details point-by-point responses are provided below.

**Recommendations for the authors:**

Point-by-point responses to reviewers’ comments

We thank the reviewer very much for his/her reviewing of our work, and we appreciate the constructive comments and suggestions that have helped us to prepare an improved revision. Based on the comments of the reviewer, we have carefully revised the manuscript by performing some new experiments.

**Reviewer #1 (Recommendations for the authors):**

(1) Did most of the follicles cultured in vitro reach the antral follicle stage after 6 days?

We greatly appreciate your insightful question. We statistically analyzed the survival rate and antral follicle ratio of in vitro-cultured follicles after 6 days of culture. Due to differences in culture systems and protocols, the follicle survival rate in our study (57.43 ± 3.11%) was different from that reported in previous literature (92 ± 10%). However, the proportion of antral follicles among surviving follicles was highly consistent between our results and published data (83 ± 13% vs 80.87 ± 3.27%) (Cortvrindt and Smitz 2002).

**Author response image 1.** Ratio and survival rate of antral follicles after 6 days of culture. n = 3. Data are represented as mean ± SD.

(2) In Figure 2F, at which stage did MATR3 begin to affect oocyte diameter?

Thank you for your careful observation. Our morphological analysis of oocytes collected from PD14 and PD23 mice showed no significant difference in oocyte diameter between the cKO and Ctrl groups at the GO stage (Fig. S3D, E). However, oocytes in the cKO group became significantly smaller than those in the Ctrl group once they reached the FGO stage (Fig. 2E, F). Taken together, these results indicate that the growth defect caused by MATR3 deletion begins to manifest during the transition from the GO to FGO stage, with significant reduction in oocyte diameter clearly observed at the FGO stage as shown in Figure 2F.

(3) What was the developmental potential of oocytes in Matr3-knockout mice?

Thank you for this important question. Compared with the Ctrl group, oocytes derived from cKO mice showed a drastically reduced fertilization rate (91.55 ± 1.96% vs 10.55 ± 4.78%) and almost completely failed to develop to the blastocyst stage (76.62 ± 7.56% vs 3.67 ± 3.38%). These results clearly demonstrate that maternal deletion of *Matr3* severely compromises the developmental potential of mouse oocytes, including fertilization capacity and subsequent early embryonic development.

**Author response image 2.** Results of in vitro fertilization of oocytes. 2-cell: 2 days after fertilization; blastocyst: 4 days after fertilization. Data are represented as mean ± SD. ***p<0.001.

(4) The legend labels in the figures should not be bold.

Thank you for your valuable suggestion. We have revised all the figures accordingly.

**Reviewer #2 (Recommendations for the authors):**

This manuscript investigates the role of MATR3 in oocyte development and folliculogenesis using conditional knockout (cKO) mouse models combined with in vitro follicle culture approaches. The topic is relevant to the field of reproductive biology and provides potentially important insights into the molecular mechanisms regulating oocyte maturation and follicle development.

While the study presents interesting observations and utilizes both in vivo and in vitro experimental systems, several issues need to be addressed before the manuscript can meet the expected standards. These include concerns related to data interpretation, validation of experimental approaches, completeness of methodological descriptions, and clarity in data presentation. In addition, the manuscript requires substantial language editing to improve clarity and readability.

The comments below outline major issues that should be addressed to strengthen the manuscript, as well as specific minor points regarding presentation and clarity.

(1) The manuscript requires substantial revision to improve the written language and grammar. Numerous sentences are unclear or awkwardly phrased, which makes interpretation of the results difficult in several sections. The authors are strongly encouraged to have the manuscript professionally edited or thoroughly revised for language and clarity before making a resubmission.

We sincerely appreciate the careful and constructive comments on the language quality and clarity of the manuscript. We fully agree that the written language, grammar, and sentence structure need substantial improvement to ensure the results are presented clearly and accurately.

To address these concerns thoroughly, we have carefully revised the entire manuscript, including correcting grammatical errors, refining awkward phrasing, and restructuring unclear sentences to enhance readability and logical flow. In addition, we have sought professional language editing support to further polish the English expression and ensure the manuscript meets the linguistic standards of the journal.

All revisions related to language and clarity have been completed, and we believe the revised version is significantly improved in terms of readability and precision.

(2) Interpretation of oocyte maturation results (Line 140; Figure 2E, H). The manuscript states: "During in vitro maturation, oocytes isolated from PD23 cKO mice could not develop to metaphase II (Fig. 2E, H)." However, Figure 2H appears to show that a small proportion of knockout oocytes do reach the MII stage. Therefore, the description in the text seems inconsistent with the data presented. The authors should clarify the exact maturation rates in both groups, revise the text to accurately reflect the data, and provide statistical analysis to support the stated conclusions.

Thank you for your valuable comment. A small proportion of knockout oocytes from PD23 cKO mice can indeed develop to the MII stage. We have revised the corresponding description and supplemented the statistical analysis of maturation rates to support our conclusion.

Line 140: “During in vitro maturation, oocytes isolated from PD23 cKO mice could not develop to metaphase II (Fig.2E, H).” have been replaced by “During in vitro maturation, the proportion of oocytes from PD23 cKO mice developing to metaphase II stage was significantly reduced (Fig.2E, H, 54.9±2.08% vs 9.57±1.11%).”

(3) Human oocyte sample size: In Figure 1D, it is unclear how many human oocytes were analyzed. It is important to specify the sample size (n) for all experiments. The authors should clearly indicate the number of oocytes analyzed in this experiment. Provide this information either in the figure legend or in the main text.

Thank you for this important comment. We agree that the altered subcellular localization of MATR3 in human OMA oocytes is of great physiological significance for understanding the functional role of MATR3 during oocyte development.

Unfortunately, during a 3‑month period of sample collection, we examined MATR3 localization in immature oocytes that failed to reach the MII stage, obtained from 11 women undergoing IVF treatment. Among these samples, only one donor’s oocytes exhibited the NSN chromatin configuration. Excitingly, these NSN‑stage oocytes from this donor clearly showed the loss of MATR3 nuclear localization, which strongly supports the critical role of MATR3 during oocyte growth and maturation. We have now clearly stated the sample size (n = 11) in the figure legend and main text as suggested. In future studies, we will continue to collect more human oocyte samples to further validate these observations with an expanded sample size.

(4) Figure annotation issue: The figure legend for Figure 1F refers to an arrow, but no arrow is visible in the figure panel. Please correct this inconsistency by either adding the appropriate arrow to the figure or revising the legend accordingly.

Thank you for pointing out this error. We have revised the figure legend for Figure 1F accordingly to correct this inconsistency.

(5) Description of follicle analysis (Line 146): The sentence: "This was reinforced by the data of available follicles within the follicles of mice on PD35 (Fig. 2I, J)." is incorrect or poorly phrased. It should likely read: "...available follicles within the ovaries of mice at PD35...".

We really appreciate your constructive suggestion on the phrasing. We have revised this sentence in the revised manuscript accordingly.

(6) Quantification of proliferating cells: Figure 2K shows Ki-positive cells, but quantitative analysis is not provided. The authors should quantify the number or proportion of Ki-positive cells in both control and cKO groups and include statistical analysis to support any claims regarding differences in proliferation.

Thank you for your valuable suggestion. We have quantified the number of Ki‑67‑positive granulosa cells in both control and cKO groups and performed the corresponding statistical analysis.

The quantitative results have been added to Fig. S3G, and the relevant description has been supplemented in the main text at Line 150 to support our conclusion regarding cell proliferation differences.

Line 150: “Consistently, immunofluorescence staining showed that the numbers of Ki67-positive (Fig. 2K) in cKO mice were lower than those found in the Ctrl.” have been replaced by “Consistently, immunofluorescence staining showed that the numbers of Ki67-positive (Fig. 2K, Fig. S3G) in cKO mice were lower than those found in the Ctrl (73.55±13.29% vs 24.65±7.80%).”

(7) Validation of findings in the in vivo cKO model (Figure 3): The development of an in vitro follicle culture system is an interesting and valuable component of the study. However, several key analyses performed in vitro (e.g., transcription assays and analysis of epigenetic markers) should ideally also be validated in oocytes derived from the in vivo cKO model.

Thanks for the valuable concern. We fully agree with you that the in vivo cKO model should be used to validate several key analyses performed in vitro. We collected growing oocytes from Ctrl and cKO mice and conducted transcription assays as well as analysis of epigenetic markers. The results showed that *Matr3* knockout significantly downregulated transcriptional activity in GO and increased H3K9me2 levels (Author response image 3), which is consistent with our in vitro findings (Fig 3D E I J). These in vivo results confirm that MATR3 plays a critical role in regulating GO transcriptional activity and H3K9me2 levels.

**Author response image 3.** *Matr3* knockout results in the reduction of transcriptional activity. (A) EU staining (green) in GO collected from Ctrl and cKO. n = 15. (B) Quantification of the mean fluorescence intensity of EU in oocytes. (C) H3K9me2 staining (red) in GO collected from Ctrl and cKO. n = 15. (D) Quantification of the mean fluorescence intensity of H3K9me2 in oocytes. Scale bar: 20 μm. Data are represented as mean ± S.D. ***P < 0.001.

(8) To strengthen the conclusions, the authors should consider repeating key experiments using oocytes directly isolated from the cKO mice. This would help confirm that the observed effects are not artifacts of the in vitro culture system.

Thank you for this valuable and constructive suggestion. We fully agree that the conditional knockout mouse model is essential for verifying the physiological significance of MATR3 in vivo.

To address this point, we have validated multiple key in vitro findings using oocytes directly isolated from cKO mice. For instances, the changes in oocyte transcriptional activity (EU staining) (in Comments 7), H3K9me2 levels (in Comments 7), GDF9 levels (Fig 4.B C E F), and OO-Mvi (Fig 6.A B, Author response image 4) all showed consistent trends with our in vitro knockdown results. In addition, the complete infertility phenotype of cKO female mice further demonstrates that MATR3 is indispensable for oocyte growth and meiotic maturation. We have also provided supplemental data from GDF9 rescue experiments and sequencing analysis performed in the mouse model.

**Author response image 4.** *Matr3* knockdown impairs the structural integrity of oocyte OO-MVi. (A) p-ERM staining (green) showing the OO-Mvi in oocyte from NC and si-*Matr3*. (B) Quantification of the number of Oo-Mvi vesicles (n = 6). Scale bar: 20 μm. Data are represented as mean ± SD. ***P < 0.001.

In conclusion, the core conclusions of this study are supported by the mutual validation of key experimental results from MATR3-specific knockdown in vitro and *Matr3* conditional knockout mouse models in vivo.

(9) (1) Validation of MATR3 knockdown: The in vitro MATR3 knockdown experiment presented in Figure 4G raises an important concern: it is unclear whether Matr3 knockdown was effectively achieved in the oocytes analyzed. The authors should provide direct evidence of knockdown efficiency, for example, immunostaining for MATR3 protein on the oocytes. Without such validation, it is difficult to interpret the functional outcomes observed.

As requested, we have provided direct evidences of the knockdown efficiency via immunostaining, which is now presented in Fig.S2D.

In this experiment, oocytes from early growing follicles (approximately 150 μm in diameter) were microinjected with *Matr3* siRNA. Following 5 days of continuous in vitro culture, oocytes from both the NC and si-*Matr3* groups were isolated and subjected to immunofluorescence staining to assess protein levels. As shown in the figure, the oocytes at this stage exhibited the characteristic non-surrounded nucleolus (NSN) chromatin configuration. We observed robust MATR3 protein expression within the nucleus of NC oocytes, whereas the MATR3 protein levels were markedly reduced in the si-*Matr3* group. These results confirm the successful construction of the *Matr3* knockdown model in early growing follicle oocytes.

(9) (2) Furthermore, it would be more convincing if the authors could perform the Gdf9 supplementation experiments using follicles isolated from the cKO mice, rather than relying solely on siRNA knockdown in vitro. Such experiments would provide clearer and more physiologically relevant evidence. If these experiments were attempted but did not produce similar results, this should be discussed.

Thank you for your valuable and insightful suggestion. We fully agree that performing GDF9 supplementation experiments using follicles isolated from cKO mice would provide more direct and physiologically relevant evidence to strengthen our conclusions.

Unfortunately, when we attempted to conduct GDF9 rescue experiments on follicles from cKO mice, neither the control nor cKO follicles were able to develop to the antral follicle stage (n=3). We speculate that this was caused by insufficient bioactivity of the veterinary-grade FSH been used, as compared to the imported FSH been provided by NHPP. Unfortunately, this particular FSH product has been discontinued. We are currently actively seeking and attempting to purchase new, qualified FSH reagents to repeat these experiments and further validate our findings in future work.

(10) Figure citation order: Figures are not cited sequentially in the text. For example, Figure 6J is described first (line 250), followed by Figure 6A. Figures should be discussed in logical order, typically starting from panel A. Please revise the text to ensure that figure panels are introduced sequentially.

Thank you for this careful and important comment.

We have carefully revised the citation order of all figure panels in the main text, especially for Figure 6, to ensure they are introduced sequentially from panel A to the last panel in logical and numerical order, rather than being cited out of sequence.

The corresponding adjustments have been made in the revised version of the manuscript.

(11) Figure 6J interpretation: The purpose of the images shown in this figure is unclear. The authors should provide higher magnification images to clearly visualize the Oo-Mvi structures and include quantification of the observed phenotype to support the interpretation. It is important because the main findings of the paper heavily rely on these results.

Thank you for this valuable and constructive suggestion. We fully agree that higher‑magnification images and quantitative analysis are essential to clearly demonstrate the Oo‑Mvi structures and reliably support our conclusions, especially given the importance of these results to the main findings of this study.

Accordingly, we have replaced the original panels in Figure 6A with higher‑magnification images to better visualize Oo‑Mvi structures. In addition, we have supplemented the corresponding quantitative analysis of the observed phenotype to strengthen the interpretation of this figure (Fig 6B). All revisions have been incorporated into the revised manuscript.

(12) Incomplete Materials and Methods section: The Materials and Methods section lacks important experimental details required for reproducibility. Specifically, the Matr3 flox mouse model. Either provide the appropriate reference describing the Matr3 floxed mice or include details on how the floxed allele was generated.

Thank you for your valuable and careful comment. We fully agree that detailed experimental information in the Materials and Methods section is crucial for ensuring the reproducibility of the study. And we apologize for the omission of key details regarding the *Matr3* flox mouse model.

In response to your suggestion, we have thoroughly supplemented the relevant experimental details in the Materials and Methods section of the revised manuscript, including the specific construction strategy of the *Matr3* floxed allele. These detailed descriptions will enable other researchers to reproduce our mouse model and verify the experimental results.

All supplementary information has been integrated into the revised manuscript to meet the requirements of experimental reproducibility. We greatly appreciate your guidance in helping us improve the completeness and rigour of our study.

(13) Follicle isolation: The manuscript does not describe how growing follicles were isolated. Please specify whether follicles were isolated using enzymatic digestion or mechanical dissection and provide sufficient methodological detail so that other researchers can reproduce the experiments.

Thank you for this valuable comment. We agree that adding this information is essential for ensuring the reproducibility of our experiments. We have supplemented the corresponding description in the Materials and Methods section. Briefly, growing follicles were isolated by mechanical dissection using insulin syringes under a stereomicroscope, without any enzymatic digestion.

**Reviewer #3 (Recommendations for the authors):**

(1) Since KDM3B and MATR3 interact in cell lines, does this relationship affect the functional localization of KDM3B within oocytes? Specifically, does the localization of KDM3B change in cKO mice (e.g., nuclear export or aggregation)?

Thank you for this insightful and constructive question. To address whether the interaction between KDM3B and MATR3 influences the functional localization of KDM3B in oocytes, we performed immunofluorescence staining to examine the subcellular distribution of KDM3B in cKO oocytes. Our results demonstrated that the nuclear localization of KDM3B remained unaltered; no obvious nuclear export or abnormal aggregation was observed in MATR3-deficient oocytes (Author response image 5).

Based on these observations combined with our other experimental data, we propose that MATR3 regulates oocyte transcriptional activity through its physical interaction with KDM3B, rather than by controlling the nuclear targeting of KDM3B. Notably, despite unchanged nuclear localization of KDM3B in MATR3 cKO oocytes, we detected significantly elevated global levels of H3K9me2 and markedly reduced transcriptional activity (in Comments 7). These findings indicate that KDM3B loses its physiological function of demethylating H3K9me2 and promoting transcription in the absence of MATR3.

In line with this mechanism, previous results showed that KDM3B knockout in female mice leads to follicle arrest at the secondary follicle stage and consequent infertility (Liu et al. 2015). Collectively, we conclude that in MATR3 cKO oocytes, although KDM3B is properly localized in the nucleus, it fails to execute its H3K9me2 demethylase activity, thereby impairing normal transcriptional regulation during oocyte development.

**Author response image 5.** *Matr3* knockout has no effect on KDM3B localization. KDM3B staining (red) in GO collected from Ctrl and cKO. n = 50. Scale bar: 40 μm.

(2) As the GDF9 rescue experiment only partially restores the phenotype, it is suggested to select 2-3 novel targets with high binding intensity and significant expression changes from the LACE-seq data (e.g., Igf2bp2 or Ccnb1 mentioned in the text) to further illustrate that MATR3 regulates a network.

Thanks for this meaningful and constructive suggestion.

We have supplemented the relevant data in Fig. S9 and further elaborated on these findings in the Discussion section in our revised manuscript, following your advice. Briefly, we collected growing oocytes from Ctrl and cKO mice and performed RT‑qPCR analysis to verify the expression of *Igf2bp2* and *Ccnb1* - two representative novel targets with strong binding intensity and significant expression changes identified from our LACE‑seq bioinformatics analysis. The results showed that both genes were significantly downregulated in cKO oocytes compared with controls, supporting the notion that MATR3 regulates a functional RNA network during oocyte development.

(3) Are the granulosa cell defects primary or secondary? It is recommended to collect ovaries from earlier-stage cKO mice (e.g., PD7 or PD10) to examine the levels of FOXL2 and PCNA in granulosa cells.

Thank you for your valuable comment. To clarify whether the granulosa cell defects are primary or secondary, we further investigated the temporal effect of MATR3 deficiency on granulosa cells by collecting ovaries from PD7, which is a critical period for primordial follicle activation and early follicular development.

To evaluate the status of granulosa cells, we performed immunofluorescence staining on PD7 ovarian sections using FOXL2 (a specific marker for granulosa cells) to quantify the number of granulosa cells, and Ki67 (a proliferation-related marker) to assess the proliferative capacity of granulosa cells. The results, as shown in Author response image 6, demonstrated that there were no significant differences in either the number of granulosa cells or their proliferation levels in primary follicles between cKO and Ctrl.

These findings are consistent with the data in our supplementary Fig S3F, where we observed no significant differences in the number of primordial follicles and growing follicles at PD7 between the two groups. Collectively, these results indicate that the activation of primordial follicles is not affected by MATR3 deficiency in oocytes, and the impairment of granulosa cells caused by oocyte-specific *Matr3* knockout occurs at the secondary follicle stage rather than the early stages of follicular development. Therefore, we conclude that the granulosa cell defects in cKO mice are secondary to the oocyte dysfunction induced by MATR3 deficiency.

**Author response image 6.** Loss of MATR3 in oocytes does not affect the number and proliferation of granulosa cells in primordial follicles. (A) Immunohistochemistry results showing granulosa cells in PD7 ovaries from Ctrl and cKO. (B) Quantification of granulosa cell number in the largest cross-section of primary follicles. (C) Quantification of the proliferation rate of granulosa cells in the largest cross-section of primary follicles. n = 15. Data are represented as mean ± SD. n.s., not significant.

References:

(1) Cortvrindt RG, Smitz JE. 2002. Follicle culture in reproductive toxicology: a tool for in-vitro testing of ovarian function? Human reproduction update 8: 243-254.

(2) Liu Z, Chen X, Zhou S, Liao L, Jiang R, Xu J. 2015. The histone H3K9 demethylase Kdm3b is required for somatic growth and female reproductive function. International journal of biological sciences 11: 494-507.
